# *Synechocystis*: A model system for expanding the study of cyanobacterial circadian rhythms

**DOI:** 10.3389/fphys.2022.1085959

**Published:** 2023-01-04

**Authors:** Chi Zhao, Yao Xu, Bo Wang, Carl Hirschie Johnson

**Affiliations:** ^1^ Department of Biological Sciences, Vanderbilt University, Nashville, TN, United States; ^2^ Department of Chemical and Biomolecular Engineering, Vanderbilt University, Nashville, TN, United States

**Keywords:** circadian, cyanobacteria, synechocystis, synechococcus elongatus, biological clocks

## Abstract

The study of circadian rhythms in bacteria was transformed by studies of the cyanobacterium *Synechococcus elongatus*. However, in a number of respects *S. elongatus* is atypical, and while those unusual characteristics were helpful for rapid progress in the past, another commonly used cyanobacterial species, *Synechocystis* sp. PCC 6803, may be more representative and therefore more productive for future insights into bacterial clock mechanisms. In the past, circadian studies of *Synechocystis* have suffered from not having an excellent reporter of circadian gene expression, but we introduce here a new luminescence reporter that rivals the reporters that have been used so successfully in *S. elongatus*. Using this new system, we generate for the first time in *Synechocystis* circadian period mutants resulting from point mutations. The temperature compensation and dark-pulse resetting that mediates entrainment to the environment is characterized. Moreover, we analyse the complex organization of clock genes in *Synechocystis* and identify which genes are essential for circadian rhythmicity and adaptive fitness for entrainment and optimal phase alignment to environmental cycles (and which genes are not). These developments will provide impetus for new approaches towards understanding daily timekeeping mechanisms in bacteria.

## Introduction

Before the mid-1980s, chronobiologists thought that endogenous circadian rhythms were an exclusive property of eukaryotic organisms, and it became a dogma that prokaryotic organisms were either too “simple” or grew too rapidly to have evolved a *bona fide* circadian timekeeper (Johnson et al., 1996). That dogma cracked in 1986 with reports that the diazotrophic cyanobacterium *Synechococcus* RF-1 displayed daily rhythms of nitrogen fixation in LD cycles that persist in constant light (LL) (Grobbelaar et al., 1986; Huang et al., 1990; Chen et al., 1991). The salient properties of circadian rhythms–persistence, entrainment, and temperature compensation (Johnson et al., 2017) were established in these early studies, but progress on the genetics and mechanism of the cyanobacterial clockwork was hindered because no genetic tools were available for *Synechococcus* RF-1 (e.g., transformation, homologous recombination, luminescence/fluorescence reporters, *etc.*).

Several years after the reports of circadian phenomena in *Synechococcus* RF-1, we and our collaborators began the analysis of circadian rhythmicity in the cyanobacterium *Synechococcus elongatus* PCC 7942 (hereafter *S. elongatus*), which was a species for which genetic tools were available, and for which a luciferase reporter strain (P_*psbAI*_::luxAB) had already been generated for the analysis of light intensity regulation of gene expression (Kondo et al., 1993; Johnson & Xu, 2009). The availability of genetic tools and a robust luminescence reporter of rhythmic gene expression enabled spectacular progress on understanding the mechanism and adaptive significance of circadian rhythmicity in cyanobacteria, transforming *S. elongatus* into one of the best understood circadian model organisms (Johnson et al., 2017; Chavan et al., 2021; Ito-Miwa et al., 2021; Johnson & Rust, 2021).

On the other hand, the cyanobacterium *Synechocystis* sp. PCC 6803 (hereafter *Synechocystis*) has long been a model organism for photosynthesis research and biotechnological applications (Nixon et al., 1992; Pakrasi & Vermaas, 1992; Shen et al., 1993; Ikeuchi & Tabata, 2001; Jagannathan & Golbeck, 2009; Santos-Merino et al., 2019; Wang et al., 2021b) partially due to the early discovery of its natural competence for genetic transformation (Grigorieva & Shestakov, 1982). For example, *Synechocystis* has served as a model platform for the structural and functional characterization of photosynthetic mechanisms, especially of photosystem II (Nixon et al., 1992; Pakrasi & Vermaas, 1992; Larom et al., 2010). When the complete genome sequence of *Synechocystis* became available in 1996, it became among the first of all phototrophic organisms to have that wealth of genetic information (Kaneko et al., 1996; Nakamura et al., 1998). As a result of this genomic data, research productivity involving this strain dramatically accelerated. In particular, *Synechocystis*-derived strains were explored as microbial cell factories to produce a variety of fuels and chemicals (Wang, et al., 2021b), including precursors to (biodegradable) plastics, such as poly-3-hydroxybutyrate (Wu et al., 2001; Agarwal et al., 2022), 3-hydroxybutyrate (Wang et al., 2018b), 3-hydroxypropionate (Wang Y. et al., 2016), lactate (Varman et al., 2013; Angermayr et al., 2014), and ethylene (Ungerer et al., 2012; Zhu et al., 2015; Wang et al., 2021a).

However, in comparison with *S. elongatus*, *Synechocystis* has been understudied in terms of its circadian properties (Aoki & Onai, 2009; Schmelling et al., 2021). The analysis of circadian phenomena in *Synechocystis* may have been hampered by the fact that the P_*dnaK*_ *::luxAB* reporter developed for *Synechocystis* (Aoki et al., 1995) was never as robust as the P_*psbAI*_ *::luxAB* reporter that revolutionized circadian analyses in *S. elongatus* (Kondo et al., 1993; Kondo et al., 1994; Johnson & Xu, 2009; Johnson et al., 2017). Nevertheless, understanding and manipulating the circadian system of *Synechocystis* specifically would be beneficial for multiple reasons. For example, some strains of *Synechocystis* can grow photoheterotrophically by using glucose in the medium (Anderson & McIntosh, 1991), whereas *S. elongatus* is an obligate photoautotroph and must have light to grow. Therefore, photoheterotropic characteristics of *Synechocystis* could be useful in the analysis of circadian behavior in constant darkness using a luciferase reporter (Aoki et al., 1997), which is a capability that is not possible in the obligate photoautotroph *S. elongatus*. Moreover, in terms of biotechnological applications, we previously showed in *S. elongatus* that manipulating the circadian system can be used to enhance foreign gene expression (Xu et al., 2013b), and similar tools could be applied to the versatile biotech platform *Synechocystis*.

To overcome the deficit of an inadequate circadian assay, we have developed a new luminescence reporter for *Synechocystis* that exhibits many of the advantageous properties of *S. elongatus*’ P_*psbAI*_ *::luxAB* reporter in terms of brightness and excellent peak-to-trough amplitude. Surprisingly, our new reporter is based on using a promoter that is not endogenous to *Synechocystis*, but is derived from a chloroplast gene from a higher plant. We show here that the P_*psbA*_^*Ah*^::luxAB reporter as applied to *Synechocystis* identifies circadian properties that are equivalent to those that have been extensively characterized in *S. elongatus* and enabled the isolation of period mutants as well as rigorous tests of adaptive significance in *Synechocystis*.

## Results

**FIGURE 1:**
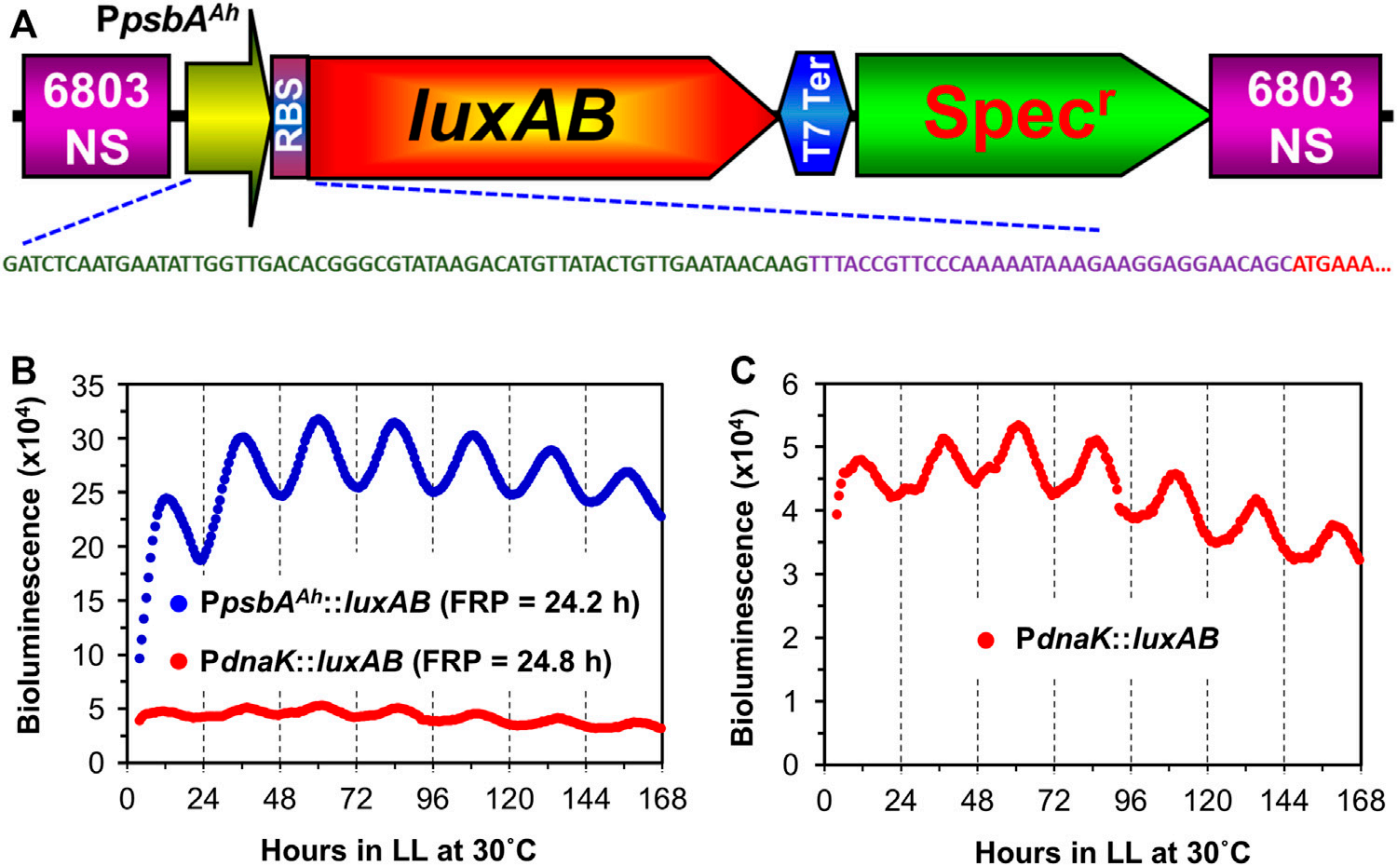
Comparison of rhythmic *luxAB* expression between the plant *psbA* promoter and the native *dnaK* promoter from colonies of *Synechocystis* sp. PCC 6803 on agar. **(A)**. Diagram of the P_*psbA*_^*Ah*^::luxAB expression construct with a spectinomycin resistance marker inserted into the genome of PCC 6803. The DNA sequence shows the nucleotide sequences of the *psbA* promoter from the chloroplast of *Amaranthus hybridus* (green), an artificial ribosomal binding site (purple), and the partial N-terminal coding region of *luxA* (red). The physical map and full nucleotide sequence of the P_*psbA*_^*Ah*^::luxAB expression plasmid is presented in Supplementary Figure S1. **(B)**. Representative luminescence rhythms and calculated free-running periods (FRPs) of the P_*psbA*_^*Ah*^::luxAB and P_*dnaK*_ *::luxAB* reporters monitored in constant light at 30°C with the Kondotron. **(C)**. The same luminescence rhythm for the P_*dnaK*_ *::luxAB* reporter shown in panel B but on an expanded scale.

A strong promoter system was developed independently for biotechnological purposes using *Synechocystis* that derived a hybrid promoter from the chloroplast *psbA* gene of the higher plant *Amaranthus hybridus* coupled to an optimized ribosome binding site (RBS) (Salis et al., 2009; Xiong et al., 2015). We tested whether this hybrid promoter would display circadian rhythms in *Synechocystis* by designing a bacterial luciferase reporter and recombining it into the genome. This hybrid promoter/reporter (P_*psbA*_^*Ah*^::luxAB) displays excellent circadian rhythms of luminescence (Figure 1). In comparison with the previously used P_*dnaK*_ *::luxAB* reporter (Aoki et al., 1995), luminescence signals are 5–10 times brighter with the P_*psbA*_^*Ah*^::luxAB reporter and display robust circadian rhythms with a period *τ*) slightly longer than 24 h in constant light (LL) at 30°C (Figure 1B, Supplementary Figure S1). As in the case of *S. elongatus*, we take this luminescence rhythm to be a reporter of pervasively rhythmic gene expression in *Synechocystis*, which has also been assessed by microarray studies (Liu et al., 1995; Woelfle et al., 2007; Ito et al., 2009; Vijayan et al., 2009). An expanded scale is shown for the data of the P_*dnaK*_ *::luxAB* reporter in Figure 1C, and this is among the most robustly rhythmic traces we have observed for this reporter, whereas the trace for the data of the P_*psbA*_^*Ah*^::luxAB reporter in Figure 1B is of average robustness. Clearly the quality of rhythms is excellent with the P_*psbA*_^*Ah*^::luxAB reporter, and its brightness is also an advantage because it is less demanding of the sensitivity of the monitoring instruments. In addition, the P_*psbA*_^*Ah*^::luxAB reporter exhibited robust rhythms over a broad range of temperatures, whereas the rhythms of P_*dnaK*_ *::luxAB* reporter became poor or arhythmic at lower temperatures (see below).

**FIGURE 2:**
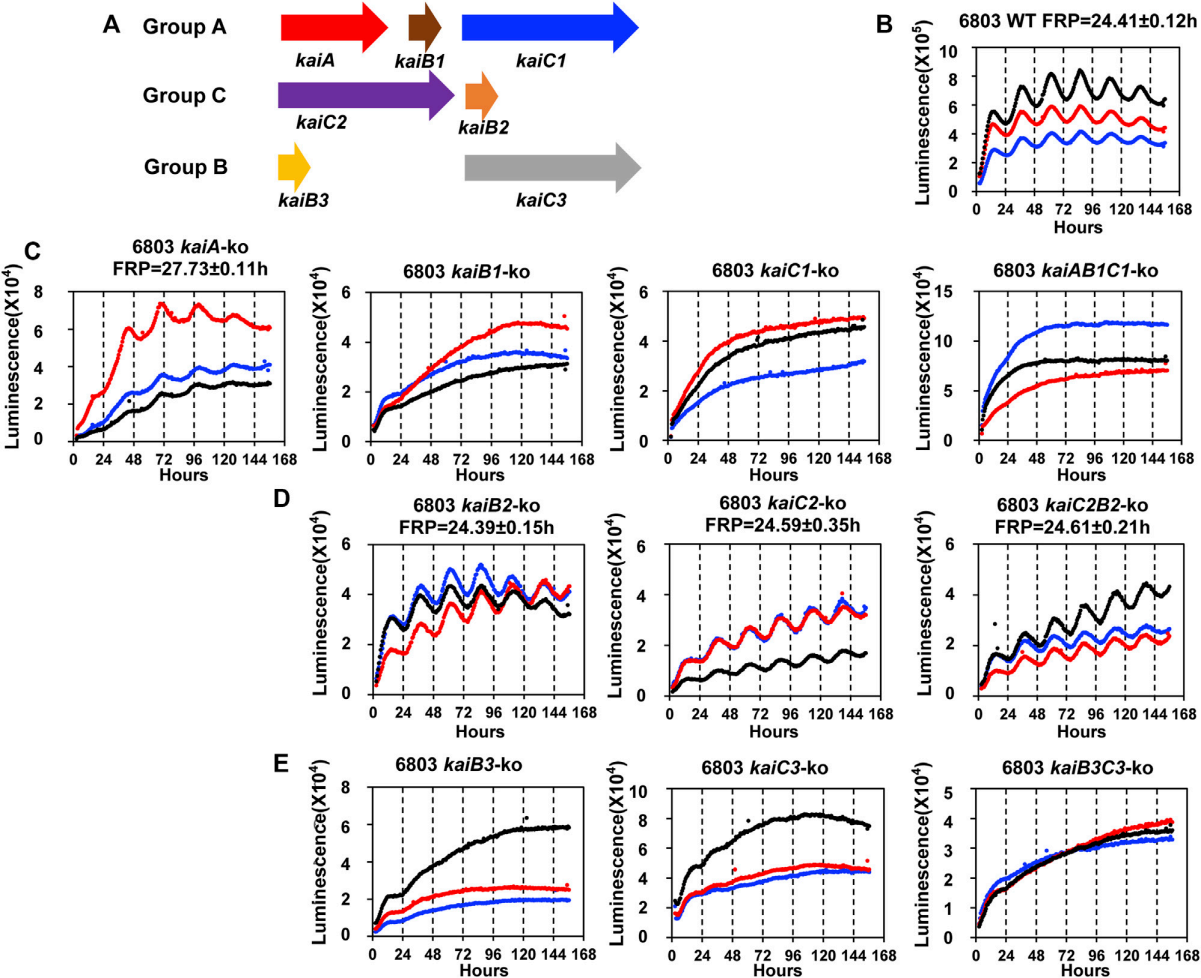
The rhythmicity of *Synechocystis sp*. PCC 6803 WT and *kai* null mutants on agar medium harboring the P_*psbA*_^*Ah*^::luxAB reporter in LL**. (A)**. Schematic diagram of the three sets of *kai* genes in *Synechocystis*. The *kaiA*, *kaiB1* and *kaiC1* genes are localized together on the chromosome as a clock gene cluster, and *kaiC1* belongs to GroupA that includes only *kaiC*s from cyanobacteria (Aoki & Onai, 2009). The *kaiC2* and *kaiB2* genes also localize together as a separate cluster, and *kaiC2* belongs to GroupC which includes *kaiC* homologs from Archaea and Proteobacteria. The *kaiB3* and *kaiC3* localize separately on the chromosome, and *kaiC3* belongs to GroupB which includes *kaiC* homologs from cyanobacteria, Proteobacteria and *Chloroflexi* (Aoki & Onai, 2009). **(B)**. The rhythmicity of WT *Synechocystis* colonies in LL as monitored with the Kondotron. The free-running period (FRP) of WT in this experiment was 24.41 ± .12 h (*n* = 3). Three representative colonies are shown. **(C)**. GroupA null mutants: expression patterns of *kaiA-ko*, *kaiB1-ko*, *kaiC1-ko*, *kaiAB1C1-ko* strains. All these null mutants were arhythmic except *kaiA-ko*, which expressed a reduced amplitude and long free-running period. **(D)**. GroupC null mutants: all three null mutants (*kaiB2-ko*, *kaiC2-ko* and *kaiC2B2-ko*) were rhythmic with the free-running periods indicated on the panels. **(E)**. GroupB null mutants: All three null mutants (*kaiB3-ko*, *kaiC3-ko* and *kaiB3C3-ko*) were arhythmic. Three representative traces are shown in each panel. FRP = free-running period.

We therefore applied the P_*psbA*_^*Ah*^::luxAB reporter to address a previously unresolved question, namely what is the role of the various *kaiC* genes in circadian rhythmicity in *Synechocystis*? In *S. elongatus*, there is a single *kaiABC* clock gene cluster which encodes the central core clock proteins mediating the clockwork (Ishiura et al., 1998; Johnson et al., 2017), but in *Synechocystis*, there are three *kaiC* homologs organized with and without *kaiA* and *kaiB* homologs (Figure 2A) (Aoki & Onai, 2009). When the presence of *kaiC* homologs among bacterial species is assessed globally, it is not unusual to find species with two or more *kaiC* homologs (Aoki & Onai, 2009; Schmelling et al., 2021). Since *S. elongatus* is able to elaborate a precise clockwork with only one *kaiC* homolog, what is the function of multiple *kaiC* genes in those species that harbor more than one copy? *Synechocystis* is an excellent test case, so we undertook to make null strains in which each *kai* cluster (and each *kai* gene individually) was knocked out to determine if its presence was necessary to enable robust rhythmicity of gene expression as reported by P_*psbA*_^*Ah*^::luxAB (see Supplementary Figure S2 for the genotyping of the knock-out strains). Figure 2 illustrates the results of the gene knockouts upon the luminescence rhythm, where Figure 2B shows the data for three representative wild-type (WT) colonies. As was found for the case of the *kaiABC* cluster in *S. elongatus* (Ishiura et al., 1998), knocking out the *kaiAB1C1* cluster or the *kaiB1* or *kaiC1* genes individually led to immediate arhythmicity (Figure 2C). Of the *kai* homologs in *Synechocystis*, the *kaiB1* and *kaiC1* genes are the most similar to *kaiB* and *kaiC* from *S. elongatus* (GroupA *kai* homologs (Aoki & Onai, 2009).

Surprisingly–and unlike the case for *S. elongatus*–knocking out the *kaiA* gene did not cause total arhythmicity (although the amplitude of the rhythm was reduced and the free-running period (FRP) was lengthened). In non-cyanobacterial species, there is precedence for the hypothesis that KaiB and KaiC can generate a daily timekeeping process in the absence of KaiA (Ma et al., 2016). Indeed, even in *S. elongatus*, the deletion of KaiA has been reported to allow the expression of a damped circadian oscillation under some conditions (Kawamoto et al., 2020). Another interpretation of the data in Figure 2C based on the known mechanism for the cyanobacterial clockwork of *S. elongatus* is that there might be another protein in *Synechocystis* that exhibits KaiA-like functionality. Based on the operon structure of *kaiABC* in *S. elongatus* (Ishiura et al., 1998), it is possible that the knockout of *kaiA* has disrupted the promoter region of *kaiB1C1*, but this is not likely because 1) the *kaiA*-ko strain is still rhythmic whereas the *kaiB1*-ko, *kaiC1*-ko, and *kaiAB1C1*-ko strains are all arhythmic, and 2) computational prediction of the *kaiB1C1* promoter is most likely to occur in the 126bp intergenic region between *kaiA* and *kaiB1* (Supplementary Figure S3).

Another surprise was that knocking out the GroupC *kaiCB* cluster had essentially no effect on the circadian rhythm (Figure 2D), despite the suggestion of circadian-like functions mediated by GroupC *kaiB* and *kaiC* in the purple bacterium *Rhodopseudomonas* (Ma et al., 2016). The final unexpected result was that knocking out *kaiB3* and/or *kaiC3* also abolished (or at least dramatically reduced the amplitude of) the rhythm even though *kaiC2* is more similar to *kaiC1* (and *kaiC* from *S. elongatus*) than is *kaiC3* (Figure 2E). Therefore, in terms of the circadian gene expression rhythm reported by P_*psbA*_^*Ah*^::luxAB, *kaiAB1C1* and *kaiB3*/*kaiC3* appear to be important for rhythmicity, whereas *kaiC2B2* is not. The adaptive fitness value of each of these *kai* clusters was assessed by competition experiments described below.

**FIGURE 3:**
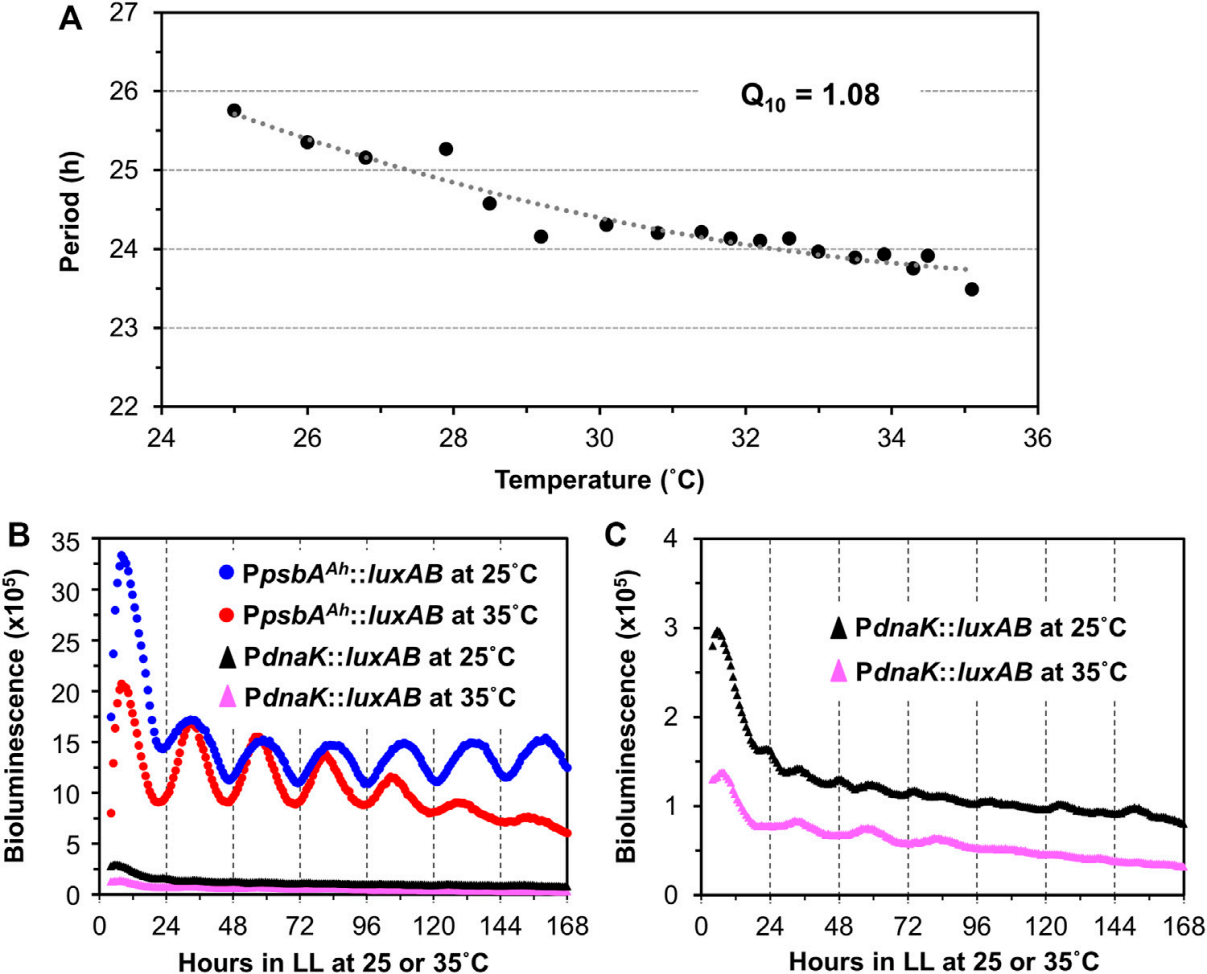
Temperature compensation of the P_*psbA*_^*Ah*^::luxAB-driving luminescence rhythms in *Synechocystis* PCC 6803. **(A)**. Free-running periods (FRPs) and Q_10_ value of the P_*psbA*_^*Ah*^::luxAB reporter measured from WT *Synechocystis* colonies on Petri dishes containing agar medium at various temperatures in LL by the Taylortron. Q_10_ was calculated as described in the Materials and Methods. **(B)**. Comparison of representative luminescence rhythms of the P_*psbA*_^*Ah*^::luxAB and P_*dnaK*_ *::luxAB* reporters monitored in constant light at 25°C and 35°C with the Taylortron. **(C).** The same luminescence rhythms for the P_*dnaK*_ *::luxAB* reporter shown in panel B but on an expanded scale.

**FIGURE 4:**
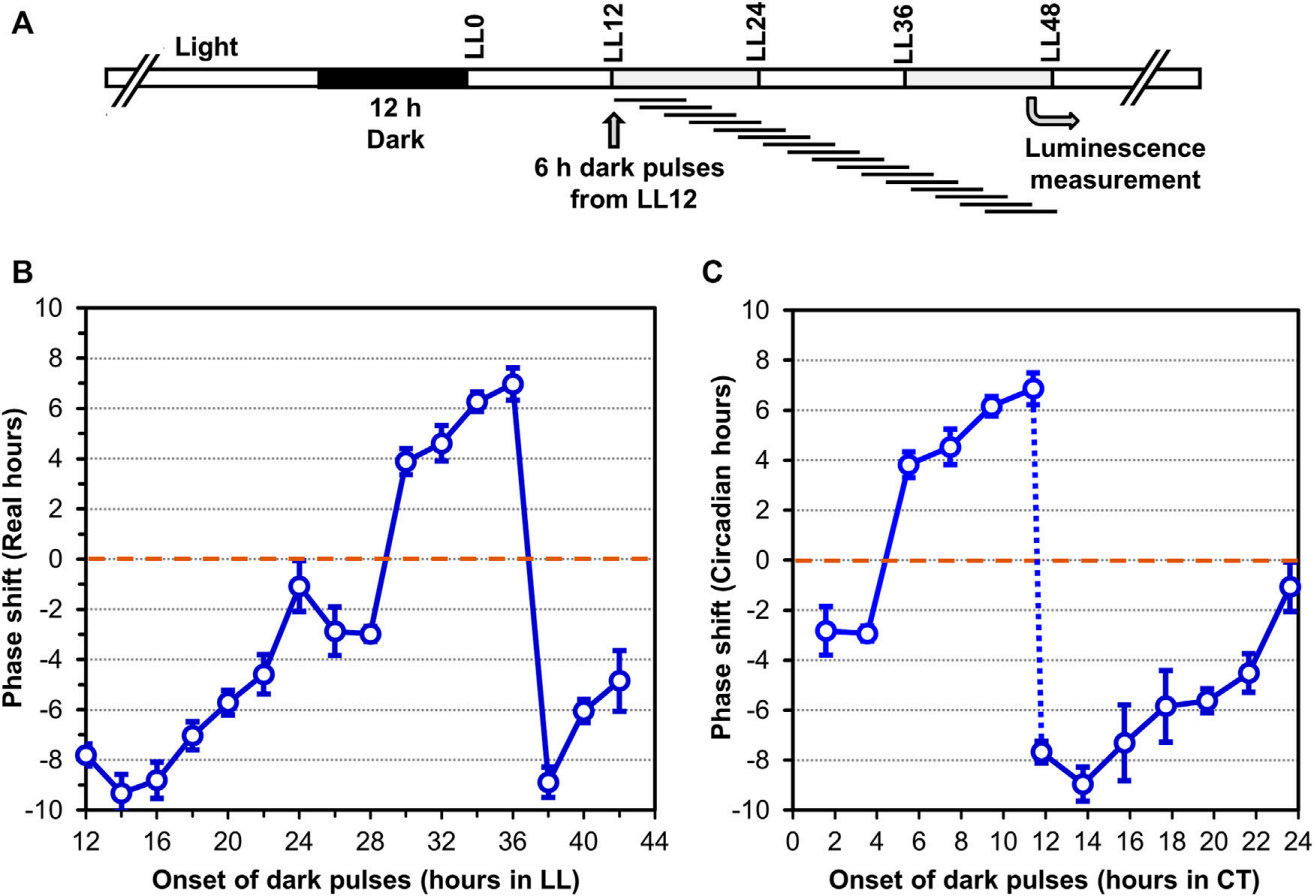
Phase Response Curve (PRC) to 6-h dark pulses of the luminescence rhythms reported by P_*psbA*_^*Ah*^::luxAB in *Synechocystis* PCC 6803. **(A)**. Protocol of the experiment where 6-h dark pulses were applied at different time points in constant light (LL) beginning 12 h after the synchronizing 12-h dark exposure. After all dark pulses were completed (at LL48), the cultures were placed in the Kondotron turntable luminescence monitoring apparatus and the bioluminescence rhythms were monitored for the next 7 days. **(B,C)**. Phase shifts caused by the 6-h dark pulses are plotted as a function of progressive time in constant light (Panel B) or as Circadian Time (=CT; Panel **(C)**. Advance *versus* delay phase shifts are determined on the basis of whether the shifted peak is 12 h or less earlier than the control (= Advance phase shifts, plotted as + values), *versus* 12 h or less later than the control (= Delay phase shifts, plotted as–values). Data are averages and standard deviations from six replicates.

In addition to persisting free-running rhythmicity under constant conditions, the other two defining properties of circadian rhythms are “temperature compensation” of the FRP and entrainment by environmental cycles (usually light/dark signals; (Pittendrigh, 1960; Johnson et al., 2017), both of which were established for the *S. elongatus* rhythms (Kondo et al., 1993). Consistent with a previous report (Aoki et al., 1995), temperature compensation of the FRP for *Synechocystis* is clearly demonstrated by the near-independence of FRP over a temperature gradient of 25°C–35°C (Figure 3A). Over this temperature range, the FRP alters by only ∼2 h, leading to a Q_10_ value of 1.08 (1.00 would be perfectly temperature independent), which is in the typical range for circadian clocks (Sweeney & Hastings, 1960). Panels B & C of Figure 3 underscore the value of using the P_*psbA*_^*Ah*^::luxAB reporter at 25°C and 35°C, where the amplitude of the P_*psbA*_^*Ah*^::luxAB rhythms are robust, whereas the amplitudes reported by P_*dnaK*_ *::luxAB* are poor (and even somewhat bimodal) at the lower and higher temperatures. Entrainment to environmental cycles is another defining property, which can often be assessed by measuring the phase-dependent resetting by light or dark pulses that can be graphed as a Phase Response Curve (PRC) (Johnson, 1990). Because we are measuring *Synechocystis* rhythms in LL, we used dark pulses to elicit phase resetting. As expected, phase resetting to 6-h dark pulses exhibit a PRC with significant phase advances in the subjective day (CT 0-12) and phase delays in the subjective night (CT 12-24, see Figure 4), which is a commonly observed pattern for dark pulse PRCs from microorganisms to mammals (Boulos & Rusak, 1982; Johnson et al., 1989), and is appropriately in antiphase with the previously reported light-pulse PRC for *Synechocystis* (Aoki et al., 1997). Thus, all three defining properties of circadian pacemakers are satisfied by the rhythms reported by P_*psbA*_^*Ah*^::luxAB in *Synechocystis*.

**FIGURE 5:**
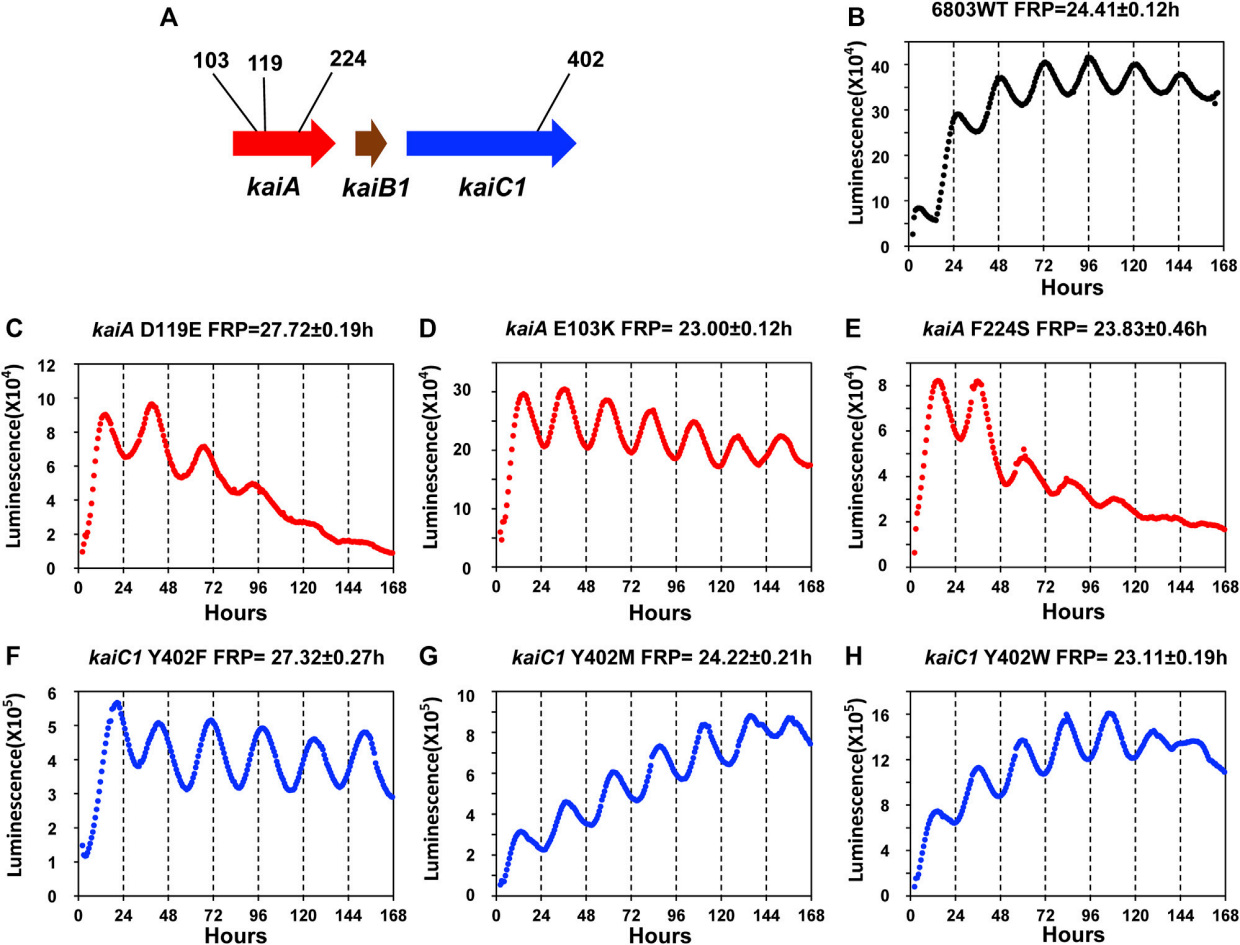
Luminescence phenotypes of period mutants in *Synechocystis* generated by point mutations that were informed by mutations identified in *S. elongatus*. **(A)**. Schematic diagram of the *kaiA* and *kaiC1* loci identifying the positions of the point mutations. Panels **(B–H)**: rhythmicity of representative colonies of the indicated strain/mutant of *Synechocystis* in LL monitored by the Kondotron. **(B).** WT *Synechocystis* (FRP = 24.41 ± .12 h). **(C)**. *kaiA* ^D119E^ (FRP = 27.72 ± .19 h). **(D)**. *kaiA* ^E103K^ (FRP = 23.00 ± .12 h). **(E)**. *kaiA* ^F224S^ (FRP = 23.83 ± .46 h). **(F)**. *kaiC* ^Y402F^ (FRP = 27.32 ± .27 h). **(G)**. *kaiC* ^Y402M^ (FRP = 24.22 ± .21 h). **(H)**. *kaiC* ^Y402W^ (FRP = 23.11 ± .19 h). All FRP data are means and S.D. from three replicates.

In *S. elongatus*, the P_*psbAI*_::*luxAB* reporter enabled a mutant screening that identified a stable of FRP mutants that ultimately led to the identification of the *kaiABC* gene cluster (Kondo et al., 1994; Ishiura et al., 1998). The brightness and robustness of the P_*psbA*_^*Ah*^::luxAB reporter should make possible such a mutant screen in *Synechocystis*. However, given 1) the large quantity and variety of FRP mutants in *S. elongatus* for which the altered sequence is known and 2) the excellent sequence conservation between *kaiABC* in *S. elongatus* with that of *kaiAB1C1* in *Synechocystis*, it might be possible to create the first FRP mutants in *Synechocystis* on the basis of the known mutations in *S. elongatus*. We set out to test that prediction by directed mutagenesis (Figure 5A), and indeed this prediction was upheld. Mutations of *Synechocystis’ kaiA* gene based on the work of Nishimura and coworkers with *S. elongatus* (Nishimura et al., 2002) led to the generation of three mutant strains with FRPs from ∼28 h to ∼23 h (*kaiA* ^D119E^, *kaiA* ^E103K^, *kaiA* ^F224S^, Figures 5C–E). Moreover, based on the fantastic range of FRPs generated by mutations to a single residue (Tyrosine^402^) of *S. elongatus’ kaiC* that was reported from the Kondo lab (Ito-Miwa et al., 2020), we revealed three mutations in Y402 of *Synechocystis*’ *kaiC1* that ranged from ∼23 h to ∼27 h (*kaiC1* ^*Y402F*^ *, kaiC1* ^*Y402M*^ *, kaiC1* ^*Y402W*^, Figures 5F–H). The similarity of the effect of these mutations in *Synechocystis* with those in *S. elongatus* confirms that the *kaiAB1C1* gene is the master clock locus in *Synechocystis*. However, the results in Figure 2E implies an essential role for *kaiC3* that could create a *kaiC1-kaiC3* redundancy that might mask mutations in *kaiC1*, but at least so far, this has not been observed. It would probably be worthwhile to test the effects of the mutations shown in Figure 5 in the *kaiB3/kaiC3* background, as well as testing point mutations in *kaiC3*.

**FIGURE 6:**
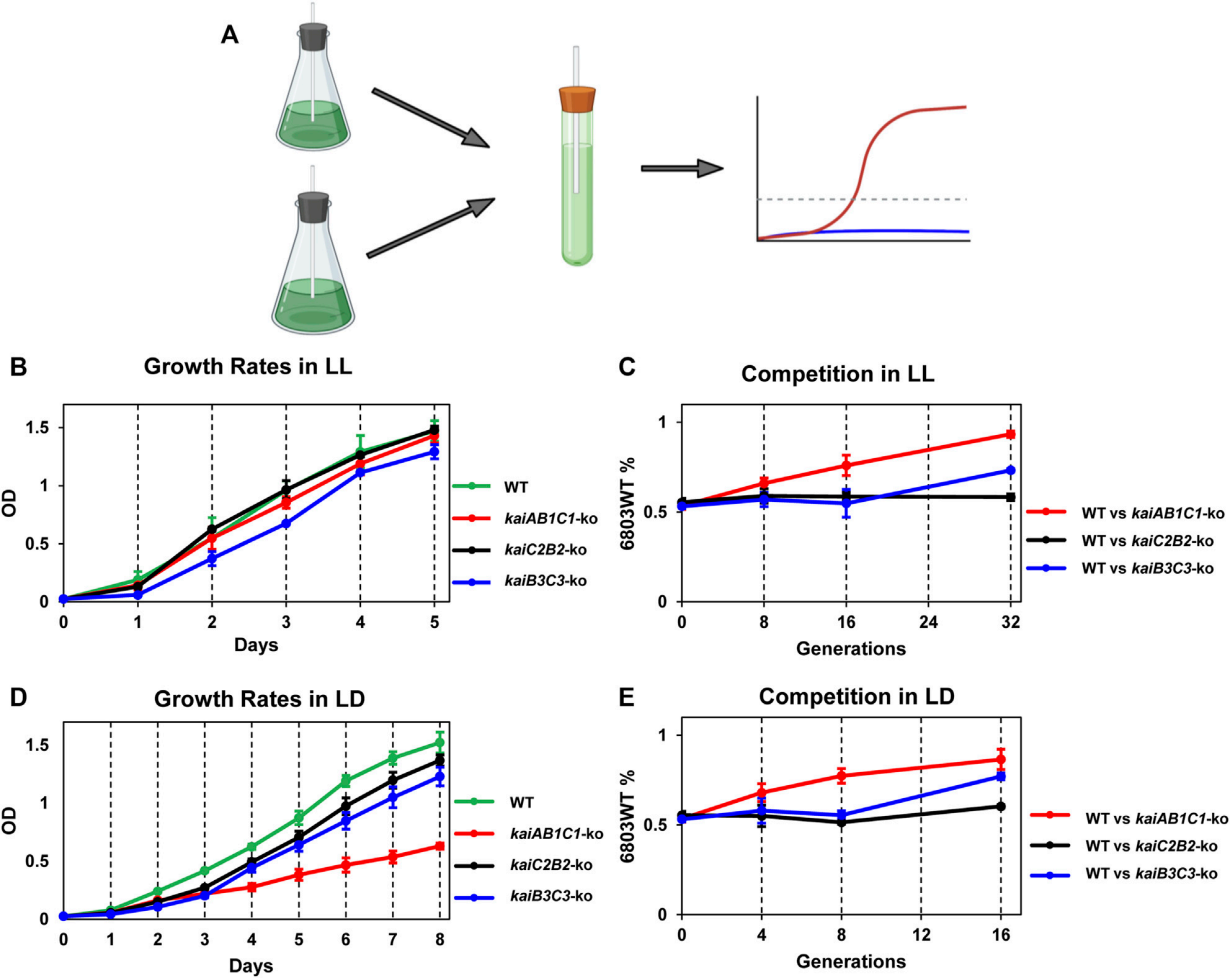
Adaptive fitness of the various *kai* gene clusters assessed by growth rates of monocultures and competition assays. The Synechocystis strains that were tested include wild-type (WT) and knockouts of the three different *kai* gene clusters (*kaiAB1C1-ko, kaiC2B2-ko* and *kaiB3/kaiC3-ko*). **(A)**. Mixed-strain cultures were grown in competition and changes in population structure quantified by QPCR of strain-specific sequences. **(B)**. Growth curves of the four strains in LL as assessed by light scattering of the cell culture (measured at OD_730_) as a function of time. **(C)**. Competition of WT against the *kaiAB1C1-ko, kaiC2B2-ko* or *kaiB3C3-ko* strains over generations/time in LL. The ordinate plots the percentage of the WT strain over time under competition. **(D)**. Growth curves of the four strains in LD 12:12 as assessed by light scattering of the cell culture (measured at OD_730_) as a function of time. **(E)**. Competition of WT against the *kaiAB1C1-ko, kaiC2B2-ko* or *kaiB3C3-ko* strains over generations/time in LD 12:12. The ordinate plots the percentage of the WT strain over time under competition. Data are plotted as means ± S.D. from three biological replicates.

To test if the various *kai* clusters in *Synechocystis* influence the fitness of the cells in LL or LD, we performed mixed-strain competitions modeled upon those we pioneered for assessing the adaptive significance of circadian periodicity in *S. elongatus* (Figure 6; (Woelfle et al., 2004). First, we assessed the single-strain growth rates of the WT, *kaiAB1C1*-ko, *kaiC2B2*-ko, and *kaiB3/kaiC3*-ko strains individually in LL, which is the non-selective environmental condition (Figure 6B). In LL, only the *kaiB3/kaiC3*-ko strain appears to grow significantly slower than the other three strains. In mixed cultures, WT outcompetes *kaiAB1C1*-ko even though there was not a significant difference in their growth rates in pure cultures (Figure 6C). The rhythm of the *kaiB3/kaiC3*-ko strain was severely disrupted (Figure 2E), and its growth was slower which apparently leads to out-competition by WT in mixed cultures (Figures 6B,C). In the clock-selective condition of LD 12:12, the *kaiAB1C1*-ko has a significantly lower growth rate. And both of the strains whose rhythms are disrupted (Figures 2C,E), namely *kaiAB1C1*-ko and *kaiB3/kaiC3*-ko, are out-competed by WT in LD (Figure 6E). Interestingly, rhythms in *kaiC2B2*-ko seem to be largely unaffected (Figure 2D), and the growth rate and competitive ability of this strain appears to be relatively equivalent to WT in both LL and LD (Figure 6).

## Discussion

Clearly, circadian rhythmicity in *Synechocystis* is robust (Figures 1–5) and adaptive (Figure 2; Figure 6). In hindsight, it was fortuitous that the first cyanobacterial species to be analyzed genetically for circadian rhythmicity was *S. elongatus* rather than *Synechocystis* (Kondo et al., 1993; Kondo et al., 1994; Ishiura et al., 1998). First, *S. elongatus* has a single *kaiABC* cluster (Ishiura et al., 1998), whereas *Synechocystis* and many other cyanobacterial species have multiple copies/clusters of *kai* genes that might have complicated the initial analysis of the genetic basis of the circadian clockwork (Aoki & Onai, 2009; Schmelling et al., 2021). Second, the first luciferase reporter created in *S. elongatus* for other experimental goals, the *P*_*psbAI*_::*luxAB* reporter, turned out to exhibit excellent high-amplitude rhythms (Kondo et al., 1993) that were optimal for enlistment to the high-throughput screen for clock mutations (Kondo et al., 1994) that ultimately led to the discovery of the *kaiABC* clock gene cluster (Ishiura et al., 1998). However, *P*_*psbAI*_::*luxAB* is not uniquely high amplitude in *S. elongatus*–in fact, in contrast to *Synechocystis,* most promoters from *S. elongatus* exhibit high amplitude rhythms (Liu et al., 1995). Therefore, serendipitously *S. elongatus* turned out to be an optimal species to begin circadian analyses in cyanobacteria.

At first, it might seem surprising that a promoter from a very different organism exhibits a better rhythm than the endogenous promoters that have been tested in *Synechocystis*. The example of *S. elongatus,* however, again exemplifies this principle; the *conII* promoter from *E. coli* expresses a beautiful rhythm in *S. elongatus* when coupled to a *luxAB* reporter (Xu, et al., 2013b). In *S. elongatus*, this result has been interpreted to mean that the entire chromosome is undergoing circadian cycles of supercoiling/compaction that confer circadian rhythmicity on all promoters, even heterologous promoters (Woelfle et al., 2007; Xu et al., 2013b). The P*psbA* ^*Ah*^ promoter used for driving the expression of *luxAB* in this study is among the strongest constitutive promoters characterized in cyanobacteria so far. It is a hybrid of the core promoter region of a chloroplast *psbA* gene (from *Amaranthus hybridus*) and an artificial RBS generated using the RBS Calculator (Salis et al., 2009; Xiong et al., 2015). This promoter-RBS hybrid, sometimes with subtle modifications, exhibited superior performance in expressing a variety of genes, such as the ethylene-forming enzyme (*Efe*), catalase (*KatE*), and two synthetic hydrogenase operons, rendering up to 12% of the total soluble protein as the target enzyme in *Synechocystis* (Xiong et al., 2015; Wang et al., 2018a). In addition, the same promoter was also able to drive strong expression of *Efe* and a limonene synthase in *S. elongatus*, and this capability was used to eliminate pathway bottlenecks (Wang X. et al., 2016; Wang et al., 2021a). Investigating the behaviors of biotechnology-relevant strong promoters, such as the herein studied P*psbA* ^*Ah*^ promoter, in response to the circadian rhythm regulation in *Synechocystis* could provide important information for guiding the synthetic biology and metabolic engineering efforts in *Synechocystis* in the future.

As we previously found for *S. elongatus* (Woelfle et al., 2004), the rhythmic WT strain of *Synechocystis* was able to outcompete arhythmic strains in LD (Figure 6). This experimental result clearly indicates the adaptiveness of appropriately aligning an internal oscillator with the external environmental cycle, and therefore that “desynchrony” of these two rhythms hampers competitiveness. Interestingly, arhythmic strains are able to compete successfully with WT in the non-selective LL conditions for *S. elongatus* (Woelfle et al., 2004), whereas WT *Synechocystis* is also dominant in LL (Figure 6), implying a fitness advantage for temporal organization in *Synechocystis* even when the environment is not rhythmic. On the basis of these knockout studies, the *kaiC2B2* cluster appears to not contribute to rhythmicity nor to fitness (Figure 2; Figure 6). This result is unexpected, given that GroupC *kai* genes do contribute to fitness in rhythmic environments in *Rhodopseudomonas* (Ma et al., 2016). Interestingly, we found that knocking out *kaiB3* and/or *kaiC3* drastically attenuated the circadian rhythm in *Synechocystis*, a phenotype that was unexpected when compared to the findings from a previous report in which *ΔkaiC3* mutant did not exhibit any apparent growth defect relative to the WT *Synechocystis* strain under photoautotrophic and photomixotrophic conditions during spot assays (Dörrich et al., 2014). In the current study, we not only found that growth of the *ΔkaiB3C3* mutant was significantly impaired (Figure 6), but also that the circadian rhythmicity of *Synechocystis* cells was eliminated when either *kaiB3* or *kaiC3* was knocked out (Figure 2). Our results hence provided sufficient evidence that Group B *kai* genes, *i.e*., *kaiB3* and *kaiC3,* are essential to the circadian clock of *Synechocystis*.

In this study, we developed a new methodology for measuring the proportions of two strains in mixed cultures based on QPCR rather than on our previous method of differential antibiotic sensitivity and colony-forming unit (CFU) counting (Woelfle et al., 2004). An advantage of our new methodology is that both strains in a mixed culture can harbor the same antibiotic resistance, thereby avoiding the complication of whether having different antibiotic resistance genes in the two strains are having a fitness effect (we always undertake our competition assays in the total absence of antibiotics, but the resistance genes themselves might have an impact on intracellular metabolism). A potential disadvantage of the new QPCR method is that we are measuring chromosome copy numbers rather than cell numbers or CFUs. WT *Synechocystis* cells have an average number of chromosome copies of three to six per cell (Pope et al., 2020). However, if the number of chromosome copies has been changed in any of the *kai* knockout strains, then the absolute proportion of the cells might be biased. We consider this to be a minor liability because even if the two tested strains have different numbers of chromosomes/cell, the relative proportions and their change over time will still be valid. And for the competition assay, the most important factor to be measured is the change over time of the proportion of one strain to the other. Therefore, we believe that the advantages of the new method outweigh its potential disadvantages.

As stated above, *S. elongatus* has functioned as an excellent system that provided superb insights into circadian mechanisms and adaptiveness, but its “simple” *kaiABC* genetic background makes it impossible to study the orchestration of Kai proteins from different Kai Gene Groups. On the other hand, the more complicated *kai* genetics of *Synechocystis* may provide a more realistic view of redundant genetics in a prokaryote. Moreover, the mechanism of the GroupB and GroupC Kai proteins may be most easily addressed in *Synechocystis* and subsequently those insights can be applied to the analysis of other species of bacteria that harbor those other versions of the *kai* genes. In that sense, *Synechocystis* may be a more productive “springboard” towards understanding circadian rhythmicity in non-cyanobacterial species than has been *S. elongatus*. Therefore, *Synechocystis* may be the model system that enlarges the scope of our comprehensive understanding and appreciation of daily timekeeping mechanisms in bacteria.

## Materials and methods

### Strains and growth conditions

The cyanobacterium Synechocystis sp. PCC 6803 wild-type (WT) strain was the basis of these studies. The cyanobacteria were grown on modified BG11 medium on agar plates or in liquid (Rippka et al., 1979; Bustos & Golden, 1991) with appropriate antibiotics (Xue et al., 2014; Taton et al., 2020). Cells were grown at 30 °C under constant cool-white fluorescence light (LL, 40–50 µE/m^2^s), or given one or two 12-h light/12-h dark cycles (e.g., LD 12:12) before release to LL to synchronize the cells in the population. NEB^®^ 5-alpha Competent E. coli (New England Biolabs) were used for the plasmid construction and gene cloning with appropriate antibiotics. *E. coli* cells were grown on LB medium (Bertani, 1951, 2004), on agar plates or in liquid media at 37 °C.

### Generation of P_*psbA*_^*Ah*^::luxAB reporter

The core promoter sequence from the chloroplast *psbA* gene of the plant *Amaranthus hybridus* was coupled to an engineered ribosome binding site (Xiong et al., 2015; Wang et al., 2018a) and assembled with the coding sequences of the *Vibrio harveyi* luciferase structure gene *luxAB* to generate a 2.18 kb fragment of P_*psbA*_^*Ah*^::luxAB (Figure 1A). A 1.24 kb BbvC I/Hind III fragment on pJU158 (Xiong et al., 2015) was replaced with the P_*psbA*_^*Ah*^::luxAB fragment to produce the P_*psbA*_^*Ah*^-driving luminescence expression construct with a spectinomycin selection marker and the *sir0168*-flanking sequences (Xiong et al., 2015) of *Synechocystis* genome (Supplemental Figure S1). The transformed P_*psbA*_^*Ah*^::luxAB reporter was confirmed by luminescence expression and colony PCR with a 2.2 kb DNA product using a pair of primers 5-ATC​TCA​ATG​AAT​ATT​GGT​TGA-3 and 5-ACG​AGT​GGT​ATT​TGA​CGA​TGT​TG-3.

### Construction of null and period mutant strains

All the *kai* null mutants as well as the period mutants were constructed based on the P_*psbA*_^*Ah*^::luxAB reporter strain. The knockout plasmids were constructed in 5-alpha Competent *E. coli* first, and thereafter transformed into *Synechocystis sp*. PCC 6803 wild type strain harboring the P_*psbA*_^*Ah*^::luxAB reporter. Briefly, approximately 1 Kb upstream and 1 Kb downstream fragments of the target genes were amplified separately through PCR, then linked together with an antibiotic resistance gene. This larger DNA fragment was cloned into the linearized pMiniT™ 2.0 cloning vector (NEB^®^ PCR Cloning Kit, New England Biolabs) to prepare for the transformation into cyanobacteria. To obtain the *kaiA* knockout plasmid (*kaiA-ko-Em*), 1 kb upstream and 1 kb downstream regions of the *kaiA* orf were amplified through PCR, then linked by the Erythromycin (Em) resistance gene, and the resulting fragment was inserted into the linearized pMiniT™ 2.0 cloning vector (NEB^®^ PCR Cloning Kit, New England Biolabs). Equivalent methods were used to generate the *kaiB1-ko-Em* and *kaiB3-ko-Em* knockout plasmids with Erythromycin resistance. Similarly, we used a Kanamycin (Km) resistance knockout plasmid to generate knockouts of the *kaiC1*, *kaiAB1C1*, and *kaiB2* genes (*kaiC1-ko-Km, kaiAB1C1-ko-Km*, and *kaiB2-ko-Km*). We used a Chloramphenicol (Cm) resistant knockout plasmid to generate knockouts of the *kaiC2* and *kaiC2B2* genes (*kaiC2-ko-Cm* and *kaiC2B2-ko-Cm*). Finally, we used a Gentamycin (Gm) resistant knockout plasmid to generate a knockout of the *kaiC3* gene (*kaiC3-ko-Gm*). See Supplemental Figure S2 for the genotyping data that confirm these knockouts, Supplemental Figure S4 for the physical maps & DNA sequences of all the plasmids for generating null mutants, and Supplemental Table S1 for the primer sequences used.

The period mutants were constructed by site-directed mutagenesis of the *Synechocystis* genome to produce mutations based on circadian mutants discovered in *S. elongatus* (Ishiura et al., 1998; Nishimura et al., 2002; Ito-Miwa et al., 2020). These mutations were introduced into the *kaiAB1C1* locus. The plasmid contained the wild type *Promoter-kaiAB1C1* DNA sequence was first assembled into the NEB cloning vector (NEB^®^ PCR Cloning Kit, New England Biolabs), then point mutations were introduced into either the *kaiA* or the *kaiC1* gene with QuikChange II Site-Directed Mutagenesis Kit (Agilent). Briefly, a pair of primers containing the desired mutations were used to amplify the backbone plasmid through PCR with *PfuUltra* HF DNA polymerase. *DpnI* was used to digest the methylated template DNA, then the purified DNA was transformed to *E. coli* competent cells. The correct mutations were confirmed with Sanger Sequencing. The resulting plasmid which contained the chloramphenicol resistance cassette was then transformed into the *kaiAB1C1* null mutant strain to integrate the mutated version of *kaiAB1C1* into the endogenous site by homologous recombination, thereby reconstituting the *kaiAB1C1* locus with the mutation, and the resulting period mutants were resistant to both Spectinomycin and Chloramphenicol. See Supplementary Figure S4 for the physical maps & DNA sequences of all the plasmids for generating period mutants, and Supplementary Table S1for the primer sequences used.

Homologous recombination and natural transformation were used to obtain all the null and period mutants (Eaton-Rye, 2004; Clerico et al., 2007). Approximately 1 µg of plasmid DNA as well as 4–5 ml of a cyanobacterial liquid culture in log phase growth (OD_730_ ∼.4–.6) were mixed together in an Eppendorf tube. The mixture was left in the dark for 3–4 h, then spread on a BG11 agar plate without antibiotics and left in the light for 12–16 h. Top agar with appropriate antibiotics was poured on top of the plate. The recombinant colonies appeared in ∼10 days. Fully segregated mutants were obtained after streaking 4-5 times on the agar plates with antibiotics. The concentrations of the antibiotics supplemented in the media for the selection of *Synechocystis* mutants were: Spectinomycin 30 μg/ml, Kanamycin 10 μg/ml, Chloramphenicol 7.5 μg/ml, Erythromycin 10 μg/ml, and Gentamycin 5 µg/ml.

### Bioluminescence monitoring

Synechocystis luminescence reporter strains were grown in modified BG11 medium on agar plates that was supplemented with appropriate antibiotics at 30°C under continuous cool-white illumination (LL; 40–50 µE/m^2^s). Circadian rhythms of luminescence from *Synechocystis* are generally more robust from colonies on agar plates as compared with liquid cultures. Fresh agar cultures grown in LL for two or 3 days were tooth-picked onto freshly made agar plates and after further growth in LL for one or days, a 12 h dark exposure was given to synchronize the clocks in the populations. Bioluminescence measurements of the *Synechocystis* colonies on agar media in LL were carried out using automated luminescence measuring systems (Kondo et al., 1993; Kondo et al., 1994; Johnson & Xu, 2009). A custom computer controlled turntable/CCD camera apparatus called the “Kondotron” was used to monitor the *in vivo* luminescence of single colonies on agar plates (Kondo et al., 1994). For measurement of luminescence rhythms over a broad range of temperatures, a custom apparatus called the “Taylortron” was used (Johnson & Xu, 2009). The Taylortron comprises a hollow metal bar with 30 positions, each of which can hold a 20-ml scintillation vial with a cyanobacterial culture on liquid or agar media. A temperature-controlled heater blows hot air into one end of the metal bar, thereby generating a temperature gradient along the bar that can be adjusted by appropriately altering the heater’s setting relative to the setpoint of the temperature-controlled room that houses the Taylortron. In the Taylortron, a computer-controlled cart with a photomultiplier tube moves from position to position and automatically monitors the intensity of the luminescence emission from the 20 ml vials containing 3–5 ml of liquid or agar medium. For the temperature gradient assay in this study, 12 colonies from fresh agar cultures of the WT reporter strain were tooth-picked onto the surface of the agar inside each vial, and then an open Eppendorf tube containing 0.1 ml oil with 1% decanal was also put inside the vial. After a 12 h dark synchronization, the luminescence rhythms were measured at a gradient of temperatures in the Taylortron in LL at an intensity of ∼50 µE/m^2^s. The Taylortron was used to monitor the rhythms in the experiments of Figure 3, whereas the turntable “Kondotron” (Johnson & Xu, 2009) was used to monitor the rhythms in the experiments of Figures 1–5.

### Q_10_ measurement and calculations

The different periods of luminescence rhythms of the wild type *Synechocystis* sp. PCC 6803 over the temperature ranges from 25°C to 35°C were analyzed with ChronoAnalysis II, version 10.1 (courtesy of T. Roenneberg) (Xu et al., 2013a), and the temperature coefficient Q_10_ for the evaluation of Temperature Compensation was estimated by fitting Eq 1 to a set of the data points (*i.e.,* circadian periods that were experimentally obtained at various temperatures).

$$Q_{10}={\left(\frac{\tau_{30}}{\tau_{t}}\right)}^{\left(\frac{10}{t-30}\right)}$$ (1)

where the dependent variable $\tau_{t}$ is period length at t°C , the fitting parameter $\tau_{30}$ is the period at 30 C, and the fitting parameter $Q_{10}$ is the temperature coefficient.

### Dark-pulse phase response curve

Colonies of wild type *Synechocystis* on agar plates were given a 12-h light/12-h dark cycle (LD 12:12) to synchronize all the cells, then the colonies were released into LL. Starting from 12 h in the light (LL12), the agar plates were sequentially given a 6-h dark treatment followed by the next plate 2 h later (from LL12 to LL42). A control plate remained in LL without any dark-pulse treatment. Phase shifts that resulted from these dark pulses were calculated with ChronoAnalysis II, version 10.1 (courtesy of Dr. T. Roenneberg); the values at each timepoint were calculated from eight replicate colonies (Xu et al., 2000).

### Intra-species competition

Single-strain monocultures of *Synechocystis* WT, *kaiAB1C1-ko, kaiC2B2-ko* and *kaiB3C3-ko* were grown under 30°C and constant cool-white fluorescence light (LL, 40–50 µE/m^2^s) to OD_730_ .4-.6. Then WT was mixed with *kaiAB1C1-ko, kaiC2B2-ko* and *kaiB3C3-ko* respectively in 30 ml liquid BG11 to an OD_730_ ratio of 1:1. The starting OD_730_ of the mixed-strain culture was .2. The competition of the mixed-strain cultures (3 biological replicates for each combination) was conducted in either LL or LD 12:12 with the cultures bubbled with air to provide aeration (especially CO_2_). Cultures were diluted every 8 d, and samples were collected for quantification on Days 0, 4, 8 and 16. Quantitative QPCR was used to quantify the copy numbers of the integrated antibiotic resistance genes as a proxy for chromosome number in the WT strain *versus* the other strain in each competition combination.

### Genome DNA extraction and absolute quantitative QPCR calculations

For the measurement of the relative percentages of *Synechocystis* chromosome copy numbers of WT and *kai* mutants in the competition experiments, absolute quantitative QPCR was applied. A pair of primers targeting the *Spectinomycin* resistant gene was used to quantify both strains in each combination (P_*psbA*_^*Ah*^ *::luxAB* was inserted into the genome with the Spectinomycin resistance gene cartridge)*,* whereas primers targeting the Kanamycin, Chloramphenicol, and Erythromycin resistance genes were used to quantify the *kaiAB1C1, kaiC2B2* and *kaiB3C3* null mutants respectively. We first amplified the DNA fragments targeting the regions on Spectinomycin, Kanamycin, Chloramphenicol and Erythromycin resistance genes, purified them with the Monarch^®^ DNA Gel Extraction Kit (New England Biolabs) and measured their concentrations. Then we did serial dilutions of each specific DNA fragment to known concentrations (e.g., to 10–13 mol/µL, 10–14 mol/µL, 10–15 mol/µL, 10–16 mol/µL, 10–17 mol/µL and 10–18 mol/µL). Henceforth, those DNA fragments were used as the standard samples to calculate DNA concentrations in the actual samples.

Ct (cycle time) values from the wells that contained the standard samples (with known concentrations) were used to draw standard curves to quantify the DNA concentrations and the Ct values, as shown inSupplementary Figure S5. From the wells that contained the actual samples (genome DNA extracted with Favorgen plant genomic DNA extraction Mini Kit FAPGK001-2), we used the Ct numbers obtained in QPCR to quantify the DNA concentrations of each strain separately, which were used to estimate chromosome copy number with the standard curves; in *Synechocystis*, the average number of chromosome copies is three to six per cell (Pope et al., 2020). By these comparisons, we calculated the relative percentages of each strain in the mixed cultures under competition.

## Data Availability

The original contributions presented in the study are included in the article/Supplementary Material, further inquiries can be directed to the corresponding author.
