# Supplementary material for: Synechocystis: A model system for expanding the study of cyanobacterial circadian rhythms
Source: Front Physiol. 2023 Jan 4;13:1085959. doi: 10.3389/fphys.2022.1085959 (PMC9846126; doi:10.3389/fphys.2022.1085959)
Supplement: Supplementary file 1 [file DataSheet1.PDF]

Supplemental Material for: *“Synechocystis*: A Model System for Expanding the Study of Cyanobacterial Circadian Rhythms”

Supplemental Table

Supplemental Table S1. Primer sequences for genotyping null mutants, period mutants, and antibiotic resistance genes.

Primers for null mutants and antibiotic resistances	Sequences
**kaiAup-1**	TTTAATGCCTGCTCGTAATCCAGCCGTTTAACTGTTTTAT
**kaiAup-2(Em)**	ttagtataattatagcacgcAGGGGATCAATAATCAGTCGAAGGTTAAT
**kaiAdown-1(Em)**	cgagcggtatcagctcactcaTAACCAAATGCTACGGTTTAATCATCAGCT
**kaiAdown-2**	TCAATTAAGGCCGACAGATCAAAGGTGCCCA
**kaiB1up-1**	TTTTGCTCCCGAACACGTTGCCCATAGACT
**kaiB1up-2(Em)**	attagtataattatagcacgcTTTACGGGTTTACTAGAACGAGGGGGAAAC
**kaiB1down-1(Em)**	cgagcggtatcagctcactcaAAAATCGGGGAACAGGGCGAGATAT
**kaiB1down-2**	CCGCCACACATTTCGTCCAAGGTTTGTACT
**kaiC1up-1**	TAGGAGTGTACTACAAACGTAAGCCCAGTCACTTTTA
**kaiC1up-2(Km)**	tgagtgcttgcggcagcgtgTAGCTAAATTTAGATTTTTAAAGAAGAAAAAAG
**kaiC1down-1(Km)**	tgctggagttcttcgcccaccGATTGAAAAAGGGTAAACTTTCCCAT
**kaiC1down-2**	TGATCCCACCGATGAAGGTTGGTCAGCTTTTAT
**kaiAB1C1up-1**	TTTAATGCCTGCTCGTAATCCAGCCGTTTAACTGTTTTAT
**kaiAB1C1up-2(Km)**	tgagtgcttgcggcagcgtgAGGGGATCAATAATCAGTCGAAGGTTAAT
**kaiAB1C1down-1(Km)**	tgctggagttcttcgcccaccGATTGAAAAAGGGTAAACTTTCCCAT
**kaiAB1C1down-2**	TGATCCCACCGATGAAGGTTGGTCAGCTTTTAT
**kaiB2up-1**	AGAGCGATGAAAACGTTTCAGTTTGCTCATGGAAAAC
**kaiB2up-2(Km)**	tgagtgcttgcggcagcgtgAATAGAAGACTCTTCAGTACAGAT
**kaiB2down-1(Km)**	gctggagttcttcgcccaccCATTTTAGGTTGTCGCAATGAAT
**kaiB2down-2**	AGACGCCAGCAGTCCTCCCCGGGAAGCAAATGAT
**kaiC2up-1**	TTCAAAATGCCTTGGTCACAGGCGGCGGCGGT
**kaiC2up-2(Cm)**	GACGATATGATCGACGGATCTGATTTTTACGGATTCAATTTACTTGCCAGCAT
**kaiC2down-1(Cm)**	GTATTATCCCGTGTGACGGATCTGTACTGAAGAGTCTTCTATTATGGAAAATT
**kaiC2down-2**	CCTAGTGCCGGTTGTTTAGATTCACAGTCACTCAAAAG
**kaiC2B2up-1**	TTCAAAATGCCTTGGTCACAGGCGGCGGCGGT
**kaiC2B2up-2(Cm)**	GACGATATGATCGACGGATCTGATTTTTACGGATTCAATTTACTTGCCAGCAT
**kaiC2B2down-1(Cm)**	GTATTATCCCGTGTGACGGATCTCATTTTAGGTTGTCGCAATGAATGTTC
**kaiC2B2down-2**	AGACGCCAGCAGTCCTCCCCGGGAAGCAAAT
**kaiB3up-1**	TCGGACCGTCGATTGTATCCCCTTTGGTGGAACGT
**B3up-2(Em)**	ttagtataattatagcacgcCGCTGATAACAAATAAAGCCAATGCT
**B3down-1(Em)**	cgagcggtatcagctcactcaCCGCTATCCCCTAGGCGGTGGGCCGAT

**B3down-2**	TTCGGGGTCTAATAATTCAATTAGACGATATCGTTCCTTTTC
**kaiC3up-1**	GGCTCGCTTGGGTTAGGCAGGGCATTATTGACGAAGT
**kaiC3up-2(Gm)**	TCGTAGATCATGGTCATAGCTCCTTTCACTGCCCCATACTCGTATTC
**kaiC3down-1(Gm)**	TGGCACTGGCCGTCGTTTTACTCAATTCTTCCCTTTGTAAACCTGGAGGC
**kaiC3down-2**	GGGGCACAATGGAAAAGGAAACCAGACTGTTAAT
**Em-1**	gcgtgctataattatactaattttataaggaggaaaaaatatgggc
**Em-2**	tgagtgagctgataccgctcgccgc
**Km-1**	cacgctgccgcaagcactcagggc
**Km-2**	ggtgggcgaagaactccagcatgagat
**Cm-1**	AGATCCGTCGATCATATCGTCAATTATTACCTCCA
**Cm-2**	AGATCCGTCACACGGGATAATACCGCGCCA
**Gm-1**	AGCTATGACCATGATCTACGAATTAGCTTGCAT
**Gm-2**	GTAAAACGACGGCCAGTGCCAAGCTTGCAT

**Primers for period mutants**	**Sequences**
kaiA-E103K-F	TCACAACGCTaAAATTCATCTGC
kaiA-E-103K-R	TAGACAATGGTGGGGTGT
kaiA-D119E-F	CCGTCGTAGAaCGGGCGATCG
kaiA-D119E-R	TGGGCAGTTCCGACCATTG
kaiA-F224S-F	TTAGATCAATaTGTTAATCAGGCTTTC
kaiA-F224S-R	CAGGTCATTAACTGTGCC
6803kaiC-402M-F	GGTAACGGGCatgGCCAAACAGGAGG
6803kaiC-402M-R	CCAATGACAAATTGACGG
6803kaiC-402F-F	GTAACGGGCTttGCCAAACAGG
6803kaiC-402F-R	CCCAATGACAAATTGACG
6803kaiC-402W-F	GTAACGGGCTggGCCAAACAGG
6803kaiC-402W-R	CCCAATGACAAATTGACG

Supplemental Figures (next page)

Supplemental Figures

A.

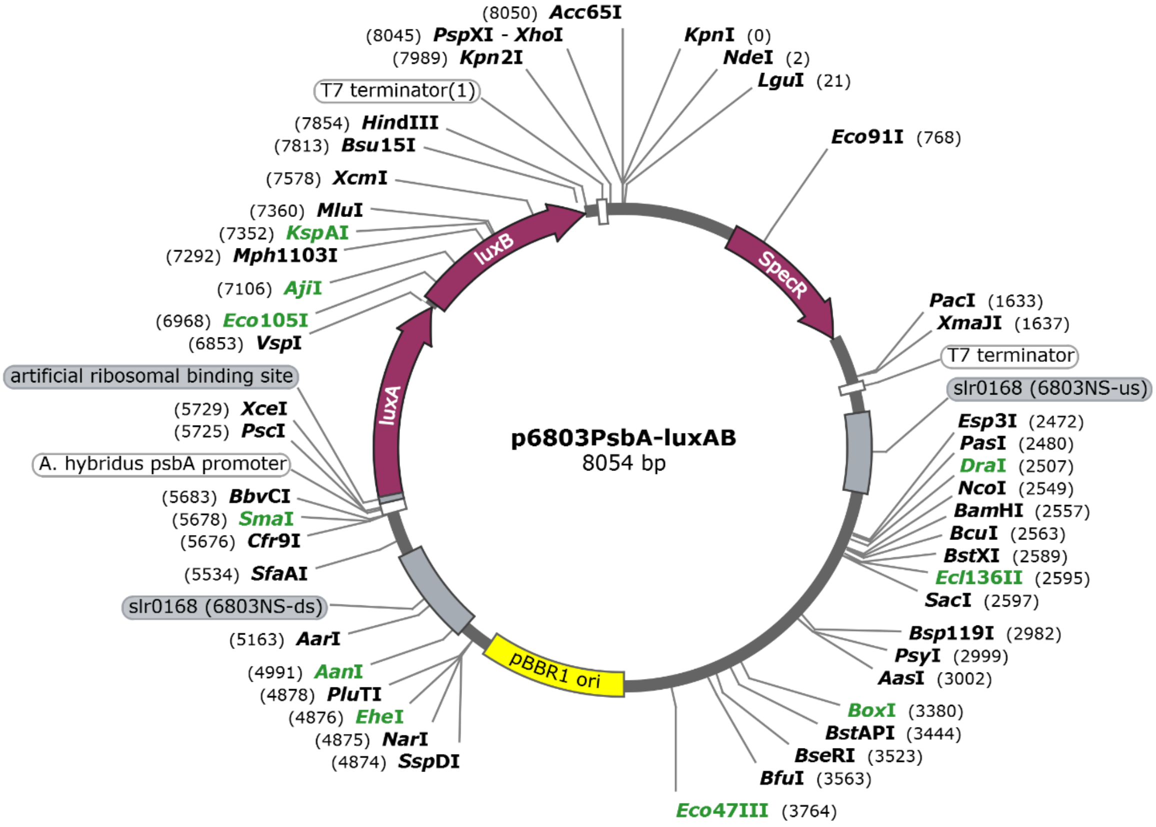

B.

catatgtctagagcggccgcttaacgaagagcatatatatacagaagctgggcgaacaaacgatgctcgccttccagaaaaccgaggatgcgaacc
acttcatccggggtcagcaccaccggcaagcgccgcgacggccgaggtcttccgatctcctgaagccagggcagatccgtgcacagcaccttgccgta
gaagaacagcaaggccgccaatgcctgacgatgcgtggagaccgaaaccttgcgctcgttcgccagccaggacagaaatgcctcgacttcgctgctg
cccaaggttgccgggtgacgcacaccgtggaaacggatgaaggcacgaacccagtggacataagcctgttcggttcgtaagctgtaatgcaagtagc
gtatgcgctcacgcaactggtccagaaccttgaccgaacgcagcggtggtaacggcgcagtggcggttttcatggcttgttatgactgtttttttggggt
acagtctatgcctcgggcatccaagcagcaagcgcgttacgccgtgggtcgatgtttgatgttatggagcagcaacgatgttacgcagcagggcagtc
gccctaaaacaaagttaaacatcatgagggaagcggtgatcgccgaagtatcgactcaactatcagaggtagttggcgtcatcgagcgccatctcga
accgacgttgctggccgtacatttgtacggctccgcagtggatggcggcctgaagccacacagtgatattgatttgctggttacggtgaccgtaaggct
tgatgaaacaacgcggcgagctttgatcaacgaccttttggaaacttcggcttcccctggagagagcgagattctccgcgctgtagaagtcaccattgt
tgtgcacgacgacatcattccgtggcgttatccagctaagcgcgaactgcaatttggagaatggcagcgcaatgacattcttgcaggtatcttcgagcc
agccacgatcgacattgatctggctatcttgctgacaaaagcaagagaacatagcgttgccttggtaggtccagcggcggaggaactctttgatccgg
ttcctgaacaggatctatttgaggcgctaaatgaaaccttaacgctatggaactcgccgcccgactgggctggcgatgagcgaaatgtagtgcttacgt
tgtcccgcatttggtacagcgcagtaaccggcaaaatcgcgccgaaggatgtcgctgccgactgggcaatggagcgcctgccggcccagtatcagcc

cgtcatacttgaagctagacaggcttatcttggacaagaagaagatcgcttggcctcgcgcgcagatcagttggaagaatttgtccactacgtgaaag
gcgagatcaccaaggtagtcggcaaataatgtctaacaattcgttcaagccgacgccgcttcgcggcgcggcttaactcaagcgttagatgcactaag
cacataattgctcacagccaaactatcaggtcaagtctgcttttattatttttaagcgtgcataataagccctacacaagccacgtaggggttgagacta
tcaatggtaatacccaccgcactgaccgggccgttgggtccccgtctactagcgcagcttaattaacctaggctgctgccaccgctgagcaataactag
cataaccccttggggcctctaaacgggtcttgaggggttttttgctgaaacctcaggcatttgagaagcacacggtcacactgcttccggtagtcaataa
accggtaaaccagcaatagacataagcggctatttaacgaccctgccctgaacgggacccaccatttccaaaggagtactgtccaacactgtgcctac
ggaaacgggataaatcgctccctccgtgcctggggttaaacgattaccaaaattgcccgtaattactaccgtttgccgttcgttgtattcactgttggga
atcaaagaggcgatcaccggggtgacaatttcccccgtgttaagcattacctgaaaatctgtgggattaagggtggtgggaaaaatgggccacgcaa
aaacaatgggaatgttatcgttttttttccagattaacgccataaacactctgcactgtgtctgtgctaatagccgaagtgtaggcccgttgggtggtggt
ggacaaatcccccaggttattccaggccacattgttgtcaaaggcaatctgttgggattccgcatcggtgccatccataccgggaattccgtatggcaa
tgaaagacggtgagctggtgatatgggatagtgttcacccttgttacaccgttttccatgagcaaactgaaacgttttcatcgctctggagtgaatacca
cgacgatttccggcagtttctacacatatattcgcaagatgtggcgtgttacggtgaaaacctggcctatttccctaaagggtttattgagaatatgtttt
tcgtctcagccaatccctgggtgagtttcaccagttttgatttaaacgtggccaatatggacaacttcttcgcccccgttttcaccatgggggggatccact
agttctagagcggccgccaccgcggtggagctccaattcgccctatagtgagtcgtattacgcgcgctcactggccgtcgttttacaacgtcgtgactgg
gaaaaccctggcgttacccaacttaatcgccttgcagcacatccccctttcgccagctggcgtaatagcgaagaggcccgcaccgatcgcccttcccaa
cagttgcgcagcctgaatggcgaatggaaattgtaagcgttaatattttgttaaaattcgcgttaaatttttgttaaatcagctcattttttaaccaatag
gccgactgcgatgagtggcagggcggggcgtaatttttttaaggcagttattggtgcccttaaacgcctggtgctacgcctgaataagtgataataagc
ggatgaatggcagaaattcgaaagcaaattcgacccggtcgtcggttcagggcagggtcgttaaatagccgcttatgtctattgctggtttaccggttt
attgactaccggaagcagtgtgaccgtgtgcttctcaaatgcctgaggccagtttgctcaggctctccccgtggaggtaataattgacgatatgatcatt
tattctgcctcccagagcctgataaaaacggtgaatccgttagcgaggtgccgccggcttccattcaggtcgaggtggcccggctccatgcaccgcgac
gcaacgcggggaggcagacaaggtatagggcggcgaggcggctacagccgatagtctggaacagcgcacttacgggttgctgcgcaacccaagtg
ctaccggcgcggcagcgtgacccgtgtcggcggctccaacggctcgccatcgtccagaaaacacggctcatcgggcatcggcaggcgctgctgcccg
cgccgttcccattcctccgtttcggtcaaggctggcaggtctggttccatgcccggaatgccgggctggctgggcggctcctcgccggggccggtcggta
gttgctgctcgcccggatacagggtcgggatgcggcgcaggtcgccatgccccaacagcgattcgtcctggtcgtcgtgatcaaccaccacggcggca
ctgaacaccgacaggcgcaactggtcgcggggctggccccacgccacgcggtcattgaccacgtaggccgacacggtgccggggccgttgagcttca
cgacggagatccagcgctcggccaccaagtccttgactgcgtattggaccgtccgcaaagaacgtccgatgagcttggaaagtgtcttctggctgacc
accacggcgttctggtggcccatctgcgccacgaggtgatgcagcagcattgccgccgtgggtttcctcgcaataagcccggcccacgcctcatgcgct
ttgcgttccgtttgcacccagtgaccgggcttgttcttggcttgaatgccgatttctctggactgcgtggccatgcttatctccatgcggtagggtgccgca
cggttgcggcaccatgcgcaatcagctgcaacttttcggcagcgcgacaacaattatgcgttgcgtaaaagtggcagtcaattacagattttctttaac
ctacgcaatgagctattgcggggggtgccgcaatgagctgttgcgtacccccctttttttaagttgttgatttttaagtctttcgcatttcgccctatatctag
ttctttggtgcccaaagaagggcacccctgcggggttcccccacgccttcggcgcggctccccctccggcaaaaagtggcccctccggggcttgttgat
cgactgcgcggccttcggccttgcccaaggtggcgctgccccttggaacccccgcactcgccgccgtgaggctcgggggggcaggcgggcgggcttcg
ccttcgactgcccccactcgcataggcttgggtcgttccaggcgcgtcaaggccaagccgctgcgcggtcgctgcgcgagccttgacccgccttccactt
ggtgtccaaccggcaagcgaagcgcgcaggccgcaggccggaggcttttccccagagaaaattaaaaaaattgatggggcaaggccgcaggccgc
gcagttggagccggtgggtatgtggtcgaaggctgggtagccggtgggcaatccctgtggtcaagctcgtgggcaggcgcagcctgtccatcagcttg
tccagcagggttgtccacgggccgagcgaagcgagccagccggtggccgctcgcggccatcgtccacatatccacgggctggcaagggagcgcagc
gaccgcgcagggcgaagcccggagagcaagcccgtagggcgccgcagccgccgtaggcggtcacgactttgcgaagcaaagtctagtgagtagca
acgttgttgccattgctgcagggaccattctctggatcattgccggggccaccgggattataaaccgccgaataatccccttcggagggcaaagcaac
cctcttaatttggcgaactgcggcttcgtcccctttgaggataatgtcgtaatagttgtcatgatcttcgatgtaagtcaggtcatagctgtcctgaacccc
ggccaaatccgccagccccaacacctgcaccagaccaaagccgggaatttcataatcaacaccagtttgggttaggataactgtccgtcccgtaatat
cttccgcttggagttgaaaataccgttcaaattctgtgggtagtaaactggcaatgccatcgggggaaaaaccggcgctggtgtaaattcgcaaacga
aattgggcttggtctccatataaatccccgccactgttattttgattggtggctaaccagaggggagccccttcccccaggtcactgaagcggtctaact
tagcggcgacaattttgggaccattgccgaacaccactgggcctactgggctattcccattatcatctacattgaagggatagcaagctaatttttatga
cggcgatcgccaaaaacaaagaaaattcagcaattaccgtgggtagcaaaaaatccccatctaaagttcagtaaatatagctagaacaaccaagca

ttttcggcaaagtactattcagatagaacgagaaatgagcttgttctatccgcccggggctgagggatctcaatgaatattggttgacacgggcgtata
agacatgttatactgttgaataacaagtttaccgttcccaaaaataaagaaggaggaacagcatgaaatttggaaacttccttctcacttatcagccac
ctgagctatctcagaccgaagtgatgaagcgattggttaatctgggcaaagcgtctgaaggttgtggcttcgacaccgtttggttgctagagcaccact
tcactgaatttgggttgttagggaatccttatgttgctgccgcacacctattaggtgcgacagaaacgctcaacgttggcactgcagctatcgtattgcc
gactgcccatccggttcgacaagcagaagacgtaaacctactggatcaaatgtcaaaaggacgattccgttttggtatttgtcgcggtttgtacgataa
agattttcgtgtctttggtacagacatggataacagccgagccttaatggactgttggtatgacttgatgaaagaaggcttcaatgaaggctatatcgc
ggcggataacgaacatattaagttcccgaaaatccaactgaatccatcggcttacacacaaggtggtgctcctgtttatgtcgtcgcggagtcagcatc
aacgacagaatgggctgcagagcgtggcctaccaatgattctaagctggatcatcaacactcacgagaagaaagcgcagcttgatctttacaacgaa
gtcgcgactgaacatggctacgatgtgactaagattgaccactgtttgtcttacatcacctccgtcgatcatgactcaaatagagccaaagatatttgcc
gcaacttcttgggccattggtacgactcatacgtgaatgccaccaagatttttgacgactctgaccaaacaaaaggttacgacttcaataaaggtcaat
ggcgtgattttgtgttgaaaggccacaaagacaccaatcgccgaattgattacagctacgaaatcaacccagtagggacgcctgaagagtgtatcgc
gattatccagcaagatattgatgcgacgggtattgacaatatttgttgtggttttgaagcaaacggttctgaagaagaaattatcgcatctatgaagcta
ttccagtctgatgtgatgccatatctcaaagaaaaacagtaattaatattttctaaaaggaaagagacatgaaatttggattattcttcctcaattttatg
aattcaaagcgttcttctgatcaagtcatcgaagaaatgttagataccgcacattacgtagatcagttgaagtttgacacgttggctgtttacgaaaacc
atttctcgaacaatggtgtggttggtgccccactaacagtggctggtttttacttggtatgacaaagaacgccaaagtggcttcgttgaatcacgtcatt
accacgcatcatccagtacgtgtggcggaagaagcgtgtctacttgaccaaatgagtgaaggccgttttgcctttggctttagtgattgtgaaaagagt
gcagatatgcgcttctttaatcgaccaacggattctcagtttcagttgttcagtgagtgtcacaagatcatcaatgatgcattcactactgggtactgcca
tccaaacaatgatttttatagttttcctaaaatctccgttaacccacacgcgttcactgaaggcggtcctgcgcaatttgtgaatgcgacgagcaaagaa
gtggttgaatgggcggctaagttagggcttccactcgtgtttagatgggacgactcaaacgctcaaagaaaagaatacgccggtttgtaccacgaagt
tgctcaggcacatggtgtcgatgttagtcaggttcgacacaagctgacgctgctggtcaaccaaaatgtagatggtgaagcagcaagggcagaagct
cgcgtgtatttggaagagtttgtccgtgaatcttactcaaataccgactttgagcaaaaaatgggagagctgttgtcagaaaatgccatcggtacttat
gaagaaagtactcaggcagcgcgagttgcgattgagtgttgtggtgccgcggacctattgatgtcttttgagtcgatggaagataaagcgcagcaaag
agcggttatcgatgtggtaaacgccaacatcgtcaaataccactcgtaaaagcttgatccggctgctaacaaagcccgaaaggaagctgagttggct
gctgccaccgctgagcaataactagcataaccccttggggcctctaaacgggtcttgaggggttttttgctgaaaggaggaactatatccggatctggc
gtaatagcgaagaggcccgcaccgatcgcccttcccaacagttgctcgaggtac

Supplemental Fig. S1. Physical map (A) and nucleotide sequence (B) of the P_{psbA}^{Ah}::*luxAB* expression plasmid p6803*PsbA-luxAB*.

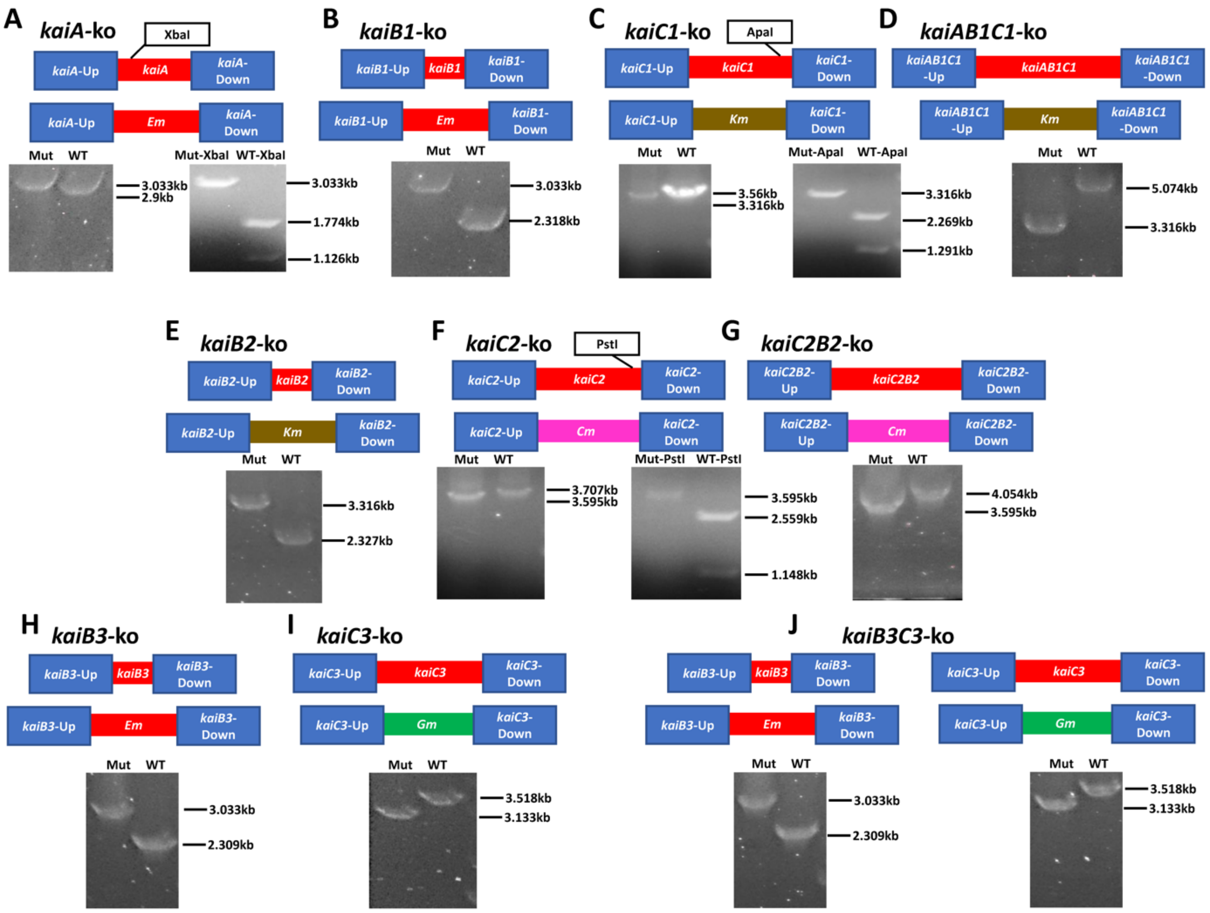

Supplemental Figure S2. The genotyping by colony PCR for all the *kai*-ko mutants. The 5' primer of the 1kb upstream region and the 3' primer of the 1kb downstream region were used to amplify both the wild type (WT) and the null mutant sequences. Abbrevations: Em = Erythromycin, Km = Kanamycin, Cm = Chloramphenicol, and Gm = Gentamycin. **A.** The resulting up-*kaiA*-down for WT and up-*Em*-down sequences had similar sizes, so XbaI (whose restriction site was only contained within the *kaiA* ORF) was used to digest both the WT and null mutant fragments. Only the WT fragment was cut into 2 smaller bands. **B.** PCR fragments for WT and *kaiB1* null mutants. **C.** PCR fragments for WT and *kaiC1* null mutants. The sizes were similar so ApaI (whose restriction site was only contained within the *kaiC1* ORF) was used to digest both the WT and null mutant fragments. Only the WT fragment was cut into 2 smaller bands. **D.** PCR fragments for WT and *kaiAB1C1* null mutant. **E.** PCR fragments for WT and *kaiB2* null mutant. **F.** PCR fragments for WT and *kaiC2* null mutant. The sizes were similar so PstI (whose restriction site was only contained within the *kaiC2* ORF) was used to digest both the WT and null mutant fragments. Only the WT fragment was cut into 2 smaller bands. **G.** PCR fragments for WT and *kaiC2B2* null mutant. **H.** PCR fragments for WT and *kaiB3* null mutant. **I.** PCR fragments for WT and *kaiC3* null mutant. **J.** PCR fragments for WT and *kaiB3C3* null mutant. The 2 genes (*kaiB3* & *kaiC3*) are not localized together on the genome so for the double null mutant, PCR was conducted on the 2 loci separately. The physical maps and the DNA sequences of all the plasmids for generating null mutants and period mutants are shown in Supplemental Figure S4.

A

126bp intergenic sequence between *kaiA* and *kaiB1*

TAACCAAATGCTACGGTTTAATCATCAGCTCAACTCTTGGCTGTTGAAACTACCTCGATTCTC
TGGTTATCTATTTTCTTAGTTGTTTCTTGGTTGGTTTCCCCCTCGTTCTAGTAAACCCGTAAA

B

Start	End	Score	Promoter Sequence
41	86	0.85	CTGTTGAAACTACCTCGATTCTCTGGTTATCTATTTTCTT**A**GTTGTTTCT

Supplemental Figure S3. The *kaiB1C1* promoter as predicted by “Berkeley Drosophila Genome Project” online program (Reese MG, 2001, “Application of a time-delay neural network to promoter annotation in the *Drosophila melanogaster* genome." *Comput Chem* 26: 51-6.). **A.** The 126bp intergenic region between *kaiA* and *kaiB1*. **B.** The predicted promoter sequence within the 126bp is listed. The predicted transcription starting site is marked in red at the 41th nucleotide, and therefore the *kaiB1C1* promoter is most likely to occur in the 126bp intergenic region between *kaiA* and *kaiB1*.

Supplemental Figure S4. The physical maps and the DNA sequences of all the plasmids for generating null mutants and period mutants. All the PCR fragments were inserted into the linearized pMini-T 2.0 vector from the NEB PCR Cloning Kit(NEB #E1203S), so only the physical maps and DNA sequences of the PCR fragments without the pMini--T 2.0 vectors are provided here.

A. Physical map of the *kaiA*-ko fragment.
B. DNA sequence of the *kaiA*-ko fragment.
C. Physical map of the *kaiB1*-ko fragment.
D. DNA sequence of the *kaiB1*-ko fragment.
E. Physical map of the *kaiC1*-ko fragment.
F. DNA sequence of the *kaiC1*-ko fragment.
G. Physical map of the *kaiAB1C1*-ko fragment.
H. DNA sequence of the *kaiAB1C1*-ko fragment.
I. Physical map of the *kaiB2*-ko fragment.
J. DNA sequence of the *kaiB2*-ko fragment.
K. Physical map of the *kaiC2*-ko fragment.
L. DNA sequence of the *kaiC2*-ko fragment.
M. Physical map of the *kaiC2B2*-ko fragment.
N. DNA sequence of the *kaiC2B2*-ko fragment.
O. Physical map of the *kaiB3*-ko fragment.
P. DNA sequence of the *kaiB3*-ko fragment.
Q. Physical map of the *kaiC3*-ko fragment.
R. DNA sequence of the *kaiC3*-ko fragment.
S. Physical map of the *kaiA*-E103K fragment.
T. DNA sequence of the *kaiA*-E103K fragment.
U. Physical map of the *kaiA*-D119E fragment.
V. DNA sequence of the *kaiA*-D119E fragment.
W. Physical map of the *kaiA*-F224S fragment.
X. DNA sequence of the *kaiA*-F224S fragment.
Y. Physical map of the *kaiC1*-Y402F fragment.
Z. DNA sequence of the *kaiC1*-Y402F fragment.
AA. Physical map of the *kaiC1*-Y402M fragment.
AB. DNA sequence of the *kaiC1*-Y402M fragment.
AC. Physical map of the *kaiC1*-Y402W fragment.
AD. DNA sequence of the *kaiC1*-Y402W fragment.

A. Physical map of the *kaiA*-ko fragment:

***kaiA*-ko**

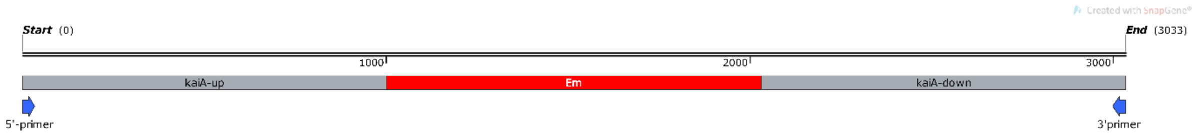

B. DNA sequence of the *kaiA*-ko fragment:

TTTAATGCCTGCTCGTAATCCAGCCGTTTAACTGTTTTATTGCCATTTTTCAGCAACTCATTGGCCCGGTTCTGAATC
CATTGGGTCGCTTCCGGTTCCGCCGGGCCACCGGAGGGGGGATTATATTTAAAGCCTCCATCAGTGGGGGGATTG
TGGGAAGGGGTAATAATAATGCCGTCCGCTAAACCTTCTTTACGTCCCTGGTTGTGGGTCAAAATGGCGTAGGAT
ACCGCCGGAGTGGGGGTGAAACGGGTTAAATCCGTGGCGGTGGTTAAAAAAGTTTCTACTTGGTTAGCGGCCAA
CACTTCCAACGCCGTTTTCTGGGCTGGTTCCGACAGAGCATGGCTATCCATCCCCATATAAAGGGGCCCCGTAATG
CCCTGGGCTTGGCGATAGTCCACCACTGCCTGGGTCACCGCCAAAATATGGGCTTCATTAAAAGTACCGTTGAGG
GCAGAACCCCGATGGCCAGAGGTACCAAAGCTCACTAACTGGGCGGGATTTTCCGGGTCCGGTTGCTGACGGTAA
TAGTCGTCTAAAAGTTTGGCCACATCCAAAAGGCTGTCGGCGGGGGGATGCTGGCCGGCGAGGGGATTAATTCT
GCTTGTCATATACAAAAATTGTAAAAAATGGAGGGCGGCGATCAGGGGCTTAGACACCCAAATCCTAGCCAAAAA
GGGTTAACTAGCCAAGGGCTATCCATGGGCAAAGAGATAAAAGAAAAAGTCTCCAAATCCCTGGTCATAGAGAAA
AAATTGCCAAAGTTACCCCAGGCCATACACGGCCCAGCGCCAAGATGGGGAGCACAAATTCAAACTTTGTAAACA
GGCCGGAAGCTATCCGGCCAAGGAGCACTCAGATTGTGTTAACGTTCAGGGGAGTTGCTTAACACAATTTTCCAAT
TAATAGTATTAATATTTTCTTAACTTGCACCGTACCATGGTGAGAAAGCCTATCTGAGCCCTTATTTGATTAACCTTC
GACTGATTATTGATCCCCTgcgtgctataattatactaattttataaggaggaaaaaatatgggcatttttagtatttttgtaatcagcacagttc
attatcaaccaaacaaaaaataagtggttataatgaatcgttaataagcaaaattcatataaccaaattaaagagggttataatgaacgagaaaaat
ataaaacacagtcaaaactttattacttcaaaacataatatagataaaataatgacaaatataagattaaatgaacatgataatatctttgaaatcgg
ctcaggaaaaggccattttacccttgaattagtaaagaggtgtaatttcgtaactgccattgaaatagaccataaattatgcaaaactacagaaaata
aacttgttgatcacgataatttccaagttttaaacaaggatatattgcagtttaaatttcctaaaaaccaatcctataaaatatacggtaatataccttat
aacataagtacggatataatacgcaaaattgtttttgatagtatagctaatgagatttatttaatcgtggaatacgggtttgctaaaagattattaaata
caaaacgctcattggcattacttttaatggcagaagttgatatttctatattaagtatggttccaagagaatattttcatcctaaacctaaagtgaatagc
tcacttatcagattaagtagaaaaaaatcaagaatatcacacaaagataaacaaaagtataattatttcgttatgaaatgggttaacaaagaataca
agaaaatatttacaaaaaatcaatttaacaattccttaaaacatgcaggaattgacgatttaaacaatattagctttgaacaattcttatctcttttcaat
agctataaattatttaataagtaagttaagggatgcagggcggtgtgaaataccgcacagatgcgtaaggagaaaataccgcatcaggcgctcttcc
gcttcctcgctcactgactcgctgcgctcggtcgttcggctgcggcgagcggtatcagctcactcaTAACCAAATGCTACGGTTTAATCATC
AGCTCAACTCTTGGCTGTTGAAACTACCTCGATTCTCTGGTTATCTATTTTCTTAGTTGTTTCTTGGTTGGTTTCCCCC
TCGTTCTAGTAAACCCGTAAAATGAGCCCCTTTAAAAAAACTTACGTTCTCAAACTCTACGTAGCTGGCAACACCCC
CAACTCTGTGCGGGCCTTAAAAATGCTAAAAAATATCCTTGAGCAAGAATTCCAGGGAGTTTATGCCCTCAAAGTA
ATCGACGTGTTGAAAAATCCCCAATTAGCCGAAGAAGATAAAATTCTTGCCACCCCCACCTTGGCTAAAATCCTACC
GCCCCCTGTCAGGAAAATCATCGGCGACCTTTCCGACCGAGAGAAAGTATTGATTGGTTTAGACCTGCTCTATGAC
GAAATTCGGGAACGGGAAGCAGAAGACCAATAGAAAATCGGGGAACAGGGCGAGATATTTCCCCCATCAACACA
CATCATCAGACTCCTAACAAACTTTAATCCTGCCCCTCAAACTCAACACATTCAATCAGTTTTAGTCAAACTGTTATT
TAGTTTTAATTTTACTAACTTTTTTCTTCTTTAAAAATCTAAATTTAGCTAATGAACTTACCGATTGTTAACGAACGTA
ATCGCCCCGATGTGCCAAGGAAGGGAGTGCAAAAAATTCGTACTGTGATCGAGGGCTTTGACGAAATTACCCACG
GCGGTTTACCCATTGGCCGTACAACCCTGGTGAGTGGCACCTCCGGCACAGGCAAAACTCTCTTGGCAGTACAATT
TCTTTACCAAGGCATTCACCATTTCGATTATCCGGGTTTATTCATTACATTTGAAGAATCCCCCAGTGACATTATTGA
AAATGCCTATAGTTTTGGCTGGGATTTACAACAATTAATTGACGATGGCAAATTGTTTATCCTCGATGCTTCCCCCG
ATCCGGAAGGGCAGGAAGTGGTGGGCACCTTTGATCTGTCGGCCTTAATTGA

C. Physical map of the *kaiB1*-ko fragment:

***kaiB1*-ko**

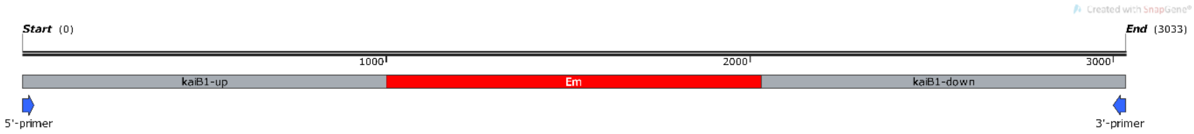

D. DNA sequence of the *kaiB1*-ko fragment:

TTTTGCTCCCGAACACGTTGCCCATAGACTCAGGTCTATTTTCCAGGGCGATCGCCATTACCTATCGACTTTTCAAG
CACTAGATGATTTTTGTGCCTTTCTAGAAGACAAACCTGAGCGGATTGATTGCCTGTTAGTCTATTACGAAGCTAAT
TCCCTTCCAGTGCTGAATCGTCTCTATGAACAGGGGCGATTGTTGCCGATTATTTTGCTCGAACCCAGTCCTTCTGC
CCTAGCCAAAACCACCGACGAACACCCCACCATTGTCTATCACAACGCTGAAATTCATCTGCCCGAATCCCAATGGT
CGGAACTGCCCACCGTCGTAGACCGGGCGATCGCCCATTACCTACACCTTGGCCCCATCTGTACCCTCCCCAACCA
AACGGAAACTATCCCCGCCCCGATTGTCGATGAATCATCCCAAAGCTTTTTACTCCTACAACAAAGAAGGCTGGCT
GACAAACTTAAAGAAAGACTCGGTTACCTAGGAGTGTACTACAAACGTAAGCCCAGTCACTTTTACCGCAACTTTT
CCCCCCAGGAAAAACAAGAATACCTAGAAGATTTAAGCTCCCAATATCGAGAGATTATTCTCAGTTATTTTAGTGA
CGAAGGCACAGTTAATGACCTGTTAGATCAATTTGTTAATCAGGCTTTCTTTGCCGACCTAGCCATTTCTCAAATCC
TGGAAATTCACATGGAATTAATGGATGAATTTTCCCAGCATCTAAAGCTAGAAGGGCGGAGCGAAGAAGTCCTCC
TAGACTATCGTTTAGTGTTGATCGACATCCTCGCCCATCTGGGGGAAATGTATCGCCGTTCCATCCCCCGGGAGGA
CATTCCCTTTGATGTATATTATCAGACGGATTAATAACCAAATGCTACGGTTTAATCATCAGCTCAACTCTTGGCTGT
TGAAACTACCTCGATTCTCTGGTTATCTATTTTCTTAGTTGTTTCTTGGTTGGTTTCCCCCTCGTTCTAGTAAACCCGT
AAAgcgtgctataattatactaattttataaggaggaaaaaatatgggcatttttagtatttttgtaatcagcacagttcattatcaaccaaacaaaaa
ataagtggttataatgaatcgttaataagcaaaattcatataaccaaattaaagagggttataatgaacgagaaaaatataaaacacagtcaaaact
ttattacttcaaaacataatatagataaaataatgacaaatataagattaaatgaacatgataatatctttgaaatcggctcaggaaaaggccatttta
cccttgaattagtaaagaggtgtaatttcgtaactgccattgaaatagaccataaattatgcaaaactacagaaaataaacttgttgatcacgataatt
tccaagttttaaacaaggatatattgcagtttaaatttcctaaaaaccaatcctataaaatatacggtaatataccttataacataagtacggatataat
acgcaaaattgtttttgatagtatagctaatgagatttatttaatcgtggaatacgggtttgctaaaagattattaaatacaaaacgctcattggcattac
ttttaatggcagaagttgatatttctatattaagtatggttccaagagaatattttcatcctaaacctaaagtgaatagctcacttatcagattaagtaga
aaaaaatcaagaatatcacacaaagataaacaaaagtataattatttcgttatgaaatgggttaacaaagaatacaagaaaatatttacaaaaaatc
aatttaacaattccttaaaacatgcaggaattgacgatttaaacaatattagctttgaacaattcttatctcttttcaatagctataaattatttaataagt
aagttaagggatgcagggcggtgtgaaataccgcacagatgcgtaaggagaaaataccgcatcaggcgctcttccgcttcctcgctcactgactcgct
gcgctcggtcgttcggctgcggcgagcggtatcagctcactcaAAAATCGGGGAACAGGGCGAGATATTTCCCCCATCAACACAC
ATCATCAGACTCCTAACAAACTTTAATCCTGCCCCTCAAACTCAACACATTCAATCAGTTTTAGTCAAACTGTTATTT
AGTTTTAATTTTACTAACTTTTTTCTTCTTTAAAAATCTAAATTTAGCTAATGAACTTACCGATTGTTAACGAACGTAA
TCGCCCCGATGTGCCAAGGAAGGGAGTGCAAAAAATTCGTACTGTGATCGAGGGCTTTGACGAAATTACCCACGG
CGGTTTACCCATTGGCCGTACAACCCTGGTGAGTGGCACCTCCGGCACAGGCAAAACTCTCTTGGCAGTACAATTT
CTTTACCAAGGCATTCACCATTTCGATTATCCGGGTTTATTCATTACATTTGAAGAATCCCCCAGTGACATTATTGAA
AATGCCTATAGTTTTGGCTGGGATTTACAACAATTAATTGACGATGGCAAATTGTTTATCCTCGATGCTTCCCCCGA
TCCGGAAGGGCAGGAAGTGGTGGGCACCTTTGATCTGTCGGCCTTAATTGAAAGAATTCAGTATGCAGTGCGGAA
ATATAAAGCCAAGTTAGTTTCCATTGATTCGGTCACAGCGGTATTTCAACAATATGATGCGGCTTCGGTGGTGCGG

CGGGAAATTTTTCGTTTGGTGGCTAGGTTAAAACAGCTCCAGGTAACGTCCATTATGACCACCGAACGGGTGGAA
GAATATGGCCCCATTGCCCGCTTTGGCGTAGAGGAATTCGTCTCCGATAACGTGGTGGTTTTGCGTAATGTTTTAG
AAGGGGAACGGCGACGACGCACGGTGGAAATCCTCAAACTACGGGGTACCACCCACATGAAGGGGGAATATCCT
TTCACTATCACCCACGACGGCATTAACATTTTTCCCCTGGGAGCCATGCGCCTCACCCAGAGGTCTTCCAATGCCCG
CATTTCATCGGGAGTACAAACCTTGGACGAAATGTGTGGCGG

E. Physical map of the *kaiC1*-ko fragment:

***kaiC1*-ko**

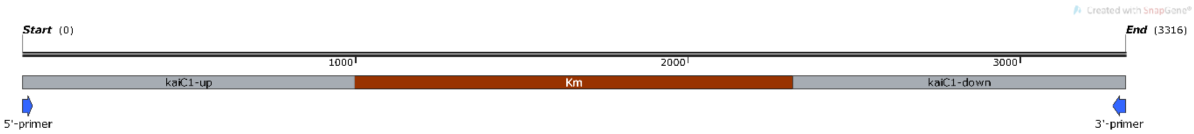

F. DNA sequence of the *kaiC1*-ko fragment:

TAGGAGTGTACTACAAACGTAAGCCCAGTCACTTTTACCGCAACTTTTCCCCCCAGGAAAAACAAGAATACCTAGA
AGATTTAAGCTCCCAATATCGAGAGATTATTCTCAGTTATTTTAGTGACGAAGGCACAGTTAATGACCTGTTAGATC
AATTTGTTAATCAGGCTTTCTTTGCCGACCTAGCCATTTCTCAAATCCTGGAAATTCACATGGAATTAATGGATGAA
TTTTCCCAGCATCTAAAGCTAGAAGGGCGGAGCGAAGAAGTCCTCCTAGACTATCGTTTAGTGTTGATCGACATCC
TCGCCCATCTGGGGGAAATGTATCGCCGTTCCATCCCCCGGGAGGACATTCCCTTTGATGTATATTATCAGACGGA
TTAATAACCAAATGCTACGGTTTAATCATCAGCTCAACTCTTGGCTGTTGAAACTACCTCGATTCTCTGGTTATCTAT
TTTCTTAGTTGTTTCTTGGTTGGTTTCCCCCTCGTTCTAGTAAACCCGTAAAATGAGCCCCTTTAAAAAAACTTACGT
TCTCAAACTCTACGTAGCTGGCAACACCCCCAACTCTGTGCGGGCCTTAAAAATGCTAAAAAATATCCTTGAGCAA
GAATTCCAGGGAGTTTATGCCCTCAAAGTAATCGACGTGTTGAAAAATCCCCAATTAGCCGAAGAAGATAAAATTC
TTGCCACCCCCACCTTGGCTAAAATCCTACCGCCCCCTGTCAGGAAAATCATCGGCGACCTTTCCGACCGAGAGAA
AGTATTGATTGGTTTAGACCTGCTCTATGACGAAATTCGGGAACGGGAAGCAGAAGACCAATAGAAAATCGGGG
AACAGGGCGAGATATTTCCCCCATCAACACACATCATCAGACTCCTAACAAACTTTAATCCTGCCCCTCAAACTCAA
CACATTCAATCAGTTTTAGTCAAACTGTTATTTAGTTTTAATTTTACTAACTTTTTTCTTCTTTAAAAATCTAAATTTAG
CTAcacgctgccgcaagcactcagggcgcaagggctgctaaaggaagcggaacacgtagaaagccagtccgcagaaacggtgctgaccccggat
gaatgtcagctactgggctatctggacaagggaaaacgcaagcgcaaagagaaagcaggtagcttgcagtgggcttacatggcgatagctagactg
ggcggttttatggacagcaagcgaaccggaattgccagctggggcgccctctggtaaggttgggaagccctgcaaagtaaactggatggctttcttgc
cgccaaggatctgatggcgcaggggatcaagatctgatcaagagacaggatgaggatcgtttcgcatgattgaacaagatggattgcacgcaggttc
tccggccgcttgggtggagaggctattcggctatgactgggcacaacagacaatcggctgctctgatgccgccgtgttccggctgtcagcgcaggggc
gcccggttctttttgtcaagaccgacctgtccggtgccctgaatgaactgcaggacgaggcagcgcggctatcgtggctggccacgacgggcgttcctt
gcgcagctgtgctcgacgttgtcactgaagcgggaagggactggctgctattgggcgaagtgccggggcaggatctcctgtcatctcaccttgctcctg
ccgagaaagtatccatcatggctgatgcaatgcggcggctgcatacgcttgatccggctacctgcccattcgaccaccaagcgaaacatcgcatcgag
cgagcacgtactcggatggaagccggtcttgtcgatcaggatgatctggacgaagagcatcaggggctcgcgccagccgaactgttcgccaggctca

aggcgcgcatgcccgacggcgaggatctcgtcgtgacccatggcgatgcctgcttgccgaatatcatggtggaaaatggccgcttttctggattcatcg
actgtggccggctgggtgtggcggaccgctatcaggacatagcgttggctacccgtgatattgctgaagagcttggcggcgaatgggctgaccgcttc
ctcgtgctttacggtatcgccgctcccgattcgcagcgcatcgccttctatcgccttcttgacgagttcttctgagcgggactctggggttcgaaatgacc
gaccaagcgacgcccaacctgccatcacgagatttcgattccaccgccgccttctatgaaaggttgggcttcggaatcgttttccgggacgccggctgg
atgatcctccagcgcggggatctcatgctggagttcttcgcccaccGATTGAAAAAGGGTAAACTTTCCCATCCGAATCTTGGTTGG
GAAAGGCGGCATGGACAGTGGTTAAGGGTTAGTTTTCTGGGGAATTTAATAACTAAATGTTCCCAGCAAAATCCA
GACTGATGAGAAGATCGTTAAAGTCTCGATCGCCACCGCCGAATAGATCCTCAACTCCATAAAGGGATTGTCCAAG
ACGCATGAAGTGACTTTCGTTATCTGGATTGGCTTGGTCGTACATAAAGAAAGCGTTGACATTGCCTGGTTGATTA
CCGGGGTTTTGTTGGAGGAACTGCTCAGTGGTTCCATTGGAGATGAGGAACAAGCCGTAATTACCCCCTGCTTTTA
ACTGAGTGGTCGCCGTTTTTTGGGAAAAATTGTCTGGGGCACTAAAGGTGATGCCTCCAGTTAAAGGATCTCGACT
CTGGGTCATGCGAACCGCCGCTTCAGCATAGCCCGCTTGCCCTGGCGAGAAATCGATTATGCCATCGTTATTGATA
TCAATATCCCCATTGGCATTAGCGAGTTTATACAAACCAAGGGTGTTTTGATATTCACCAGCACGATGGATTTGAAT
ATCAGTGGCGATCGCCTCTCCTTGCAAACCGGGGACAACGAGGGGAAGGGTAAAAACTTCGACGGCATATTTCCC
GTTGGGGGTGTCTGCGGAAATTTGGGTAAAACCCCGACCATCCTGGGTCACTGACAAATTCTCATCCTCAACAGAA
AACATTTCGTTACTGTTCAAGTCAAGGATATAGGGAGTCAAACTATAAGCATCCTCAATGGTGACCTGGGAAAACG
TACCGATGTCCTTATGTTCGGTGGAAGCCAGGGAATTACCGGAAGTAAGCACTTGTTGAGCCAGAGTTCGGTTTA
AGGCCAAACCATCCGCCGGAGCGGCTTCCAAAACAGGGCTAAATGAACTCCGTTCACCGTTACGAAAAACCCCGG
CTTGGGTTTCGAGGGGGATAAAAGCTGACCAACCTTCATCGGTGGGATCA

G. Physical map of the *kaiAB1C1*-ko fragment:

***kaiAB1C1*-ko**

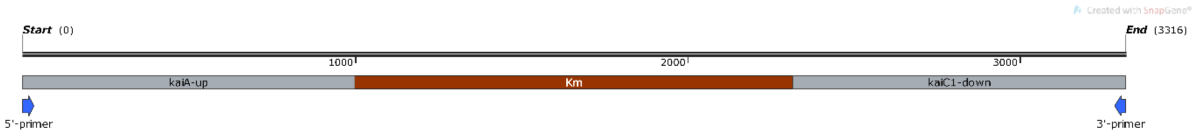

H. DNA sequence of the *kaiAB1C1*-ko fragment:

TTTAATGCCTGCTCGTAATCCAGCCGTTTAACTGTTTTATTGCCATTTTTCAGCAACTCATTGGCCCGGTTCTGAATC
CATTGGGTCGCTTCCGGTTCCGCCGGGCCACCGGAGGGGGGATTATATTTAAAGCCTCCATCAGTGGGGGGATTG
TGGGAAGGGGTAATAATAATGCCGTCCGCTAAACCTTCTTTACGTCCCTGGTTGTGGGTCAAAATGGCGTAGGAT
ACCGCCGGAGTGGGGGTGAAACGGGTTAAATCCGTGGCGGTGGTTAAAAAAGTTTCTACTTGGTTAGCGGCCAA
CACTTCCAACGCCGTTTTCTGGGCTGGTTCCGACAGAGCATGGCTATCCATCCCCATATAAAGGGGCCCCGTAATG
CCCTGGGCTTGGCGATAGTCCACCACTGCCTGGGTCACCGCCAAAATATGGGCTTCATTAAAAGTACCGTTGAGG
GCAGAACCCCGATGGCCAGAGGTACCAAAGCTCACTAACTGGGCGGGATTTTCCGGGTCCGGTTGCTGACGGTAA
TAGTCGTCTAAAAGTTTGGCCACATCCAAAAGGCTGTCGGCGGGGGGATGCTGGCCGGCGAGGGGATTAATTCT
GCTTGTCATATACAAAAATTGTAAAAAATGGAGGGCGGCGATCAGGGGCTTAGACACCCAAATCCTAGCCAAAAA
GGGTTAACTAGCCAAGGGCTATCCATGGGCAAAGAGATAAAAGAAAAAGTCTCCAAATCCCTGGTCATAGAGAAA
AAATTGCCAAAGTTACCCCAGGCCATACACGGCCCAGCGCCAAGATGGGGAGCACAAATTCAAACTTTGTAAACA

GGCCGGAAGCTATCCGGCCAAGGAGCACTCAGATTGTGTTAACGTTCAGGGGAGTTGCTTAACACAATTTTCCAAT
TAATAGTATTAATATTTTCTTAACTTGCACCGTACCATGGTGAGAAAGCCTATCTGAGCCCTTATTTGATTAACCTTC
GACTGATTATTGATCCCCTcacgctgccgcaagcactcagggcgcaagggctgctaaaggaagcggaacacgtagaaagccagtccgcaga
aacggtgctgaccccggatgaatgtcagctactgggctatctggacaagggaaaacgcaagcgcaaagagaaagcaggtagcttgcagtgggctta
catggcgatagctagactgggcggttttatggacagcaagcgaaccggaattgccagctggggcgccctctggtaaggttgggaagccctgcaaagt
aaactggatggctttcttgccgccaaggatctgatggcgcaggggatcaagatctgatcaagagacaggatgaggatcgtttcgcatgattgaacaag
atggattgcacgcaggttctccggccgcttgggtggagaggctattcggctatgactgggcacaacagacaatcggctgctctgatgccgccgtgttcc
ggctgtcagcgcaggggcgcccggttctttttgtcaagaccgacctgtccggtgccctgaatgaactgcaggacgaggcagcgcggctatcgtggctg
gccacgacgggcgttccttgcgcagctgtgctcgacgttgtcactgaagcgggaagggactggctgctattgggcgaagtgccggggcaggatctcct
gtcatctcaccttgctcctgccgagaaagtatccatcatggctgatgcaatgcggcggctgcatacgcttgatccggctacctgcccattcgaccaccaa
gcgaaacatcgcatcgagcgagcacgtactcggatggaagccggtcttgtcgatcaggatgatctggacgaagagcatcaggggctcgcgccagcc
gaactgttcgccaggctcaaggcgcgcatgcccgacggcgaggatctcgtcgtgacccatggcgatgcctgcttgccgaatatcatggtggaaaatgg
ccgcttttctggattcatcgactgtggccggctgggtgtggcggaccgctatcaggacatagcgttggctacccgtgatattgctgaagagcttggcggc
gaatgggctgaccgcttcctcgtgctttacggtatcgccgctcccgattcgcagcgcatcgccttctatcgccttcttgacgagttcttctgagcgggact
ctggggttcgaaatgaccgaccaagcgacgcccaacctgccatcacgagatttcgattccaccgccgccttctatgaaaggttgggcttcggaatcgtt
ttccgggacgccggctggatgatcctccagcgcggggatctcatgctggagttcttcgcccaccGATTGAAAAAGGGTAAACTTTCCCATC
CGAATCTTGGTTGGGAAAGGCGGCATGGACAGTGGTTAAGGGTTAGTTTTCTGGGGAATTTAATAACTAAATGTT
CCCAGCAAAATCCAGACTGATGAGAAGATCGTTAAAGTCTCGATCGCCACCGCCGAATAGATCCTCAACTCCATAA
AGGGATTGTCCAAGACGCATGAAGTGACTTTCGTTATCTGGATTGGCTTGGTCGTACATAAAGAAAGCGTTGACAT
TGCCTGGTTGATTACCGGGGTTTTGTTGGAGGAACTGCTCAGTGGTTCCATTGGAGATGAGGAACAAGCCGTAAT
TACCCCCTGCTTTTAACTGAGTGGTCGCCGTTTTTTGGGAAAAATTGTCTGGGGCACTAAAGGTGATGCCTCCAGTT
AAAGGATCTCGACTCTGGGTCATGCGAACCGCCGCTTCAGCATAGCCCGCTTGCCCTGGCGAGAAATCGATTATGC
CATCGTTATTGATATCAATATCCCCATTGGCATTAGCGAGTTTATACAAACCAAGGGTGTTTTGATATTCACCAGCA
CGATGGATTTGAATATCAGTGGCGATCGCCTCTCCTTGCAAACCGGGGACAACGAGGGGAAGGGTAAAAACTTCG
ACGGCATATTTCCCGTTGGGGGTGTCTGCGGAAATTTGGGTAAAACCCCGACCATCCTGGGTCACTGACAAATTCT
CATCCTCAACAGAAAACATTTCGTTACTGTTCAAGTCAAGGATATAGGGAGTCAAACTATAAGCATCCTCAATGGT
GACCTGGGAAAACGTACCGATGTCCTTATGTTCGGTGGAAGCCAGGGAATTACCGGAAGTAAGCACTTGTTGAGC
CAGAGTTCGGTTTAAGGCCAAACCATCCGCCGGAGCGGCTTCCAAAACAGGGCTAAATGAACTCCGTTCACCGTT
ACGAAAAACCCCGGCTTGGGTTTCGAGGGGGATAAAAGCTGACCAACCTTCATCGGTGGGATCA

I. Physical map of the *kaiB2*-ko fragment:

***kaiB2*-ko**

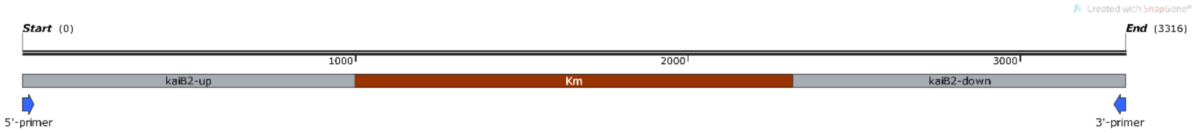

J. DNA sequence of the *kaiB2*-ko fragment:

AGAGCGATGAAAACGTTTCAGTTTGCTCATGGAAAACGGTGTAACAAGGGTGAACACTATCCCATATCACCAGCTC
ACCGTCTTTCATTGCCATACGGAATTCCGGATGAGCATTCATCAGGCGGGCAAGAATGTGAATAAAGGCCGGATA
AAACTTGTGCTTATTTTTCTTTACGGTCTTTAAAAAGGCCGTAATATCCAGCTGAACGGTCTGGTTATAGGTACATT
GAGCAACTGACTGAAATGCCTCAAAATGTTCTTTACGATGCCATTGGGATATATCAACGGTGGTATATCCAGTGAT
TTTTTTCTCCATTTTAGCTTCCTTAGCTCCTGAAAATCTCGATAACTCAAAAAATACGCCCGGTAGTGATCTTATTTC
ATTATGGTGAAAGTTGGAACCTCTTACGTGCCGATCAACGTCTCATTTTCGCCAAAAGTTGGCCCAGGGCTTCCCG
GTATCAACAGGGACACCAGGATTTATTTATTCTGCGAAGTGATCTTCCGTCACAGGTATTTATTCGAAGACGAAAG
GGCCTCGTGATACGCCTATTTTTATAGGTTAATGTCATGATAATAATGGTTTCTTAGACGTCAGGTGGCACTTTTCG
GGGAAATGTGCGCGGAACCCCTATTTGTTTATTTTTCTAAATACATTCAAATATGTATCCGCTCATGAGACAATAAC
CCTGATAAATGCTTCAATAATATTGAAAAAGGAAGAGTATGAGTATTCAACATTTCCGTGTCGCCCTTATTCCCTTT
TTTGCGGCATTTTGCCTTCCTGTTTTTGCTCACCCAGAAACGCTGGTGAAAGTAAAAGATGCTGAAGATCAGTTGG
GTGCACGAGTGGGTTACATCGAACTGGATCTCAACAGCGGTAAGATCCTTGAGAGTTTTCGCCCCGAAGAACGTT
TTCCAATGATGAGCACTTTTAAAGTTCTGCTATGTGGCGCGGTATTATCCCGTGTGACGGATCTGTACTGAAGAGT
CTTCTATTcacgctgccgcaagcactcagggcgcaagggctgctaaaggaagcggaacacgtagaaagccagtccgcagaaacggtgctgaccc
cggatgaatgtcagctactgggctatctggacaagggaaaacgcaagcgcaaagagaaagcaggtagcttgcagtgggcttacatggcgatagcta
gactgggcggttttatggacagcaagcgaaccggaattgccagctggggcgccctctggtaaggttgggaagccctgcaaagtaaactggatggcttt
cttgccgccaaggatctgatggcgcaggggatcaagatctgatcaagagacaggatgaggatcgtttcgcatgattgaacaagatggattgcacgca
ggttctccggccgcttgggtggagaggctattcggctatgactgggcacaacagacaatcggctgctctgatgccgccgtgttccggctgtcagcgcag
gggcgcccggttctttttgtcaagaccgacctgtccggtgccctgaatgaactgcaggacgaggcagcgcggctatcgtggctggccacgacgggcgt
tccttgcgcagctgtgctcgacgttgtcactgaagcgggaagggactggctgctattgggcgaagtgccggggcaggatctcctgtcatctcaccttgct
cctgccgagaaagtatccatcatggctgatgcaatgcggcggctgcatacgcttgatccggctacctgcccattcgaccaccaagcgaaacatcgcat
cgagcgagcacgtactcggatggaagccggtcttgtcgatcaggatgatctggacgaagagcatcaggggctcgcgccagccgaactgttcgccagg
ctcaaggcgcgcatgcccgacggcgaggatctcgtcgtgacccatggcgatgcctgcttgccgaatatcatggtggaaaatggccgcttttctggattc
atcgactgtggccggctgggtgtggcggaccgctatcaggacatagcgttggctacccgtgatattgctgaagagcttggcggcgaatgggctgaccg
cttcctcgtgctttacggtatcgccgctcccgattcgcagcgcatcgccttctatcgccttcttgacgagttcttctgagcgggactctggggttcgaaatg
accgaccaagcgacgcccaacctgccatcacgagatttcgattccaccgccgccttctatgaaaggttgggcttcggaatcgttttccgggacgccggc
tggatgatcctccagcgcggggatctcatgctggagttcttcgcccaccCATTTTAGGTTGTCGCAATGAATGTTCCGGAGGATTTAA
AACCCGCAATTACAGAAATATTTGAGCACCTTCTTACAAAATCCAAAGAACAGTATTACGTACTGCGGCTTTGCATT
GCAGGGAGCAAATTCTAGTCTTTGCAAGCCTAGGAAAGTGTTTGAAAAGTTTTAAGCAAGCCTTTGAGGCATAAG
ATGAACCATGGCAACATAGAGCATTGTCTCACTGGTGGTAGGTAAATATTCATAATCCTTACTTAAACGACGATAA
CGTCCAAACCAAGCAAAGGTGCGCTCGACTGCCCAACGGCGGGGTAGGACTTCAAAGCCTTTTTGTCCCTACTTTT
TGCTGACCACATTCAAGGTCCAACCAAAAGTATGCTCCGCCCAATGGATAAAATCCTTTCCGCCAAAGGTACTGTC
AGTCCATATAACTTGCAGGCACTGCCAAAGGGGAGCAAACCAGGTGCCAAGTAGAATCAGACCCTGATGGTCGG
AGCGATGGGCACCATGGACGACAACATCGAGAATTAATCCCATGGTATCCACAAGGATTGTACGTTTACGACCATT
GACCTTTTTTCCACCGTCATAGCCAGGTTCTTGGCCAGTTCAAGCCCTTTTGAGTGACTGTGAATCTAAACAACCGG
CACTAGGGTGAGTATTTCTTCCAGCTTTGAGCCGAACTTTCTCACGGAAGATACGGTTTAATTTTTTCCAGGTACCA
TCTTCGTGCCATGGCTGGAAATAACCATAGACCGTTCGCCATTTAGGAAAATCGTGGGGTACAAGTCGCCAGGCA
CACCCCGTTCTGAGCATGTAAAAAGCAGAAAGTTTTAGTGGTCAGAAAAAGCCCACGAGCTTACCAGTTGGTAAA
AGTTAAGAAATTTTAACTCCCCGTTTTCACTGCAATTATGAGAAGATAATGAAAAATGAGATGATCATGAAAGACC
ATTATCACTATGGCAACATCATTTGCTTCCCGGGGAGGACTGCTGGCGTCT

K. Physical map of the *kaiC2*-ko fragment:

***kaiC2*-ko**

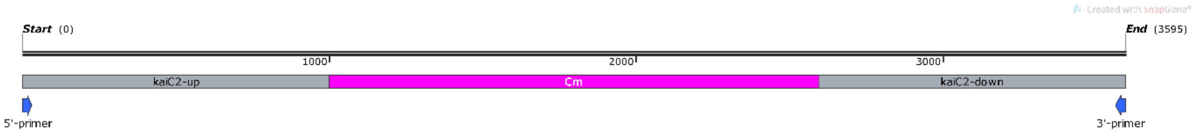

L. DNA sequence of the *kaiC2*-ko fragment:

TTCAAAATGCCTTGGTCACAGGCGGCGGCGGTGATTTTGGCTAGGGTCTGCCGTTGGGCCAAAGTTTGTAGCTCC
GGAACAGTGTCGGGTCCTAGGTTTAAAAATCCCCGGTCATAGTGGTAAATGCGGGAGCGTTCCACATCAATTTTTT
GGTCAAGAATTTGCGGCGGCGGCAGGGTGACAATTAGCGTTTCTCCCTGTTGTTGAATACTTTGTTCATTTATCTCG
GCTAGATCAATGCCGGCCCGCACTTCCCCGTAGCCAATGTAGAGTAAGTCGGTGCGCCCCAGGGTGAAATTACCC
CATTGCCGTTCCTGACTAGTGGGCACTACGGTTTCCATGGCAAAAATGGTGGTGGTTAGTTCACTCACTGCCCGGA
TTTGCTGCACCACGAGGGTAGAAATTTCCGCCTTGGCCTGATTATTGCCGATGCGGAGCCAAGGGCTGATGGTAC
GTAGACCCTGGACAATGCCCATAACGGCGATCGCCATGGTTAGAACTACGCCCCCAGTCCCCAGAAGCGGTAGTC
GCTGAAAAATGTTCCGGGCTGGTGGTGAGGAAGATAGTTTTTCTGCCATGGTTTAAGCAATGGTTGAGGGGGGA
ATAACTTGATTGTAGTAGCCGGGGATCGCAGTTTTAATGGATACTATTGCCGTGACTGTATGGGTCCATGGGGCCA
ATTCCATGGTCAACTAGCTTCTAAATGGAACTCAGGCGGAAAATTAGGTAAATTTCACCATACCCGACTAAGGCAC
TTTCTGTTGCTGGGGTCTGGGTCCGCTAATGTGACAAAAAGTTATTACTGAAGTCATGGGGTTGGCCTTGGTCGGG
CAGTTGACAACAATGGCGATCGGAGTAGCCCATGCCTACGGTGTGATCATTGCTCAATCTCGGCAAATTAATAGTG
GGAATATTGTGCGAACATAATTACGACAAAAACTCAAACATAAATTTAAACACCATTGGCAATGCTGGCAAGTAAA
TTGAATCCGTAAAAATCAGATCCGTCGATCATATCGTCAATTATTACCTCCACGGGGAGAGCCTGAGCAAACTGGC
CTCAGGCATTTGAGAAGCACACGGTCACACTGCTTCCGGTAGTCAATAAACCGGTAAACCAGCAATAGACATAAG
CGGCTATTTAACGACCCTGCCCTGAACCGACGACCGGGTCGAATTTGCTTTCGAATTTCTGCCATTCATCCGCTTAT
TATCACTTATTCAGGCGTAGCACCAGGCGTTTAAGGGCACCAATAACTGCCTTAAAAAAATTACGCCCCGCCCTGC
CACTCATCGCAGTACTGTTGTAATTCATTAAGCATTCTGCCGACATGGAAGCCATCACAAACGGCATGATGAACCT
GAATCGCCAGCGGCATCAGCACCTTGTCGCCTTGCGTATAATATTTGCCCATGGTGAAAACGGGGGCGAAGAAGT
TGTCCATATTGGCCACGTTTAAATCAAAACTGGTGAAACTCACCCAGGGATTGGCTGAGACGAAAAACATATTCTC
AATAAACCCTTTAGGGAAATAGGCCAGGTTTTCACCGTAACACGCCACATCTTGCGAATATATGTGTAGAAACTGC
CGGAAATCGTCGTGGTATTCACTCCAGAGCGATGAAAACGTTTCAGTTTGCTCATGGAAAACGGTGTAACAAGGG
TGAACACTATCCCATATCACCAGCTCACCGTCTTTCATTGCCATACGGAATTCCGGATGAGCATTCATCAGGCGGGC
AAGAATGTGAATAAAGGCCGGATAAAACTTGTGCTTATTTTTCTTTACGGTCTTTAAAAAGGCCGTAATATCCAGCT
GAACGGTCTGGTTATAGGTACATTGAGCAACTGACTGAAATGCCTCAAAATGTTCTTTACGATGCCATTGGGATAT
ATCAACGGTGGTATATCCAGTGATTTTTTTCTCCATTTTAGCTTCCTTAGCTCCTGAAAATCTCGATAACTCAAAAAA
TACGCCCGGTAGTGATCTTATTTCATTATGGTGAAAGTTGGAACCTCTTACGTGCCGATCAACGTCTCATTTTCGCC
AAAAGTTGGCCCAGGGCTTCCCGGTATCAACAGGGACACCAGGATTTATTTATTCTGCGAAGTGATCTTCCGTCAC
AGGTATTTATTCGAAGACGAAAGGGCCTCGTGATACGCCTATTTTTATAGGTTAATGTCATGATAATAATGGTTTCT
TAGACGTCAGGTGGCACTTTTCGGGGAAATGTGCGCGGAACCCCTATTTGTTTATTTTTCTAAATACATTCAAATAT
GTATCCGCTCATGAGACAATAACCCTGATAAATGCTTCAATAATATTGAAAAAGGAAGAGTATGAGTATTCAACAT

TTCCGTGTCGCCCTTATTCCCTTTTTTGCGGCATTTTGCCTTCCTGTTTTTGCTCACCCAGAAACGCTGGTGAAAGTA
AAAGATGCTGAAGATCAGTTGGGTGCACGAGTGGGTTACATCGAACTGGATCTCAACAGCGGTAAGATCCTTGAG
AGTTTTCGCCCCGAAGAACGTTTTCCAATGATGAGCACTTTTAAAGTTCTGCTATGTGGCGCGGTATTATCCCGTGT
GACGGATCTGTACTGAAGAGTCTTCTATTATGGAAAATTTAAACGCTCTATCTGGCAATCAAAACTTGGAAGTTTG
GCAACTGCGTCTGTATGTAGCGGGACAAACTCCTAAATCCGTCACAGCTTTTATAAATTTAAAAAAGATTTGTGAA
GAATATCTAAACGGTCAATACCAAATTGAAATCATCGATTTGACCCAACAACCTGAATTGGCGATTGAAGATAGTA
TTTTGGCATTGCCTACTTTAGTAAGAAAATTACCCGAACCAATCAAAAAAATTATTGGTGATTTGTCCAATACAGAA
AAGGTATTAGTGGGCTTACAAATTTTACCCTCTATGGATTGGAAAATCTAGCATTTTAGGTTGTCGCAATGAATGTT
CCGGAGGATTTAAAACCCGCAATTACAGAAATATTTGAGCACCTTCTTACAAAATCCAAAGAACAGTATTACGTAC
TGCGGCTTTGCATTGCAGGGAGCAAATTCTAGTCTTTGCAAGCCTAGGAAAGTGTTTGAAAAGTTTTAAGCAAGCC
TTTGAGGCATAAGATGAACCATGGCAACATAGAGCATTGTCTCACTGGTGGTAGGTAAATATTCATAATCCTTACT
TAAACGACGATAACGTCCAAACCAAGCAAAGGTGCGCTCGACTGCCCAACGGCGGGGTAGGACTTCAAAGCCTTT
TTGTCCCTACTTTTTGCTGACCACATTCAAGGTCCAACCAAAAGTATGCTCCGCCCAATGGATAAAATCCTTTCCGC
CAAAGGTACTGTCAGTCCATATAACTTGCAGGCACTGCCAAAGGGGAGCAAACCAGGTGCCAAGTAGAATCAGAC
CCTGATGGTCGGAGCGATGGGCACCATGGACGACAACATCGAGAATTAATCCCATGGTATCCACAAGGATTGTAC
GTTTACGACCATTGACCTTTTTTCCACCGTCATAGCCAGGTTCTTGGCCAGTTCAAGCCCTTTTGAGTGACTGTGAA
TCTAAACAACCGGCACTAGG

M. Physical map of the *kaiC2B2*-ko fragment:

***kaiC2B2*-ko**

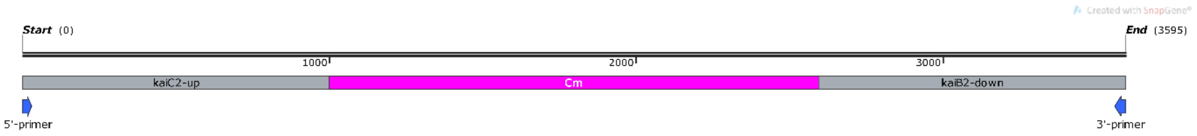

N. DNA sequence of the *kaiC2B2*-ko fragment:

TTCAAAATGCCTTGGTCACAGGCGGCGGCGGTGATTTTGGCTAGGGTCTGCCGTTGGGCCAAAGTTTGTAGCTCC
GGAACAGTGTCGGGTCCTAGGTTTAAAAATCCCCGGTCATAGTGGTAAATGCGGGAGCGTTCCACATCAATTTTTT
GGTCAAGAATTTGCGGCGGCGGCAGGGTGACAATTAGCGTTTCTCCCTGTTGTTGAATACTTTGTTCATTTATCTCG
GCTAGATCAATGCCGGCCCGCACTTCCCCGTAGCCAATGTAGAGTAAGTCGGTGCGCCCCAGGGTGAAATTACCC
CATTGCCGTTCCTGACTAGTGGGCACTACGGTTTCCATGGCAAAAATGGTGGTGGTTAGTTCACTCACTGCCCGGA
TTTGCTGCACCACGAGGGTAGAAATTTCCGCCTTGGCCTGATTATTGCCGATGCGGAGCCAAGGGCTGATGGTAC
GTAGACCCTGGACAATGCCCATAACGGCGATCGCCATGGTTAGAACTACGCCCCCAGTCCCCAGAAGCGGTAGTC
GCTGAAAAATGTTCCGGGCTGGTGGTGAGGAAGATAGTTTTTCTGCCATGGTTTAAGCAATGGTTGAGGGGGGA
ATAACTTGATTGTAGTAGCCGGGGATCGCAGTTTTAATGGATACTATTGCCGTGACTGTATGGGTCCATGGGGCCA
ATTCCATGGTCAACTAGCTTCTAAATGGAACTCAGGCGGAAAATTAGGTAAATTTCACCATACCCGACTAAGGCAC
TTTCTGTTGCTGGGGTCTGGGTCCGCTAATGTGACAAAAAGTTATTACTGAAGTCATGGGGTTGGCCTTGGTCGGG
CAGTTGACAACAATGGCGATCGGAGTAGCCCATGCCTACGGTGTGATCATTGCTCAATCTCGGCAAATTAATAGTG

GGAATATTGTGCGAACATAATTACGACAAAAACTCAAACATAAATTTAAACACCATTGGCAATGCTGGCAAGTAAA
TTGAATCCGTAAAAATCAGATCCGTCGATCATATCGTCAATTATTACCTCCACGGGGAGAGCCTGAGCAAACTGGC
CTCAGGCATTTGAGAAGCACACGGTCACACTGCTTCCGGTAGTCAATAAACCGGTAAACCAGCAATAGACATAAG
CGGCTATTTAACGACCCTGCCCTGAACCGACGACCGGGTCGAATTTGCTTTCGAATTTCTGCCATTCATCCGCTTAT
TATCACTTATTCAGGCGTAGCACCAGGCGTTTAAGGGCACCAATAACTGCCTTAAAAAAATTACGCCCCGCCCTGC
CACTCATCGCAGTACTGTTGTAATTCATTAAGCATTCTGCCGACATGGAAGCCATCACAAACGGCATGATGAACCT
GAATCGCCAGCGGCATCAGCACCTTGTCGCCTTGCGTATAATATTTGCCCATGGTGAAAACGGGGGCGAAGAAGT
TGTCCATATTGGCCACGTTTAAATCAAAACTGGTGAAACTCACCCAGGGATTGGCTGAGACGAAAAACATATTCTC
AATAAACCCTTTAGGGAAATAGGCCAGGTTTTCACCGTAACACGCCACATCTTGCGAATATATGTGTAGAAACTGC
CGGAAATCGTCGTGGTATTCACTCCAGAGCGATGAAAACGTTTCAGTTTGCTCATGGAAAACGGTGTAACAAGGG
TGAACACTATCCCATATCACCAGCTCACCGTCTTTCATTGCCATACGGAATTCCGGATGAGCATTCATCAGGCGGGC
AAGAATGTGAATAAAGGCCGGATAAAACTTGTGCTTATTTTTCTTTACGGTCTTTAAAAAGGCCGTAATATCCAGCT
GAACGGTCTGGTTATAGGTACATTGAGCAACTGACTGAAATGCCTCAAAATGTTCTTTACGATGCCATTGGGATAT
ATCAACGGTGGTATATCCAGTGATTTTTTTCTCCATTTTAGCTTCCTTAGCTCCTGAAAATCTCGATAACTCAAAAAA
TACGCCCGGTAGTGATCTTATTTCATTATGGTGAAAGTTGGAACCTCTTACGTGCCGATCAACGTCTCATTTTCGCC
AAAAGTTGGCCCAGGGCTTCCCGGTATCAACAGGGACACCAGGATTTATTTATTCTGCGAAGTGATCTTCCGTCAC
AGGTATTTATTCGAAGACGAAAGGGCCTCGTGATACGCCTATTTTTATAGGTTAATGTCATGATAATAATGGTTTCT
TAGACGTCAGGTGGCACTTTTCGGGGAAATGTGCGCGGAACCCCTATTTGTTTATTTTTCTAAATACATTCAAATAT
GTATCCGCTCATGAGACAATAACCCTGATAAATGCTTCAATAATATTGAAAAAGGAAGAGTATGAGTATTCAACAT
TTCCGTGTCGCCCTTATTCCCTTTTTTGCGGCATTTTGCCTTCCTGTTTTTGCTCACCCAGAAACGCTGGTGAAAGTA
AAAGATGCTGAAGATCAGTTGGGTGCACGAGTGGGTTACATCGAACTGGATCTCAACAGCGGTAAGATCCTTGAG
AGTTTTCGCCCCGAAGAACGTTTTCCAATGATGAGCACTTTTAAAGTTCTGCTATGTGGCGCGGTATTATCCCGTGT
GACGGATCTCATTTTAGGTTGTCGCAATGAATGTTCCGGAGGATTTAAAACCCGCAATTACAGAAATATTTGAGCA
CCTTCTTACAAAATCCAAAGAACAGTATTACGTACTGCGGCTTTGCATTGCAGGGAGCAAATTCTAGTCTTTGCAA
GCCTAGGAAAGTGTTTGAAAAGTTTTAAGCAAGCCTTTGAGGCATAAGATGAACCATGGCAACATAGAGCATTGT
CTCACTGGTGGTAGGTAAATATTCATAATCCTTACTTAAACGACGATAACGTCCAAACCAAGCAAAGGTGCGCTCG
ACTGCCCAACGGCGGGGTAGGACTTCAAAGCCTTTTTGTCCCTACTTTTTGCTGACCACATTCAAGGTCCAACCAAA
AGTATGCTCCGCCCAATGGATAAAATCCTTTCCGCCAAAGGTACTGTCAGTCCATATAACTTGCAGGCACTGCCAA
AGGGGAGCAAACCAGGTGCCAAGTAGAATCAGACCCTGATGGTCGGAGCGATGGGCACCATGGACGACAACATC
GAGAATTAATCCCATGGTATCCACAAGGATTGTACGTTTACGACCATTGACCTTTTTTCCACCGTCATAGCCAGGTT
CTTGGCCAGTTCAAGCCCTTTTGAGTGACTGTGAATCTAAACAACCGGCACTAGGGTGAGTATTTCTTCCAGCTTTG
AGCCGAACTTTCTCACGGAAGATACGGTTTAATTTTTTCCAGGTACCATCTTCGTGCCATGGCTGGAAATAACCATA
GACCGTTCGCCATTTAGGAAAATCGTGGGGTACAAGTCGCCAGGCACACCCCGTTCTGAGCATGTAAAAAGCAGA
AAGTTTTAGTGGTCAGAAAAAGCCCACGAGCTTACCAGTTGGTAAAAGTTAAGAAATTTTAACTCCCCGTTTTCACT
GCAATTATGAGAAGATAATGAAAAATGAGATGATCATGAAAGACCATTATCACTATGGCAACATCATTTGCTTCCC
GGGGAGGACTGCTGGCGTCT

O. Physical map of the *kaiB3*-ko fragment:

***kaiB3*-ko**

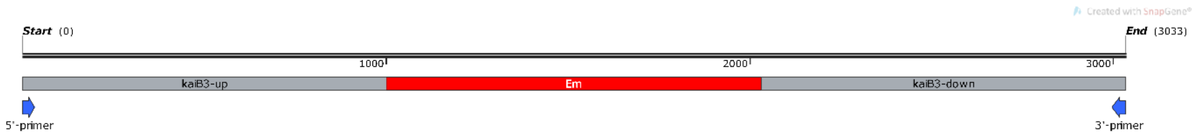

P. DNA sequence of the *kaiB3*-ko fragment:

TCGGACCGTCGATTGTATCCCCTTTGGTGGAACGTCTACCGGTACAAATTACGACGGTGTCAAATTTGGCTGAGGC
GGTGGACATTTGCCAAGATCGAGAGTTTGCCGTGGTTTTATTAGATCTATTTCTATCGGAACTCCAAGGCATTGAT
ACCCTAATTAAAGCCCGCACAATTTTCCCCGACCAATCCATCATTGTCTATAGCCAAAGTGAAGATGAGCATTTAGT
TATTCAAGCGTTCCAGCATGGGGCCGATGGCTATTTACGACTGAAAAATCTCGACAGTTATTTGCTTTATTATGAAT
TACTATCGGTTTTAGAAAGAAATATTTATCGTCGCAAGAGTGAAAATCAACGAAACTTAGCCAGCCAACAGAAGG
AATACAGCGCCCTGGAAACCTTAATTAGCTCCACCACCAGCGTCACTGCCCGGATGTTTGGTTCTGATACCATCAA
AGATTCCTTTCCAGAATTATTTGGGGAGTTGAAACAAACCTATGGAGAGTTGTTAGAATTAGCATTGGATCAACGC
GCTTTTAGGGTAGACCATAATCTTTCTGAGCGCTTGAGAAATTTAGCCGATCGCCTAGGTTTTATGAAAGCCAGCC
CCAGGGATATTATCGAAATCCACACCACCGTCCTAAAGGAAAAGAATCACAATATTAATGTAATTAAATCCCAAGC
CTACGCCACCGAGGGCAGATTGATAGTATTAGAATTAATGGGCTATTTAGCAAGCTTCTATCGCAAATACTATGTC
AGCTTGAATAACTTAAATATTTCCTCGCGTATTTCACCACGATTTCCCCCCAAAGATAGTTCTTAAATTAATTATCTTT
GCTTGATTGTTTAGTTGTTTAACTATAACCTATTACACTTTGTTTTAACTAGATTAATAATCCATTATTTGATTGTTTT
ATTTAAATTTCCTTTAATTCAGGTTACTGACCCCAGATTAATTGAGCTAGATTTAAGCATTGGCTTTATTTGTTATCA
GCGgcgtgctataattatactaattttataaggaggaaaaaatatgggcatttttagtatttttgtaatcagcacagttcattatcaaccaaacaaaaa
ataagtggttataatgaatcgttaataagcaaaattcatataaccaaattaaagagggttataatgaacgagaaaaatataaaacacagtcaaaact
ttattacttcaaaacataatatagataaaataatgacaaatataagattaaatgaacatgataatatctttgaaatcggctcaggaaaaggccatttta
cccttgaattagtaaagaggtgtaatttcgtaactgccattgaaatagaccataaattatgcaaaactacagaaaataaacttgttgatcacgataatt
tccaagttttaaacaaggatatattgcagtttaaatttcctaaaaaccaatcctataaaatatacggtaatataccttataacataagtacggatataat
acgcaaaattgtttttgatagtatagctaatgagatttatttaatcgtggaatacgggtttgctaaaagattattaaatacaaaacgctcattggcattac
ttttaatggcagaagttgatatttctatattaagtatggttccaagagaatattttcatcctaaacctaaagtgaatagctcacttatcagattaagtaga
aaaaaatcaagaatatcacacaaagataaacaaaagtataattatttcgttatgaaatgggttaacaaagaatacaagaaaatatttacaaaaaatc
aatttaacaattccttaaaacatgcaggaattgacgatttaaacaatattagctttgaacaattcttatctcttttcaatagctataaattatttaataagt
aagttaagggatgcagggcggtgtgaaataccgcacagatgcgtaaggagaaaataccgcatcaggcgctcttccgcttcctcgctcactgactcgct
gcgctcggtcgttcggctgcggcgagcggtatcagctcactcaCCGCTATCCCCTAGGCGGTGGGCCGATATTAGGTGATTATTAA
AAACCAGGGCGAAAATTTTGCCCCCATTCCTCTGGCGATCGCCAAAGCTCCAGTTTTTTCCCAATTATGTTGTCCAA
CTCCGACCAGCACCGCGCAGTTCAGGCCCTCTACAACACCTATCCCTTCCCCCCCGAACCCCTGCTACAGGAACCAC
CTCCGGGTTACAACTGGCGCTGGCAATGGACTGCGGCCCATAATTTTTGTTTAGGACGGCGGCCGGCTAATCAAA
AGGTTCGCATTTTGGATGCGGGTTGTGGCACTGGAGTCGGCACGGAATATTTAGTCCACCTCAACCCGGAAGCGG
AAGTTCATGCCGTGGACATCAGTGAAGGGGCTTTAGCCGTTGCCCAAACAAGGTTGCAAAAGTCCGGTGTGGTTT
GTGATCGGGTGCATTTCCACCATTTATCCCTGGAAAATCTGGCCCATCTTCCCGGACAATTTGATTACATCAATTCC

GTCGGTGTACTGCATCATTTGCCCGATCCAGTGGCTGGAATTCAGGCCGTAGCGGAAAAATTGGCCCCCGGTGGT
TTATTCCACATTTTTGTTTACGCTGAAATCGGCCGCTGGGAAATTCAACTCATGCAAAAGGCGATCGCCATCCTACA
GGGGAAAAAACGGGGCGATTACCAGGATGGGGTTGCGGTGGGTCGAGAAATTTTTGCGAGTTTGCCGGAATATA
ACCGTTTAGTGAAAAGGGAAAAAGAGCGTTGGTCCCTGGAAAATCATCGGGATGAATCCTTTGCGGATATGTATG
TCCATCCCCAAGAAACGGACTATAACATTGATACGTTATTTGAATTAATCGACTCGGCTGGACTGGAATTTTTGGG
CTTTTCCAACCCTGATTATTGGCAACTAGATCGCCTGTTAGGCAAAGCACCGGATTTAATGGAACGGGCTAAAGAT
TTAAGCGAAAAGGAACGATATCGTCTAATTGAATTATTAGACCCCGAA

Q. Physical map of the *kaiC3*-ko fragment:

***kaiC3*-ko**

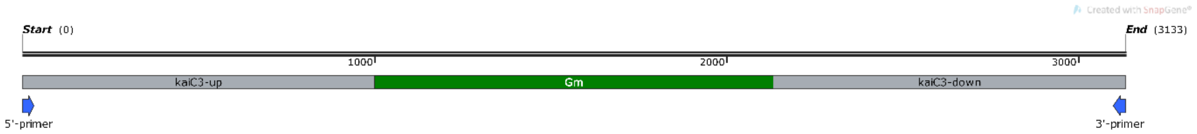

R. DNA sequence of the *kaiC3*-ko fragment:

GGCTCGCTTGGGTTAGGCAGGGCATTATTGACGAAGTGGTGGTGCAGGTTTACGGTTCTACTCCGGCGGAAGTGC
AACAAACCGTTGCCAATTCTGGTATTCATACTGCCTCCCGTTACGTGCCCGTGGGCATTGGCCTCTACACCGGCATT
AAAGCTAAACCATTTAATTTGCAAGCCGTCCAAAACCAGGTTAAGGCTGTGAAAGAGCAAAATCTAGGTCATTCTC
TTTTTGTCTGGGAATTTTTAGTGTTGAGAACAATCAACGCCCATCTCAATGTTCTGTAAGGGTTCTATAAAAATTGT
GAGAAACTCCCCTGGGCAAAGGAGCCATAGCTAGTGTTAGACTCCAGCCTTTTTTTTCCCAAGCTTTCCTTCGTTAT
TTTTTTCTCTTTAGTCCCTAAATTTCCCCTGGATTGAGGGGAATCATGGCCCAGGTGACATAGGTGCCAATGGGGCT
CCAAAGCAAAAACGGTACTAAGAAACCAAAAGCAGTGGTGGAAACTTGACTAACGGCGATCACCAGGGCGAGCC
CCACAAAGAAACCTGTCGCACCAATAATACTGCCCACCCTTAAACTCCGGAGTTTACACATCACGGGGGTGTAGGC
CATGACAGTCAATTCCAGCAGAAGATAGCCGACCATTAATCCCCAGCGATGTCCCGGGTCCGCCGTGGCATTCCAG
GCTAGGGTAGCGGAAATGGCTCCAGCAATGAAAATGGCAATCCAGATGAAGGGGATGGCCCACTCAAAAGTTAG
CCAAGAAGGGCGACGCAGGCGATTGAACCAACGCAAATCCCTGGGGGATAAACGGTTACACACAAAGGCTAGGG
CAAAAGCGATCAACCCAATCCATAGCCAAGCTGGAATCATAAACACTTTCCATTAACTTGTGCAAATTCTATCATCG
CCCCCTGGCTGAGATCGGTGATATCGGTGGCATTATCAACACTGGTATAATTCCCGTTGGTTCCGAATACGAGTAT
GGGGCAGTGAAAGGAGCTATGACCATGATCTACGAATTAGCTTGCATGCCTGCAGGTCGACTCTAGAGGATCCCC
GGGTACCCGGGGATCCGTCGGCCGAGGTCTTCCGATCTCCTGAAGCCAGGGCAGATCCGTGCACAGCACCTTGCC
GTAGAAGAACAGCAAGGCCGCCAATGCCTGACGATGCGTGGAGACCGAAACCTTGCGCTCGTTCGCCAGCCAGG
ACAGAAATGCCTCGACTTCGCTGCTGCCCAAGGTTGCCGGGTGACGCACACCGTGGAAACGGATGAAGGCACGA
ACCCAGTTGACATAAGCCTGTTCGGTTCGTAAACTGTAATGCAAGTAGCGTATGCGCTCACGCAACTGGTCCAGAA
CCTTGACCGAACGCAGCGGTGGTAACGGCGCAGTGGCGGTTTTCATGGCTTGTTATGACTGTTTTTTTGTACAGTC

TATGCCTCGGGCATCCAAGCAGCAAGCGCGTTACGCCGTGGGTCGATGTTTGATGTTATGGAGCAGCAACGATGT
TACGCAGCAGCAACGATGTTACGCAGCAGGGCAGTCGCCCTAAAACAAAGTTAGGTGGCTCAAGTATGGGCATCA
TTCGCACATGTAGGCTCGGCCCTGACCAAGTCAAATCCATGCGGGCTGCTCTTGATCTTTTCGGTCGTGAGTTCGG
AGACGTAGCCACCTACTCCCAACATCAGCCGGACTCCGATTACCTCGGGAACTTGCTCCGTAGTAAGACATTCATC
GCGCTTGCTGCCTTCGACCAAGAAGCGGTTGTTGGCGCTCTCGCGGCTTACGTTCTGCCCAAGTTTGAGCAGCCGC
GTAGTGAGATCTATATCTATGATCTCGCAGTCTCCGGCGAGCACCGGAGGCAGGGCATTGCCACCGCGCTCATCA
ATCTCCTCAAGCATGAGGCCAACGCGCTTGGTGCTTATGTGATCTACGTGCAAGCAGATTACGGTGACGATCCCGC
AGTGGCTCTCTATACAAAGTTGGGCATACGGGAAGAAGTGATGCACTTTGATATCGACCCAAGTACCGCCACCTAA
CAATTCGTTCAAGCCGAGATCGGCTTCCCGGCCGACGGATCCTCTAGAGTCGACCTGCAGGCATGCAAGCTTGGC
ACTGGCCGTCGTTTTACTCAATTCTTCCCTTTGTAAACCTGGAGGCAATCAATCCATGCAGTGGCAAGAATCTTTAC
CCTGGACCCCCGAAGCTCGGCAAAAGTTGAAGAATATTCCCTATTTTGCCCGGGTGCAAGCCCGTCAGCGCATTGA
GCAGTTAGCTCGGCAGGCCGATCTAGATGAGGTCACTGTGGATTTGGTAGAACAAGCCCGTCTGGAGTTTGGCCA
ATGAGTTGGTCTGACTTTTGGGGCTAAACTCACAGGTAAATCCCATTTCACCATGCATGGTTACAAATTCACCAAGC
CGGTCTGACATCAGTGTTTACAATGGCGATCGCCAGGGGCCAATGTATTCTCTGATTGTGCCCATTTATAACGAAG
AAGATAATATTCCCGTGTTATATGAGCGGCTCAAGGCAGTCATGGATCAATTGGCAAGTACGGAACTAGTGCTGA
TTAATGATGGCAGCGGCGATCGATCCTTAGAAATGATTCGGGCTTTGCATGATCAGGATAAACGGGTCTGTTACCT
TAGTTTTGCCCGTAACTTTGGTCATCAAGTGGCGGTGACGGCGGGGCTAAACTTCGCCCGGGGCCAGGCGGTGAT
TATTCTCGATGCGGATTTGCAAGATCCCCCCGAATTGGTGCCCCAATTGGTGGAAAGGTGGCAAGCAGGCTACAG
CGTGGTCTATGCCCAACGGGTTAAACGTCGGCAGGAAAGCTGGTTTAAGCGGCTAACGGCCTATGGGTTCTACCG
ACTATTGCAACGGTTAGCAGATGTAAGAATTCCGGCGGACACGGGGGATTTTTGCCTGATGGACCGCCAAGTGGT
GGATTTGCTCAACACCATGCCGGAAAGAAATCGTTATATTCGGGGATTAAGGGCCTGGGTTGGCTTTCCCCAAACC
GGCGTTAAATTTGAACGGGATCCTCGCCATGCCGGGGAAGTGAAATACACTTTTCGTAAATCTCTCCGTTTAGCCA
TTAACAGTCTGGTTTCCTTTTCCATTGTGCCCC

S. Physical map of the *kaiA*-E103K fragment:

***kaiA*-E103K**

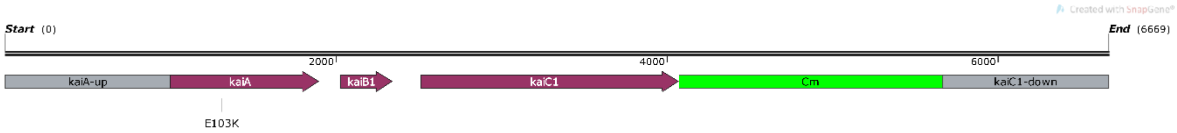

T. DNA sequence of the *kaiA*-E103K fragment, red text highlights the mutation:

TTTAATGCCTGCTCGTAATCCAGCCGTTTAACTGTTTTATTGCCATTTTTCAGCAACTCATTGGCCCGGTTCTGAATC
CATTGGGTCGCTTCCGGTTCCGCCGGGCCACCGGAGGGGGGATTATATTTAAAGCCTCCATCAGTGGGGGGATTG
TGGGAAGGGGTAATAATAATGCCGTCCGCTAAACCTTCTTTACGTCCCTGGTTGTGGGTCAAAATGGCGTAGGAT

ACCGCCGGAGTGGGGGTGAAACGGGTTAAATCCGTGGCGGTGGTTAAAAAAGTTTCTACTTGGTTAGCGGCCAA
CACTTCCAACGCCGTTTTCTGGGCTGGTTCCGACAGAGCATGGCTATCCATCCCCATATAAAGGGGCCCCGTAATG
CCCTGGGCTTGGCGATAGTCCACCACTGCCTGGGTCACCGCCAAAATATGGGCTTCATTAAAAGTACCGTTGAGG
GCAGAACCCCGATGGCCAGAGGTACCAAAGCTCACTAACTGGGCGGGATTTTCCGGGTCCGGTTGCTGACGGTAA
TAGTCGTCTAAAAGTTTGGCCACATCCAAAAGGCTGTCGGCGGGGGGATGCTGGCCGGCGAGGGGATTAATTCT
GCTTGTCATATACAAAAATTGTAAAAAATGGAGGGCGGCGATCAGGGGCTTAGACACCCAAATCCTAGCCAAAAA
GGGTTAACTAGCCAAGGGCTATCCATGGGCAAAGAGATAAAAGAAAAAGTCTCCAAATCCCTGGTCATAGAGAAA
AAATTGCCAAAGTTACCCCAGGCCATACACGGCCCAGCGCCAAGATGGGGAGCACAAATTCAAACTTTGTAAACA
GGCCGGAAGCTATCCGGCCAAGGAGCACTCAGATTGTGTTAACGTTCAGGGGAGTTGCTTAACACAATTTTCCAAT
TAATAGTATTAATATTTTCTTAACTTGCACCGTACCATGGTGAGAAAGCCTATCTGAGCCCTTATTTGATTAACCTTC
GACTGATTATTGATCCCCTGTGCAGTCTCCCCTCTCCCTCTGTCTTTTTGCTCCCGAACACGTTGCCCATAGACTCAG
GTCTATTTTCCAGGGCGATCGCCATTACCTATCGACTTTTCAAGCACTAGATGATTTTTGTGCCTTTCTAGAAGACA
AACCTGAGCGGATTGATTGCCTGTTAGTCTATTACGAAGCTAATTCCCTTCCAGTGCTGAATCGTCTCTATGAACAG
GGGCGATTGTTGCCGATTATTTTGCTCGAACCCAGTCCTTCTGCCCTAGCCAAAACCACCGACGAACACCCCACCAT
TGTCTATCACAACGCTAAAATTCATCTGCCCGAATCCCAATGGTCGGAACTGCCCACCGTCGTAGACCGGGCGA
TCGCCCATTACCTACACCTTGGCCCCATCTGTACCCTCCCCAACCAAACGGAAACTATCCCCGCCCCGATTGTCGAT
GAATCATCCCAAAGCTTTTTACTCCTACAACAAAGAAGGCTGGCTGACAAACTTAAAGAAAGACTCGGTTACCTAG
GAGTGTACTACAAACGTAAGCCCAGTCACTTTTACCGCAACTTTTCCCCCCAGGAAAAACAAGAATACCTAGAAGA
TTTAAGCTCCCAATATCGAGAGATTATTCTCAGTTATTTTAGTGACGAAGGCACAGTTAATGACCTGTTAGATCAAT
TTGTTAATCAGGCTTTCTTTGCCGACCTAGCCATTTCTCAAATCCTGGAAATTCACATGGAATTAATGGATGAATTTT
CCCAGCATCTAAAGCTAGAAGGGCGGAGCGAAGAAGTCCTCCTAGACTATCGTTTAGTGTTGATCGACATCCTCGC
CCATCTGGGGGAAATGTATCGCCGTTCCATCCCCCGGGAGGACATTCCCTTTGATGTATATTATCAGACGGATTAA
TAACCAAATGCTACGGTTTAATCATCAGCTCAACTCTTGGCTGTTGAAACTACCTCGATTCTCTGGTTATCTATTTTC
TTAGTTGTTTCTTGGTTGGTTTCCCCCTCGTTCTAGTAAACCCGTAAAATGAGCCCCTTTAAAAAAACTTACGTTCTC
AAACTCTACGTAGCTGGCAACACCCCCAACTCTGTGCGGGCCTTAAAAATGCTAAAAAATATCCTTGAGCAAGAAT
TCCAGGGAGTTTATGCCCTCAAAGTAATCGACGTGTTGAAAAATCCCCAATTAGCCGAAGAAGATAAAATTCTTGC
CACCCCCACCTTGGCTAAAATCCTACCGCCCCCTGTCAGGAAAATCATCGGCGACCTTTCCGACCGAGAGAAAGTA
TTGATTGGTTTAGACCTGCTCTATGACGAAATTCGGGAACGGGAAGCAGAAGACCAATAGAAAATCGGGGAACA
GGGCGAGATATTTCCCCCATCAACACACATCATCAGACTCCTAACAAACTTTAATCCTGCCCCTCAAACTCAACACA
TTCAATCAGTTTTAGTCAAACTGTTATTTAGTTTTAATTTTACTAACTTTTTTCTTCTTTAAAAATCTAAATTTAGCTAA
TGAACTTACCGATTGTTAACGAACGTAATCGCCCCGATGTGCCAAGGAAGGGAGTGCAAAAAATTCGTACTGTGA
TCGAGGGCTTTGACGAAATTACCCACGGCGGTTTACCCATTGGCCGTACAACCCTGGTGAGTGGCACCTCCGGCAC
AGGCAAAACTCTCTTGGCAGTACAATTTCTTTACCAAGGCATTCACCATTTCGATTATCCGGGTTTATTCATTACATT
TGAAGAATCCCCCAGTGACATTATTGAAAATGCCTATAGTTTTGGCTGGGATTTACAACAATTAATTGACGATGGC
AAATTGTTTATCCTCGATGCTTCCCCCGATCCGGAAGGGCAGGAAGTGGTGGGCACCTTTGATCTGTCGGCCTTAA
TTGAAAGAATTCAGTATGCAGTGCGGAAATATAAAGCCAAGTTAGTTTCCATTGATTCGGTCACAGCGGTATTTCA
ACAATATGATGCGGCTTCGGTGGTGCGGCGGGAAATTTTTCGTTTGGTGGCTAGGTTAAAACAGCTCCAGGTAAC
GTCCATTATGACCACCGAACGGGTGGAAGAATATGGCCCCATTGCCCGCTTTGGCGTAGAGGAATTCGTCTCCGAT
AACGTGGTGGTTTTGCGTAATGTTTTAGAAGGGGAACGGCGACGACGCACGGTGGAAATCCTCAAACTACGGGG
TACCACCCACATGAAGGGGGAATATCCTTTCACTATCACCCACGACGGCATTAACATTTTTCCCCTGGGAGCCATGC
GCCTCACCCAGAGGTCTTCCAATGCCCGCATTTCATCGGGAGTACAAACCTTGGACGAAATGTGTGGCGGTGGCTT
TTTCAAAGATTCGATTATTCTGGCTACGGGGGCTACTGGTACGGGCAAAACCCTGTTGGTAAGCAAATTTTTGCAG
GAAGGTTGTCGCCAAAGAGAACGGGCCATTTTGTTTGCCTATGAGGAATCCAGGGCTCAGCTTTCCCGCAACGCTT
CTTCCTGGGGCATTGATTTTGAAGAAATGGAACACAAGGGTTTATTAAAACTTCTTTGTACCTATCCAGAATCGGC

GGGCTTGGAGGATCATTTGCAAATGATCAAGTCGGAAATATCGGAATTTAAACCTTCCCGCATTGCCATTGATTCC
CTTTCTGCCCTGGCCCGGGGAGTGACCAATAATGCTTTCCGTCAATTTGTCATTGGGGTAACGGGCTACGCCAAAC
AGGAGGAGATTACTGGCTTCTTTACCAATACCACGGACCAATTTATGGGGGCCCATTCCATTACGGAATCCCATAT
TTCCACCATTACAGACACCATTTTGATGTTGCAGTATGTGGAAATCCGAGGAGAAATGTCCCGGGCATTGAATGTG
TTTAAAATGCGGGGTTCCTGGCATGATAAAGGCATTCGAGAATATAGCATTAGCCATGATGGCCCTGATATTCGCG
ATTCCTTCCGCAATTATGAGCGGATTATCAGTGGTTCCCCCACCCGCATTAGTGTGGATGAAAAATCTGAGCTTTCC
CGCATTGTCCGGGGTGTTAAGGACAAGACCGCTGAGTAGAGATCCGTCGATCATATCGTCAATTATTACCTCCACG
GGGAGAGCCTGAGCAAACTGGCCTCAGGCATTTGAGAAGCACACGGTCACACTGCTTCCGGTAGTCAATAAACCG
GTAAACCAGCAATAGACATAAGCGGCTATTTAACGACCCTGCCCTGAACCGACGACCGGGTCGAATTTGCTTTCGA
ATTTCTGCCATTCATCCGCTTATTATCACTTATTCAGGCGTAGCACCAGGCGTTTAAGGGCACCAATAACTGCCTTA
AAAAAATTACGCCCCGCCCTGCCACTCATCGCAGTACTGTTGTAATTCATTAAGCATTCTGCCGACATGGAAGCCAT
CACAAACGGCATGATGAACCTGAATCGCCAGCGGCATCAGCACCTTGTCGCCTTGCGTATAATATTTGCCCATGGT
GAAAACGGGGGCGAAGAAGTTGTCCATATTGGCCACGTTTAAATCAAAACTGGTGAAACTCACCCAGGGATTGGC
TGAGACGAAAAACATATTCTCAATAAACCCTTTAGGGAAATAGGCCAGGTTTTCACCGTAACACGCCACATCTTGC
GAATATATGTGTAGAAACTGCCGGAAATCGTCGTGGTATTCACTCCAGAGCGATGAAAACGTTTCAGTTTGCTCAT
GGAAAACGGTGTAACAAGGGTGAACACTATCCCATATCACCAGCTCACCGTCTTTCATTGCCATACGGAATTCCGG
ATGAGCATTCATCAGGCGGGCAAGAATGTGAATAAAGGCCGGATAAAACTTGTGCTTATTTTTCTTTACGGTCTTT
AAAAAGGCCGTAATATCCAGCTGAACGGTCTGGTTATAGGTACATTGAGCAACTGACTGAAATGCCTCAAAATGTT
CTTTACGATGCCATTGGGATATATCAACGGTGGTATATCCAGTGATTTTTTTCTCCATTTTAGCTTCCTTAGCTCCTG
AAAATCTCGATAACTCAAAAAATACGCCCGGTAGTGATCTTATTTCATTATGGTGAAAGTTGGAACCTCTTACGTGC
CGATCAACGTCTCATTTTCGCCAAAAGTTGGCCCAGGGCTTCCCGGTATCAACAGGGACACCAGGATTTATTTATTC
TGCGAAGTGATCTTCCGTCACAGGTATTTATTCGAAGACGAAAGGGCCTCGTGATACGCCTATTTTTATAGGTTAA
TGTCATGATAATAATGGTTTCTTAGACGTCAGGTGGCACTTTTCGGGGAAATGTGCGCGGAACCCCTATTTGTTTAT
TTTTCTAAATACATTCAAATATGTATCCGCTCATGAGACAATAACCCTGATAAATGCTTCAATAATATTGAAAAAGG
AAGAGTATGAGTATTCAACATTTCCGTGTCGCCCTTATTCCCTTTTTTGCGGCATTTTGCCTTCCTGTTTTTGCTCACC
CAGAAACGCTGGTGAAAGTAAAAGATGCTGAAGATCAGTTGGGTGCACGAGTGGGTTACATCGAACTGGATCTC
AACAGCGGTAAGATCCTTGAGAGTTTTCGCCCCGAAGAACGTTTTCCAATGATGAGCACTTTTAAAGTTCTGCTAT
GTGGCGCGGTATTATCCCGTGTGACGGATCTGATTGAAAAAGGGTAAACTTTCCCATCCGAATCTTGGTTGGGAA
AGGCGGCATGGACAGTGGTTAAGGGTTAGTTTTCTGGGGAATTTAATAACTAAATGTTCCCAGCAAAATCCAGAC
TGATGAGAAGATCGTTAAAGTCTCGATCGCCACCGCCGAATAGATCCTCAACTCCATAAAGGGATTGTCCAAGACG
CATGAAGTGACTTTCGTTATCTGGATTGGCTTGGTCGTACATAAAGAAAGCGTTGACATTGCCTGGTTGATTACCG
GGGTTTTGTTGGAGGAACTGCTCAGTGGTTCCATTGGAGATGAGGAACAAGCCGTAATTACCCCCTGCTTTTAACT
GAGTGGTCGCCGTTTTTTGGGAAAAATTGTCTGGGGCACTAAAGGTGATGCCTCCAGTTAAAGGATCTCGACTCTG
GGTCATGCGAACCGCCGCTTCAGCATAGCCCGCTTGCCCTGGCGAGAAATCGATTATGCCATCGTTATTGATATCA
ATATCCCCATTGGCATTAGCGAGTTTATACAAACCAAGGGTGTTTTGATATTCACCAGCACGATGGATTTGAATATC
AGTGGCGATCGCCTCTCCTTGCAAACCGGGGACAACGAGGGGAAGGGTAAAAACTTCGACGGCATATTTCCCGTT
GGGGGTGTCTGCGGAAATTTGGGTAAAACCCCGACCATCCTGGGTCACTGACAAATTCTCATCCTCAACAGAAAA
CATTTCGTTACTGTTCAAGTCAAGGATATAGGGAGTCAAACTATAAGCATCCTCAATGGTGACCTGGGAAAACGTA
CCGATGTCCTTATGTTCGGTGGAAGCCAGGGAATTACCGGAAGTAAGCACTTGTTGAGCCAGAGTTCGGTTTAAG
GCCAAACCATCCGCCGGAGCGGCTTCCAAAACAGGGCTAAATGAACTCCGTTCACCGTTACGAAAAACCCCGGCTT
GGGTTTCGAGGGGGATAAAAGCTGACCAACCTTCATCGGTGGGATCA

U. Physical map of the *kaiA*-D119E fragment:

***kaiA*-D119E**

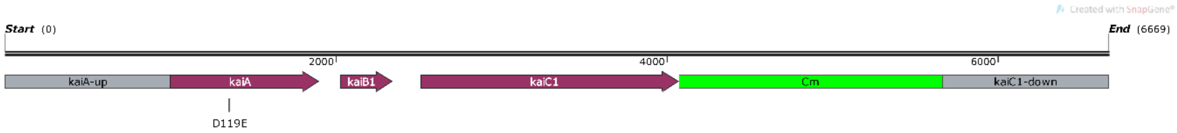

V. DNA sequence of the *kaiA*-D119E fragment, red text highlights the mutation:

TTTAATGCCTGCTCGTAATCCAGCCGTTTAACTGTTTTATTGCCATTTTTCAGCAACTCATTGGCCCGGTTCTGAATC
CATTGGGTCGCTTCCGGTTCCGCCGGGCCACCGGAGGGGGGATTATATTTAAAGCCTCCATCAGTGGGGGGATTG
TGGGAAGGGGTAATAATAATGCCGTCCGCTAAACCTTCTTTACGTCCCTGGTTGTGGGTCAAAATGGCGTAGGAT
ACCGCCGGAGTGGGGGTGAAACGGGTTAAATCCGTGGCGGTGGTTAAAAAAGTTTCTACTTGGTTAGCGGCCAA
CACTTCCAACGCCGTTTTCTGGGCTGGTTCCGACAGAGCATGGCTATCCATCCCCATATAAAGGGGCCCCGTAATG
CCCTGGGCTTGGCGATAGTCCACCACTGCCTGGGTCACCGCCAAAATATGGGCTTCATTAAAAGTACCGTTGAGG
GCAGAACCCCGATGGCCAGAGGTACCAAAGCTCACTAACTGGGCGGGATTTTCCGGGTCCGGTTGCTGACGGTAA
TAGTCGTCTAAAAGTTTGGCCACATCCAAAAGGCTGTCGGCGGGGGGATGCTGGCCGGCGAGGGGATTAATTCT
GCTTGTCATATACAAAAATTGTAAAAAATGGAGGGCGGCGATCAGGGGCTTAGACACCCAAATCCTAGCCAAAAA
GGGTTAACTAGCCAAGGGCTATCCATGGGCAAAGAGATAAAAGAAAAAGTCTCCAAATCCCTGGTCATAGAGAAA
AAATTGCCAAAGTTACCCCAGGCCATACACGGCCCAGCGCCAAGATGGGGAGCACAAATTCAAACTTTGTAAACA
GGCCGGAAGCTATCCGGCCAAGGAGCACTCAGATTGTGTTAACGTTCAGGGGAGTTGCTTAACACAATTTTCCAAT
TAATAGTATTAATATTTTCTTAACTTGCACCGTACCATGGTGAGAAAGCCTATCTGAGCCCTTATTTGATTAACCTTC
GACTGATTATTGATCCCCTGTGCAGTCTCCCCTCTCCCTCTGTCTTTTTGCTCCCGAACACGTTGCCCATAGACTCAG
GTCTATTTTCCAGGGCGATCGCCATTACCTATCGACTTTTCAAGCACTAGATGATTTTTGTGCCTTTCTAGAAGACA
AACCTGAGCGGATTGATTGCCTGTTAGTCTATTACGAAGCTAATTCCCTTCCAGTGCTGAATCGTCTCTATGAACAG
GGGCGATTGTTGCCGATTATTTTGCTCGAACCCAGTCCTTCTGCCCTAGCCAAAACCACCGACGAACACCCCACCAT
TGTCTATCACAACGCTGAAATTCATCTGCCCGAATCCCAATGGTCGGAACTGCCCACCGTCGTA**GAA**CGGGCGA
TCGCCCATTACCTACACCTTGGCCCCATCTGTACCCTCCCCAACCAAACGGAAACTATCCCCGCCCCGATTGTCGAT
GAATCATCCCAAAGCTTTTTACTCCTACAACAAAGAAGGCTGGCTGACAAACTTAAAGAAAGACTCGGTTACCTAG
GAGTGTACTACAAACGTAAGCCCAGTCACTTTTACCGCAACTTTTCCCCCCAGGAAAAACAAGAATACCTAGAAGA
TTTAAGCTCCCAATATCGAGAGATTATTCTCAGTTATTTTAGTGACGAAGGCACAGTTAATGACCTGTTAGATCAAT
TTGTTAATCAGGCTTTCTTTGCCGACCTAGCCATTTCTCAAATCCTGGAAATTCACATGGAATTAATGGATGAATTTT
CCCAGCATCTAAAGCTAGAAGGGCGGAGCGAAGAAGTCCTCCTAGACTATCGTTTAGTGTTGATCGACATCCTCGC
CCATCTGGGGGAAATGTATCGCCGTTCCATCCCCCGGGAGGACATTCCCTTTGATGTATATTATCAGACGGATTAA
TAACCAAATGCTACGGTTTAATCATCAGCTCAACTCTTGGCTGTTGAAACTACCTCGATTCTCTGGTTATCTATTTTC
TTAGTTGTTTCTTGGTTGGTTTCCCCCTCGTTCTAGTAAACCCGTAAAATGAGCCCCTTTAAAAAAACTTACGTTCTC
AAACTCTACGTAGCTGGCAACACCCCCAACTCTGTGCGGGCCTTAAAAATGCTAAAAAATATCCTTGAGCAAGAAT
TCCAGGGAGTTTATGCCCTCAAAGTAATCGACGTGTTGAAAAATCCCCAATTAGCCGAAGAAGATAAAATTCTTGC

CACCCCCACCTTGGCTAAAATCCTACCGCCCCCTGTCAGGAAAATCATCGGCGACCTTTCCGACCGAGAGAAAGTA
TTGATTGGTTTAGACCTGCTCTATGACGAAATTCGGGAACGGGAAGCAGAAGACCAATAGAAAATCGGGGAACA
GGGCGAGATATTTCCCCCATCAACACACATCATCAGACTCCTAACAAACTTTAATCCTGCCCCTCAAACTCAACACA
TTCAATCAGTTTTAGTCAAACTGTTATTTAGTTTTAATTTTACTAACTTTTTTCTTCTTTAAAAATCTAAATTTAGCTAA
TGAACTTACCGATTGTTAACGAACGTAATCGCCCCGATGTGCCAAGGAAGGGAGTGCAAAAAATTCGTACTGTGA
TCGAGGGCTTTGACGAAATTACCCACGGCGGTTTACCCATTGGCCGTACAACCCTGGTGAGTGGCACCTCCGGCAC
AGGCAAAACTCTCTTGGCAGTACAATTTCTTTACCAAGGCATTCACCATTTCGATTATCCGGGTTTATTCATTACATT
TGAAGAATCCCCCAGTGACATTATTGAAAATGCCTATAGTTTTGGCTGGGATTTACAACAATTAATTGACGATGGC
AAATTGTTTATCCTCGATGCTTCCCCCGATCCGGAAGGGCAGGAAGTGGTGGGCACCTTTGATCTGTCGGCCTTAA
TTGAAAGAATTCAGTATGCAGTGCGGAAATATAAAGCCAAGTTAGTTTCCATTGATTCGGTCACAGCGGTATTTCA
ACAATATGATGCGGCTTCGGTGGTGCGGCGGGAAATTTTTCGTTTGGTGGCTAGGTTAAAACAGCTCCAGGTAAC
GTCCATTATGACCACCGAACGGGTGGAAGAATATGGCCCCATTGCCCGCTTTGGCGTAGAGGAATTCGTCTCCGAT
AACGTGGTGGTTTTGCGTAATGTTTTAGAAGGGGAACGGCGACGACGCACGGTGGAAATCCTCAAACTACGGGG
TACCACCCACATGAAGGGGGAATATCCTTTCACTATCACCCACGACGGCATTAACATTTTTCCCCTGGGAGCCATGC
GCCTCACCCAGAGGTCTTCCAATGCCCGCATTTCATCGGGAGTACAAACCTTGGACGAAATGTGTGGCGGTGGCTT
TTTCAAAGATTCGATTATTCTGGCTACGGGGGCTACTGGTACGGGCAAAACCCTGTTGGTAAGCAAATTTTTGCAG
GAAGGTTGTCGCCAAAGAGAACGGGCCATTTTGTTTGCCTATGAGGAATCCAGGGCTCAGCTTTCCCGCAACGCTT
CTTCCTGGGGCATTGATTTTGAAGAAATGGAACACAAGGGTTTATTAAAACTTCTTTGTACCTATCCAGAATCGGC
GGGCTTGGAGGATCATTTGCAAATGATCAAGTCGGAAATATCGGAATTTAAACCTTCCCGCATTGCCATTGATTCC
CTTTCTGCCCTGGCCCGGGGAGTGACCAATAATGCTTTCCGTCAATTTGTCATTGGGGTAACGGGCTACGCCAAAC
AGGAGGAGATTACTGGCTTCTTTACCAATACCACGGACCAATTTATGGGGGCCCATTCCATTACGGAATCCCATAT
TTCCACCATTACAGACACCATTTTGATGTTGCAGTATGTGGAAATCCGAGGAGAAATGTCCCGGGCATTGAATGTG
TTTAAAATGCGGGGTTCCTGGCATGATAAAGGCATTCGAGAATATAGCATTAGCCATGATGGCCCTGATATTCGCG
ATTCCTTCCGCAATTATGAGCGGATTATCAGTGGTTCCCCCACCCGCATTAGTGTGGATGAAAAATCTGAGCTTTCC
CGCATTGTCCGGGGTGTTAAGGACAAGACCGCTGAGTAGAGATCCGTCGATCATATCGTCAATTATTACCTCCACG
GGGAGAGCCTGAGCAAACTGGCCTCAGGCATTTGAGAAGCACACGGTCACACTGCTTCCGGTAGTCAATAAACCG
GTAAACCAGCAATAGACATAAGCGGCTATTTAACGACCCTGCCCTGAACCGACGACCGGGTCGAATTTGCTTTCGA
ATTTCTGCCATTCATCCGCTTATTATCACTTATTCAGGCGTAGCACCAGGCGTTTAAGGGCACCAATAACTGCCTTA
AAAAAATTACGCCCCGCCCTGCCACTCATCGCAGTACTGTTGTAATTCATTAAGCATTCTGCCGACATGGAAGCCAT
CACAAACGGCATGATGAACCTGAATCGCCAGCGGCATCAGCACCTTGTCGCCTTGCGTATAATATTTGCCCATGGT
GAAAACGGGGGCGAAGAAGTTGTCCATATTGGCCACGTTTAAATCAAAACTGGTGAAACTCACCCAGGGATTGGC
TGAGACGAAAAACATATTCTCAATAAACCCTTTAGGGAAATAGGCCAGGTTTTCACCGTAACACGCCACATCTTGC
GAATATATGTGTAGAAACTGCCGGAAATCGTCGTGGTATTCACTCCAGAGCGATGAAAACGTTTCAGTTTGCTCAT
GGAAAACGGTGTAACAAGGGTGAACACTATCCCATATCACCAGCTCACCGTCTTTCATTGCCATACGGAATTCCGG
ATGAGCATTCATCAGGCGGGCAAGAATGTGAATAAAGGCCGGATAAAACTTGTGCTTATTTTTCTTTACGGTCTTT
AAAAAGGCCGTAATATCCAGCTGAACGGTCTGGTTATAGGTACATTGAGCAACTGACTGAAATGCCTCAAAATGTT
CTTTACGATGCCATTGGGATATATCAACGGTGGTATATCCAGTGATTTTTTTCTCCATTTTAGCTTCCTTAGCTCCTG
AAAATCTCGATAACTCAAAAAATACGCCCGGTAGTGATCTTATTTCATTATGGTGAAAGTTGGAACCTCTTACGTGC
CGATCAACGTCTCATTTTCGCCAAAAGTTGGCCCAGGGCTTCCCGGTATCAACAGGGACACCAGGATTTATTTATTC
TGCGAAGTGATCTTCCGTCACAGGTATTTATTCGAAGACGAAAGGGCCTCGTGATACGCCTATTTTTATAGGTTAA
TGTCATGATAATAATGGTTTCTTAGACGTCAGGTGGCACTTTTCGGGGAAATGTGCGCGGAACCCCTATTTGTTTAT
TTTTCTAAATACATTCAAATATGTATCCGCTCATGAGACAATAACCCTGATAAATGCTTCAATAATATTGAAAAAGG
AAGAGTATGAGTATTCAACATTTCCGTGTCGCCCTTATTCCCTTTTTTGCGGCATTTTGCCTTCCTGTTTTTGCTCACC
CAGAAACGCTGGTGAAAGTAAAAGATGCTGAAGATCAGTTGGGTGCACGAGTGGGTTACATCGAACTGGATCTC

AACAGCGGTAAGATCCTTGAGAGTTTTCGCCCCGAAGAACGTTTTCCAATGATGAGCACTTTTAAAGTTCTGCTAT
GTGGCGCGGTATTATCCCGTGTGACGGATCTGATTGAAAAAGGGTAAACTTTCCCATCCGAATCTTGGTTGGGAA
AGGCGGCATGGACAGTGGTTAAGGGTTAGTTTTCTGGGGAATTTAATAACTAAATGTTCCCAGCAAAATCCAGAC
TGATGAGAAGATCGTTAAAGTCTCGATCGCCACCGCCGAATAGATCCTCAACTCCATAAAGGGATTGTCCAAGACG
CATGAAGTGACTTTCGTTATCTGGATTGGCTTGGTCGTACATAAAGAAAGCGTTGACATTGCCTGGTTGATTACCG
GGGTTTTGTTGGAGGAACTGCTCAGTGGTTCCATTGGAGATGAGGAACAAGCCGTAATTACCCCCTGCTTTTAACT
GAGTGGTCGCCGTTTTTTGGGAAAAATTGTCTGGGGCACTAAAGGTGATGCCTCCAGTTAAAGGATCTCGACTCTG
GGTCATGCGAACCGCCGCTTCAGCATAGCCCGCTTGCCCTGGCGAGAAATCGATTATGCCATCGTTATTGATATCA
ATATCCCCATTGGCATTAGCGAGTTTATACAAACCAAGGGTGTTTTGATATTCACCAGCACGATGGATTTGAATATC
AGTGGCGATCGCCTCTCCTTGCAAACCGGGGACAACGAGGGGAAGGGTAAAAACTTCGACGGCATATTTCCCGTT
GGGGGTGTCTGCGGAAATTTGGGTAAAACCCCGACCATCCTGGGTCACTGACAAATTCTCATCCTCAACAGAAAA
CATTTCGTTACTGTTCAAGTCAAGGATATAGGGAGTCAAACTATAAGCATCCTCAATGGTGACCTGGGAAAACGTA
CCGATGTCCTTATGTTCGGTGGAAGCCAGGGAATTACCGGAAGTAAGCACTTGTTGAGCCAGAGTTCGGTTTAAG
GCCAAACCATCCGCCGGAGCGGCTTCCAAAACAGGGCTAAATGAACTCCGTTCACCGTTACGAAAAACCCCGGCTT
GGGTTTCGAGGGGGATAAAAGCTGACCAACCTTCATCGGTGGGATCA

W. Physical map of the *kaiA*-F224S fragment:

***kaiA*-F224S**

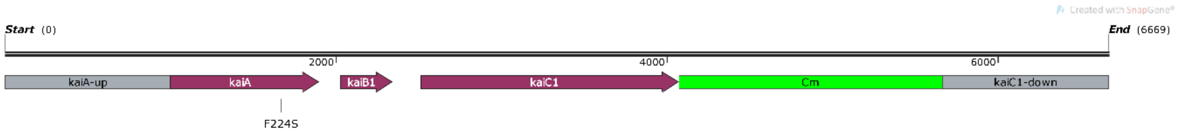

X. DNA sequence of the *kaiA*-F224S fragment, red text highlights the mutation:

TTTAATGCCTGCTCGTAATCCAGCCGTTTAACTGTTTTATTGCCATTTTTCAGCAACTCATTGGCCCGGTTCTGAATC
CATTGGGTCGCTTCCGGTTCCGCCGGGCCACCGGAGGGGGGATTATATTTAAAGCCTCCATCAGTGGGGGGATTG
TGGGAAGGGGTAATAATAATGCCGTCCGCTAAACCTTCTTTACGTCCCTGGTTGTGGGTCAAAATGGCGTAGGAT
ACCGCCGGAGTGGGGGTGAAACGGGTTAAATCCGTGGCGGTGGTTAAAAAAGTTTCTACTTGGTTAGCGGCCAA
CACTTCCAACGCCGTTTTCTGGGCTGGTTCCGACAGAGCATGGCTATCCATCCCCATATAAAGGGGCCCCGTAATG
CCCTGGGCTTGGCGATAGTCCACCACTGCCTGGGTCACCGCCAAAATATGGGCTTCATTAAAAGTACCGTTGAGG
GCAGAACCCCGATGGCCAGAGGTACCAAAGCTCACTAACTGGGCGGGATTTTCCGGGTCCGGTTGCTGACGGTAA
TAGTCGTCTAAAAGTTTGGCCACATCCAAAAGGCTGTCGGCGGGGGGATGCTGGCCGGCGAGGGGATTAATTCT
GCTTGTCATATACAAAAATTGTAAAAAATGGAGGGCGGCGATCAGGGGCTTAGACACCCAAATCCTAGCCAAAAA
GGGTTAACTAGCCAAGGGCTATCCATGGGCAAAGAGATAAAAGAAAAAGTCTCCAAATCCCTGGTCATAGAGAAA
AAATTGCCAAAGTTACCCCAGGCCATACACGGCCCAGCGCCAAGATGGGGAGCACAAATTCAAACTTTGTAAACA

GGCCGGAAGCTATCCGGCCAAGGAGCACTCAGATTGTGTTAACGTTCAGGGGAGTTGCTTAACACAATTTTCCAAT
TAATAGTATTAATATTTTCTTAACTTGCACCGTACCATGGTGAGAAAGCCTATCTGAGCCCTTATTTGATTAACCTTC
GACTGATTATTGATCCCCTGTGCAGTCTCCCCTCTCCCTCTGTCTTTTTGCTCCCGAACACGTTGCCCATAGACTCAG
GTCTATTTTCCAGGGCGATCGCCATTACCTATCGACTTTTCAAGCACTAGATGATTTTTGTGCCTTTCTAGAAGACA
AACCTGAGCGGATTGATTGCCTGTTAGTCTATTACGAAGCTAATTCCCTTCCAGTGCTGAATCGTCTCTATGAACAG
GGGCGATTGTTGCCGATTATTTTGCTCGAACCCAGTCCTTCTGCCCTAGCCAAAACCACCGACGAACACCCCACCAT
TGTCTATCACAACGCTGAAATTCATCTGCCCGAATCCCAATGGTCGGAACTGCCCACCGTCGTAGACCGGGCGATC
GCCCATTACCTACACCTTGGCCCCATCTGTACCCTCCCCAACCAAACGGAAACTATCCCCGCCCCGATTGTCGATGA
ATCATCCCAAAGCTTTTTACTCCTACAACAAAGAAGGCTGGCTGACAAACTTAAAGAAAGACTCGGTTACCTAGGA
GTGTACTACAAACGTAAGCCCAGTCACTTTTACCGCAACTTTTCCCCCCAGGAAAAACAAGAATACCTAGAAGATTT
AAGCTCCCAATATCGAGAGATTATTCTCAGTTATTTTAGTGACGAAGGCACAGTTAATGACCTGTTAGATCAA**TC**
TGTTAATCAGGCTTTCTTTGCCGACCTAGCCATTTCTCAAATCCTGGAAATTCACATGGAATTAATGGATGAATTTT
CCCAGCATCTAAAGCTAGAAGGGCGGAGCGAAGAAGTCCTCCTAGACTATCGTTTAGTGTTGATCGACATCCTCGC
CCATCTGGGGGAAATGTATCGCCGTTCCATCCCCCGGGAGGACATTCCCTTTGATGTATATTATCAGACGGATTAA
TAACCAAATGCTACGGTTTAATCATCAGCTCAACTCTTGGCTGTTGAAACTACCTCGATTCTCTGGTTATCTATTTTC
TTAGTTGTTTCTTGGTTGGTTTCCCCCTCGTTCTAGTAAACCCGTAAAATGAGCCCCTTTAAAAAAACTTACGTTCTC
AAACTCTACGTAGCTGGCAACACCCCCAACTCTGTGCGGGCCTTAAAAAATGCTAAAAAATATCCTTGAGCAAGAAT
TCCAGGGAGTTTATGCCCTCAAAGTAATCGACGTGTTGAAAAATCCCCAATTAGCCGAAGAAGATAAAATTCTTGC
CACCCCCACCTTGGCTAAAATCCTACCGCCCCCTGTCAGGAAAATCATCGGCGACCTTTCCGACCGAGAGAAAGTA
TTGATTGGTTTAGACCTGCTCTATGACGAAATTCGGGAACGGGAAGCAGAAGACCAATAGAAAATCGGGGAACA
GGGCGAGATATTTCCCCCATCAACACACATCATCAGACTCCTAACAAACTTTAATCCTGCCCCTCAAACTCAACACA
TTCAATCAGTTTTAGTCAAACTGTTATTTAGTTTTAATTTTACTAACTTTTTTCTTCTTTAAAAATCTAAATTTAGCTAA
TGAACTTACCGATTGTTAACGAACGTAATCGCCCCGATGTGCCAAGGAAGGGAGTGCAAAAAATTCGTACTGTGA
TCGAGGGCTTTGACGAAATTACCCACGGCGGTTTACCCATTGGCCGTACAACCCTGGTGAGTGGCACCTCCGGCAC
AGGCAAAACTCTCTTGGCAGTACAATTTCTTTACCAAGGCATTCACCATTTCGATTATCCGGGTTTATTCATTACATT
TGAAGAATCCCCCAGTGACATTATTGAAAATGCCTATAGTTTTGGCTGGGATTTACAACAATTAATTGACGATGGC
AAATTGTTTATCCTCGATGCTTCCCCCGATCCGGAAGGGCAGGAAGTGGTGGGCACCTTTGATCTGTCGGCCTTAA
TTGAAAGAATTCAGTATGCAGTGCGGAAATATAAAGCCAAGTTAGTTTCCATTGATTCGGTCACAGCGGTATTTCA
ACAATATGATGCGGCTTCGGTGGTGCGGCGGGAAATTTTTCGTTTGGTGGCTAGGTTAAAACAGCTCCAGGTAAC
GTCCATTATGACCACCGAACGGGTGGAAGAATATGGCCCCATTGCCCGCTTTGGCGTAGAGGAATTCGTCTCCGAT
AACGTGGTGGTTTTGCGTAATGTTTTAGAAGGGGAACGGCGACGACGCACGGTGGAAATCCTCAAACTACGGGG
TACCACCCACATGAAGGGGGAATATCCTTTCACTATCACCCACGACGGCATTAACATTTTTCCCCTGGGAGCCATGC
GCCTCACCCAGAGGTCTTCCAATGCCCGCATTTCATCGGGAGTACAAACCTTGGACGAAATGTGTGGCGGTGGCTT
TTTCAAAGATTCGATTATTCTGGCTACGGGGGCTACTGGTACGGGCAAAACCCTGTTGGTAAGCAAATTTTTGCAG
GAAGGTTGTCGCCAAAGAGAACGGGCCATTTTGTTTGCCTATGAGGAATCCAGGGCTCAGCTTTCCCGCAACGCTT
CTTCCTGGGGCATTGATTTTGAAGAAATGGAACACAAGGGTTTATTAAAACTTCTTTGTACCTATCCAGAATCGGC
GGGCTTGGAGGATCATTTGCAAATGATCAAGTCGGAAATATCGGAATTTAAACCTTCCCGCATTGCCATTGATTCC
CTTTCTGCCCTGGCCCGGGGAGTGACCAATAATGCTTTCCGTCAATTTGTCATTGGGGTAACGGGCTACGCCAAAC
AGGAGGAGATTACTGGCTTCTTTACCAATACCACGGACCAATTTATGGGGGCCCATTCCATTACGGAATCCCATAT
TTCCACCATTACAGACACCATTTTGATGTTGCAGTATGTGGAAATCCGAGGAGAAATGTCCCGGGCATTGAATGTG
TTTAAAATGCGGGGTTCCTGGCATGATAAAGGCATTCGAGAATATAGCATTAGCCATGATGGCCCTGATATTCGCG
ATTCCTTCCGCAATTATGAGCGGATTATCAGTGGTTCCCCCACCCGCATTAGTGTGGATGAAAAATCTGAGCTTTCC
CGCATTGTCCGGGGTGTTAAGGACAAGACCGCTGAGTAGAGATCCGTCGATCATATCGTCAATTATTACCTCCACG

GGGAGAGCCTGAGCAAACTGGCCTCAGGCATTTGAGAAGCACACGGTCACACTGCTTCCGGTAGTCAATAAACCG
GTAAACCAGCAATAGACATAAGCGGCTATTTAACGACCCTGCCCTGAACCGACGACCGGGTCGAATTTGCTTTCGA
ATTTCTGCCATTCATCCGCTTATTATCACTTATTCAGGCGTAGCACCAGGCGTTTAAGGGCACCAATAACTGCCTTA
AAAAAATTACGCCCCGCCCTGCCACTCATCGCAGTACTGTTGTAATTCATTAAGCATTCTGCCGACATGGAAGCCAT
CACAAACGGCATGATGAACCTGAATCGCCAGCGGCATCAGCACCTTGTCGCCTTGCGTATAATATTTGCCCATGGT
GAAAACGGGGGCGAAGAAGTTGTCCATATTGGCCACGTTTAAATCAAAACTGGTGAAACTCACCCAGGGATTGGC
TGAGACGAAAAACATATTCTCAATAAACCCTTTAGGGAAATAGGCCAGGTTTTCACCGTAACACGCCACATCTTGC
GAATATATGTGTAGAAACTGCCGGAAATCGTCGTGGTATTCACTCCAGAGCGATGAAAACGTTTCAGTTTGCTCAT
GGAAAACGGTGTAACAAGGGTGAACACTATCCCATATCACCAGCTCACCGTCTTTCATTGCCATACGGAATTCCGG
ATGAGCATTCATCAGGCGGGCAAGAATGTGAATAAAGGCCGGATAAAACTTGTGCTTATTTTTCTTTACGGTCTTT
AAAAAGGCCGTAATATCCAGCTGAACGGTCTGGTTATAGGTACATTGAGCAACTGACTGAAATGCCTCAAAATGTT
CTTTACGATGCCATTGGGATATATCAACGGTGGTATATCCAGTGATTTTTTTCTCCATTTTAGCTTCCTTAGCTCCTG
AAAATCTCGATAACTCAAAAAATACGCCCGGTAGTGATCTTATTTCATTATGGTGAAAGTTGGAACCTCTTACGTGC
CGATCAACGTCTCATTTTCGCCAAAAGTTGGCCCAGGGCTTCCCGGTATCAACAGGGACACCAGGATTTATTTATTC
TGCGAAGTGATCTTCCGTCACAGGTATTTATTCGAAGACGAAAGGGCCTCGTGATACGCCTATTTTTATAGGTTAA
TGTCATGATAATAATGGTTTCTTAGACGTCAGGTGGCACTTTTCGGGGAAATGTGCGCGGAACCCCTATTTGTTTAT
TTTTCTAAATACATTCAAATATGTATCCGCTCATGAGACAATAACCCTGATAAATGCTTCAATAATATTGAAAAAGG
AAGAGTATGAGTATTCAACATTTCCGTGTCGCCCTTATTCCCTTTTTTGCGGCATTTTGCCTTCCTGTTTTTGCTCACC
CAGAAACGCTGGTGAAAGTAAAAGATGCTGAAGATCAGTTGGGTGCACGAGTGGGTTACATCGAACTGGATCTC
AACAGCGGTAAGATCCTTGAGAGTTTTCGCCCCGAAGAACGTTTTCCAATGATGAGCACTTTTAAAGTTCTGCTAT
GTGGCGCGGTATTATCCCGTGTGACGGATCTGATTGAAAAAGGGTAAACTTTCCCATCCGAATCTTGGTTGGGAA
AGGCGGCATGGACAGTGGTTAAGGGTTAGTTTTCTGGGGAATTTAATAACTAAATGTTCCCAGCAAAATCCAGAC
TGATGAGAAGATCGTTAAAGTCTCGATCGCCACCGCCGAATAGATCCTCAACTCCATAAAGGGATTGTCCAAGACG
CATGAAGTGACTTTCGTTATCTGGATTGGCTTGGTCGTACATAAAGAAAGCGTTGACATTGCCTGGTTGATTACCG
GGGTTTTGTTGGAGGAACTGCTCAGTGGTTCCATTGGAGATGAGGAACAAGCCGTAATTACCCCCTGCTTTTAACT
GAGTGGTCGCCGTTTTTTGGGAAAAATTGTCTGGGGCACTAAAGGTGATGCCTCCAGTTAAAGGATCTCGACTCTG
GGTCATGCGAACCGCCGCTTCAGCATAGCCCGCTTGCCCTGGCGAGAAATCGATTATGCCATCGTTATTGATATCA
ATATCCCCATTGGCATTAGCGAGTTTATACAAACCAAGGGTGTTTTGATATTCACCAGCACGATGGATTTGAATATC
AGTGGCGATCGCCTCTCCTTGCAAACCGGGGACAACGAGGGGAAGGGTAAAAACTTCGACGGCATATTTCCCGTT
GGGGGTGTCTGCGGAAATTTGGGTAAAACCCCGACCATCCTGGGTCACTGACAAATTCTCATCCTCAACAGAAAA
CATTTCGTTACTGTTCAAGTCAAGGATATAGGGAGTCAAACTATAAGCATCCTCAATGGTGACCTGGGAAAACGTA
CCGATGTCCTTATGTTCGGTGGAAGCCAGGGAATTACCGGAAGTAAGCACTTGTTGAGCCAGAGTTCGGTTTAAG
GCCAAACCATCCGCCGGAGCGGCTTCCAAAACAGGGCTAAATGAACTCCGTTCACCGTTACGAAAAACCCCGGCTT
GGGTTTCGAGGGGGATAAAAGCTGACCAACCTTCATCGGTGGGATCA

Y. Physical map of the *kaiC1*-Y402F fragment:

***kaiC1*-Y402F**

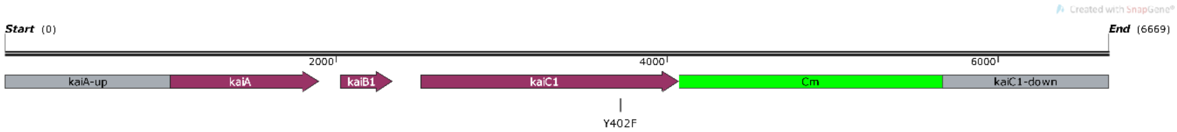

Z. DNA sequence of the *kaiC1*-Y402F fragment, red text highlights the mutation:

TTTAATGCCTGCTCGTAATCCAGCCGTTTAACTGTTTTATTGCCATTTTTCAGCAACTCATTGGCCCGGTTCTGAATC
CATTGGGTCGCTTCCGGTTCCGCCGGGCCACCGGAGGGGGGATTATATTTAAAGCCTCCATCAGTGGGGGGATTG
TGGGAAGGGGTAATAATAATGCCGTCCGCTAAACCTTCTTTACGTCCCTGGTTGTGGGTCAAAATGGCGTAGGAT
ACCGCCGGAGTGGGGGTGAAACGGGTTAAATCCGTGGCGGTGGTTAAAAAAGTTTCTACTTGGTTAGCGGCCAA
CACTTCCAACGCCGTTTTCTGGGCTGGTTCCGACAGAGCATGGCTATCCATCCCCATATAAAGGGGCCCCGTAATG
CCCTGGGCTTGGCGATAGTCCACCACTGCCTGGGTCACCGCCAAAATATGGGCTTCATTAAAAGTACCGTTGAGG
GCAGAACCCCGATGGCCAGAGGTACCAAAGCTCACTAACTGGGCGGGATTTTCCGGGTCCGGTTGCTGACGGTAA
TAGTCGTCTAAAAGTTTGGCCACATCCAAAAGGCTGTCGGCGGGGGGATGCTGGCCGGCGAGGGGATTAATTCT
GCTTGTCATATACAAAAATTGTAAAAAATGGAGGGCGGCGATCAGGGGCTTAGACACCCAAATCCTAGCCAAAAA
GGGTTAACTAGCCAAGGGCTATCCATGGGCAAAGAGATAAAAGAAAAAGTCTCCAAATCCCTGGTCATAGAGAAA
AAATTGCCAAAGTTACCCCAGGCCATACACGGCCCAGCGCCAAGATGGGGAGCACAAATTCAAACTTTGTAAACA
GGCCGGAAGCTATCCGGCCAAGGAGCACTCAGATTGTGTTAACGTTCAGGGGAGTTGCTTAACACAATTTTCCAAT
TAATAGTATTAATATTTTCTTAACTTGCACCGTACCATGGTGAGAAAGCCTATCTGAGCCCTTATTTGATTAACCTTC
GACTGATTATTGATCCCCTGTGCAGTCTCCCCTCTCCCTCTGTCTTTTTGCTCCCGAACACGTTGCCCATAGACTCAG
GTCTATTTTCCAGGGCGATCGCCATTACCTATCGACTTTTCAAGCACTAGATGATTTTTGTGCCTTTCTAGAAGACA
AACCTGAGCGGATTGATTGCCTGTTAGTCTATTACGAAGCTAATTCCCTTCCAGTGCTGAATCGTCTCTATGAACAG
GGGCGATTGTTGCCGATTATTTTGCTCGAACCCAGTCCTTCTGCCCTAGCCAAAACCACCGACGAACACCCCACCAT
TGTCTATCACAACGCTGAAATTCATCTGCCCGAATCCCAATGGTCGGAACTGCCCACCGTCGTAGACCGGGCGATC
GCCCATTACCTACACCTTGGCCCCATCTGTACCCTCCCCAACCAAACGGAAACTATCCCCGCCCCGATTGTCGATGA
ATCATCCCAAAGCTTTTTACTCCTACAACAAAGAAGGCTGGCTGACAAACTTAAAGAAAGACTCGGTTACCTAGGA
GTGTACTACAAACGTAAGCCCAGTCACTTTTACCGCAACTTTTCCCCCCAGGAAAAACAAGAATACCTAGAAGATTT
AAGCTCCCAATATCGAGAGATTATTCTCAGTTATTTTAGTGACGAAGGCACAGTTAATGACCTGTTAGATCAATTTG
TTAATCAGGCTTTCTTTGCCGACCTAGCCATTTCTCAAATCCTGGAAATTCACATGGAATTAATGGATGAATTTTCCC
AGCATCTAAAGCTAGAAGGGCGGAGCGAAGAAGTCCTCCTAGACTATCGTTTAGTGTTGATCGACATCCTCGCCC
ATCTGGGGGAAATGTATCGCCGTTCCATCCCCCGGGAGGACATTCCCTTTGATGTATATTATCAGACGGATTAATA
ACCAAATGCTACGGTTTAATCATCAGCTCAACTCTTGGCTGTTGAAACTACCTCGATTCTCTGGTTATCTATTTTCTT
AGTTGTTTCTTGGTTGGTTTCCCCCTCGTTCTAGTAAACCCGTAAAATGAGCCCCTTTAAAAAAACTTACGTTCTCAA
ACTCTACGTAGCTGGCAACACCCCCAACTCTGTGCGGGCCTTAAAAATGCTAAAAAATATCCTTGAGCAAGAATTC
CAGGGAGTTTATGCCCTCAAAGTAATCGACGTGTTGAAAAATCCCCAATTAGCCGAAGAAGATAAAATTCTTGCCA
CCCCCACCTTGGCTAAAATCCTACCGCCCCCTGTCAGGAAAATCATCGGCGACCTTTCCGACCGAGAGAAAGTATT
GATTGGTTTAGACCTGCTCTATGACGAAATTCGGGAACGGGAAGCAGAAGACCAATAGAAAATCGGGGAACAGG

GCGAGATATTTCCCCCATCAACACACATCATCAGACTCCTAACAAACTTTAATCCTGCCCCTCAAACTCAACACATTC
AATCAGTTTTAGTCAAACTGTTATTTAGTTTTAATTTTACTAACTTTTTTCTTCTTTAAAAATCTAAATTTAGCTAATG
AACTTACCGATTGTTAACGAACGTAATCGCCCCGATGTGCCAAGGAAGGGAGTGCAAAAAATTCGTACTGTGATC
GAGGGCTTTGACGAAATTACCCACGGCGGTTTACCCATTGGCCGTACAACCCTGGTGAGTGGCACCTCCGGCACA
GGCAAAACTCTCTTGGCAGTACAATTTCTTTACCAAGGCATTCACCATTTCGATTATCCGGGTTTATTCATTACATTT
GAAGAATCCCCCAGTGACATTATTGAAAATGCCTATAGTTTTGGCTGGGATTTACAACAATTAATTGACGATGGCA
AATTGTTTATCCTCGATGCTTCCCCCGATCCGGAAGGGCAGGAAGTGGTGGGCACCTTTGATCTGTCGGCCTTAAT
TGAAAGAATTCAGTATGCAGTGCGGAAATATAAAGCCAAGTTAGTTTCCATTGATTCGGTCACAGCGGTATTTCAA
CAATATGATGCGGCTTCGGTGGTGCGGCGGGAAATTTTTCGTTTGGTGGCTAGGTTAAAACAGCTCCAGGTAACG
TCCATTATGACCACCGAACGGGTGGAAGAATATGGCCCCATTGCCCGCTTTGGCGTAGAGGAATTCGTCTCCGATA
ACGTGGTGGTTTTGCGTAATGTTTTAGAAGGGGAACGGCGACGACGCACGGTGGAAATCCTCAAACTACGGGGT
ACCACCCACATGAAGGGGGAATATCCTTTCACTATCACCCACGACGGCATTAACATTTTTCCCCTGGGAGCCATGC
GCCTCACCCAGAGGTCTTCCAATGCCCGCATTTCATCGGGAGTACAAACCTTGGACGAAATGTGTGGCGGTGGCTT
TTTCAAAGATTCGATTATTCTGGCTACGGGGGCTACTGGTACGGGCAAAACCCTGTTGGTAAGCAAATTTTTGCAG
GAAGGTTGTCGCCAAAGAGAACGGGCCATTTTGTTTGCCTATGAGGAATCCAGGGCTCAGCTTTCCCGCAACGCTT
CTTCCTGGGGCATTGATTTTGAAGAAATGGAACACAAGGGTTTATTAAAACTTCTTTGTACCTATCCAGAATCGGC
GGGCTTGGAGGATCATTTGCAAATGATCAAGTCGGAAATATCGGAATTTAAACCTTCCCGCATTGCCATTGATTCC
CTTTCTGCCCTGGCCCGGGGAGTGACCAATAATGCTTTCCGTCAATTTGTCATTGGGGTAACGGGC**TTT**GCCAAA
CAGGAGGAGATTACTGGCTTCTTTACCAATACCACGGACCAATTTATGGGGGCCCATTCCATTACGGAATCCCATA
TTTCCACCATTACAGACACCATTTTGATGTTGCAGTATGTGGAAATCCGAGGAGAAATGTCCCGGGCATTGAATGT
GTTTAAAATGCGGGGTTCCTGGCATGATAAAGGCATTCGAGAATATAGCATTAGCCATGATGGCCCTGATATTCGC
GATTCCTTCCGCAATTATGAGCGGATTATCAGTGGTTCCCCCACCCGCATTAGTGTGGATGAAAAATCTGAGCTTTC
CCGCATTGTCCGGGGTGTTAAGGACAAGACCGCTGAGTAGAGATCCGTCGATCATATCGTCAATTATTACCTCCAC
GGGGAGAGCCTGAGCAAACTGGCCTCAGGCATTTGAGAAGCACACGGTCACACTGCTTCCGGTAGTCAATAAACC
GGTAAACCAGCAATAGACATAAGCGGCTATTTAACGACCCTGCCCTGAACCGACGACCGGGTCGAATTTGCTTTCG
AATTTCTGCCATTCATCCGCTTATTATCACTTATTCAGGCGTAGCACCAGGCGTTTAAGGGCACCAATAACTGCCTT
AAAAAAATTACGCCCCGCCCTGCCACTCATCGCAGTACTGTTGTAATTCATTAAGCATTCTGCCGACATGGAAGCCA
TCACAAACGGCATGATGAACCTGAATCGCCAGCGGCATCAGCACCTTGTCGCCTTGCGTATAATATTTGCCCATGG
TGAAAACGGGGGCGAAGAAGTTGTCCATATTGGCCACGTTTAAATCAAAACTGGTGAAACTCACCCAGGGATTGG
CTGAGACGAAAAACATATTCTCAATAAACCCTTTAGGGAAATAGGCCAGGTTTTCACCGTAACACGCCACATCTTG
CGAATATATGTGTAGAAACTGCCGGAAATCGTCGTGGTATTCACTCCAGAGCGATGAAAACGTTTCAGTTTGCTCA
TGGAAAACGGTGTAACAAGGGTGAACACTATCCCATATCACCAGCTCACCGTCTTTCATTGCCATACGGAATTCCG
GATGAGCATTCATCAGGCGGGCAAGAATGTGAATAAAGGCCGGATAAAACTTGTGCTTATTTTTCTTTACGGTCTT
TAAAAAGGCCGTAATATCCAGCTGAACGGTCTGGTTATAGGTACATTGAGCAACTGACTGAAATGCCTCAAAATGT
TCTTTACGATGCCATTGGGATATATCAACGGTGGTATATCCAGTGATTTTTTTCTCCATTTTAGCTTCCTTAGCTCCT
GAAAATCTCGATAACTCAAAAAATACGCCCGGTAGTGATCTTATTTCATTATGGTGAAAGTTGGAACCTCTTACGT
GCCGATCAACGTCTCATTTTCGCCAAAAGTTGGCCCAGGGCTTCCCGGTATCAACAGGGACACCAGGATTTATTTA
TTCTGCGAAGTGATCTTCCGTCACAGGTATTTATTCGAAGACGAAAGGGCCTCGTGATACGCCTATTTTTATAGGTT
AATGTCATGATAATAATGGTTTCTTAGACGTCAGGTGGCACTTTTCGGGGAAATGTGCGCGGAACCCCTATTTGTT
TATTTTTCTAAATACATTCAAATATGTATCCGCTCATGAGACAATAACCCTGATAAATGCTTCAATAATATTGAAAAA
GGAAGAGTATGAGTATTCAACATTTCCGTGTCGCCCTTATTCCCTTTTTTGCGGCATTTTGCCTTCCTGTTTTTGCTC
ACCCAGAAACGCTGGTGAAAGTAAAAGATGCTGAAGATCAGTTGGGTGCACGAGTGGGTTACATCGAACTGGAT
CTCAACAGCGGTAAGATCCTTGAGAGTTTTCGCCCCGAAGAACGTTTTCCAATGATGAGCACTTTTAAAGTTCTGCT
ATGTGGCGCGGTATTATCCCGTGTGACGGATCTGATTGAAAAAGGGTAAACTTTCCCATCCGAATCTTGGTTGGGA

AAGGCGGCATGGACAGTGGTTAAGGGTTAGTTTTCTGGGGAATTTAATAACTAAATGTTCCCAGCAAAATCCAGA
CTGATGAGAAGATCGTTAAAGTCTCGATCGCCACCGCCGAATAGATCCTCAACTCCATAAAGGGATTGTCCAAGAC
GCATGAAGTGACTTTCGTTATCTGGATTGGCTTGGTCGTACATAAAGAAAGCGTTGACATTGCCTGGTTGATTACC
GGGGTTTTGTTGGAGGAACTGCTCAGTGGTTCCATTGGAGATGAGGAACAAGCCGTAATTACCCCCTGCTTTTAAC
TGAGTGGTCGCCGTTTTTTGGGAAAAATTGTCTGGGGCACTAAAGGTGATGCCTCCAGTTAAAGGATCTCGACTCT
GGGTCATGCGAACCGCCGCTTCAGCATAGCCCGCTTGCCCTGGCGAGAAATCGATTATGCCATCGTTATTGATATC
AATATCCCCATTGGCATTAGCGAGTTTATACAAACCAAGGGTGTTTTGATATTCACCAGCACGATGGATTTGAATAT
CAGTGGCGATCGCCTCTCCTTGCAAACCGGGGACAACGAGGGGAAGGGTAAAAACTTCGACGGCATATTTCCCGT
TGGGGGTGTCTGCGGAAATTTGGGTAAAACCCCGACCATCCTGGGTCACTGACAAATTCTCATCCTCAACAGAAAA
CATTTCGTTACTGTTCAAGTCAAGGATATAGGGAGTCAAACTATAAGCATCCTCAATGGTGACCTGGGAAAACGTA
CCGATGTCCTTATGTTCGGTGGAAGCCAGGGAATTACCGGAAGTAAGCACTTGTTGAGCCAGAGTTCGGTTTAAG
GCCAAACCATCCGCCGGAGCGGCTTCCAAAACAGGGCTAAATGAACTCCGTTCACCGTTACGAAAAACCCCGGCTT
GGGTTTCGAGGGGGATAAAAGCTGACCAACCTTCATCGGTGGGATCA

AA. Physical map of the *kaiC1*-Y402M fragment:

***kaiC1*-Y402M**

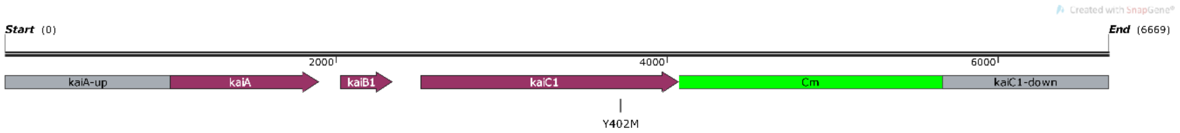

AB. DNA sequence of the *kaiC1*-Y402M fragment, red text highlights the mutation:

TTTAATGCCTGCTCGTAATCCAGCCGTTTAACTGTTTTATTGCCATTTTTCAGCAACTCATTGGCCCGGTTCTGAATC
CATTGGGTCGCTTCCGGTTCCGCCGGGCCACCGGAGGGGGGATTATATTTAAAGCCTCCATCAGTGGGGGGATTG
TGGGAAGGGGTAATAATAATGCCGTCCGCTAAACCTTCTTTACGTCCCTGGTTGTGGGTCAAAATGGCGTAGGAT
ACCGCCGGAGTGGGGGTGAAACGGGTTAAATCCGTGGCGGTGGTTAAAAAAGTTTCTACTTGGTTAGCGGCCAA
CACTTCCAACGCCGTTTTCTGGGCTGGTTCCGACAGAGCATGGCTATCCATCCCCATATAAAGGGGCCCCGTAATG
CCCTGGGCTTGGCGATAGTCCACCACTGCCTGGGTCACCGCCAAAATATGGGCTTCATTAAAAGTACCGTTGAGG
GCAGAACCCCGATGGCCAGAGGTACCAAAGCTCACTAACTGGGCGGGATTTTCCGGGTCCGGTTGCTGACGGTAA
TAGTCGTCTAAAAGTTTGGCCACATCCAAAAGGCTGTCGGCGGGGGGATGCTGGCCGGCGAGGGGATTAATTCT
GCTTGTCATATACAAAAATTGTAAAAAATGGAGGGCGGCGATCAGGGGCTTAGACACCCAAATCCTAGCCAAAAA
GGGTTAACTAGCCAAGGGCTATCCATGGGCAAAGAGATAAAGAAAAAGTCTCCAAATCCCTGGTCATAGAGAAA
AAATTGCCAAAGTTACCCCAGGCCATACACGGCCCAGCGCCAAGATGGGGAGCACAAATTCAAACTTTGTAAACA
GGCCGGAAGCTATCCGGCCAAGGAGCACTCAGATTGTGTTAACGTTCAGGGGAGTTGCTTAACACAATTTTCCAAT
TAATAGTATTAATATTTTCTTAACTTGCACCGTACCATGGTGAGAAAGCCTATCTGAGCCCTTATTTGATTAACCTTC
GACTGATTATTGATCCCCTGTGCAGTCTCCCCTCTCCCTCTGTCTTTTTGCTCCCGAACACGTTGCCCATAGACTCAG
GTCTATTTTCCAGGGCGATCGCCATTACCTATCGACTTTTCAAGCACTAGATGATTTTTGTGCCTTTCTAGAAGACA

AACCTGAGCGGATTGATTGCCTGTTAGTCTATTACGAAGCTAATTCCCTTCCAGTGCTGAATCGTCTCTATGAACAG
GGGCGATTGTTGCCGATTATTTTGCTCGAACCCAGTCCTTCTGCCCTAGCCAAAACCACCGACGAACACCCCACCAT
TGTCTATCACAACGCTGAAATTCATCTGCCCGAATCCCAATGGTCGGAACTGCCCACCGTCGTAGACCGGGCGATC
GCCCATTACCTACACCTTGGCCCCATCTGTACCCTCCCCAACCAAACGGAAACTATCCCCGCCCCGATTGTCGATGA
ATCATCCCAAAGCTTTTTACTCCTACAACAAAGAAGGCTGGCTGACAAACTTAAAGAAAGACTCGGTTACCTAGGA
GTGTACTACAAACGTAAGCCCAGTCACTTTTACCGCAACTTTTCCCCCCAGGAAAAACAAGAATACCTAGAAGATTT
AAGCTCCCAATATCGAGAGATTATTCTCAGTTATTTTAGTGACGAAGGCACAGTTAATGACCTGTTAGATCAATTTG
TTAATCAGGCTTTCTTTGCCGACCTAGCCATTTCTCAAATCCTGGAAATTCACATGGAATTAATGGATGAATTTTCCC
AGCATCTAAAGCTAGAAGGGCGGAGCGAAGAAGTCCTCCTAGACTATCGTTTAGTGTTGATCGACATCCTCGCCC
ATCTGGGGGAAATGTATCGCCGTTCCATCCCCCGGGAGGACATTCCCTTTGATGTATATTATCAGACGGATTAATA
ACCAAATGCTACGGTTTAATCATCAGCTCAACTCTTGGCTGTTGAAACTACCTCGATTCTCTGGTTATCTATTTTCTT
AGTTGTTTCTTGGTTGGTTTCCCCCTCGTTCTAGTAAACCCGTAAAATGAGCCCCTTTAAAAAAACTTACGTTCTCAA
ACTCTACGTAGCTGGCAACACCCCCAACTCTGTGCGGGCCTTAAAAATGCTAAAAAATATCCTTGAGCAAGAATTC
CAGGGAGTTTATGCCCTCAAAGTAATCGACGTGTTGAAAAATCCCCAATTAGCCGAAGAAGATAAAATTCTTGCCA
CCCCCACCTTGGCTAAAATCCTACCGCCCCCTGTCAGGAAAATCATCGGCGACCTTTCCGACCGAGAGAAAGTATT
GATTGGTTTAGACCTGCTCTATGACGAAATTCGGGAACGGGAAGCAGAAGACCAATAGAAAATCGGGGAACAGG
GCGAGATATTTCCCCCATCAACACACATCATCAGACTCCTAACAAACTTTAATCCTGCCCCTCAAACTCAACACATTC
AATCAGTTTTAGTCAAACTGTTATTTAGTTTTAATTTTACTAACTTTTTTCTTCTTTAAAAATCTAAATTTAGCTAATG
AACTTACCGATTGTTAACGAACGTAATCGCCCCGATGTGCCAAGGAAGGGAGTGCAAAAAATTCGTACTGTGATC
GAGGGCTTTGACGAAATTACCCACGGCGGTTTACCCATTGGCCGTACAACCCTGGTGAGTGGCACCTCCGGCACA
GGCAAAACTCTCTTGGCAGTACAATTTCTTTACCAAGGCATTCACCATTTCGATTATCCGGGTTTATTCATTACATTT
GAAGAATCCCCCAGTGACATTATTGAAAATGCCTATAGTTTTGGCTGGGATTTACAACAATTAATTGACGATGGCA
AATTGTTTATCCTCGATGCTTCCCCCGATCCGGAAGGGCAGGAAGTGGTGGGCACCTTTGATCTGTCGGCCTTAAT
TGAAAGAATTCAGTATGCAGTGCGGAAATATAAAGCCAAGTTAGTTTCCATTGATTCGGTCACAGCGGTATTTCAA
CAATATGATGCGGCTTCGGTGGTGCGGCGGGAAATTTTTCGTTTGGTGGCTAGGTTAAAACAGCTCCAGGTAACG
TCCATTATGACCACCGAACGGGTGGAAGAATATGGCCCCATTGCCCGCTTTGGCGTAGAGGAATTCGTCTCCGATA
ACGTGGTGGTTTTGCGTAATGTTTTAGAAGGGGAACGGCGACGACGCACGGTGGAAATCCTCAAACTACGGGGT
ACCACCCACATGAAGGGGGAATATCCTTTCACTATCACCCACGACGGCATTAACATTTTTCCCCTGGGAGCCATGC
GCCTCACCCAGAGGTCTTCCAATGCCCGCATTTCATCGGGAGTACAAACCTTGGACGAAATGTGTGGCGGTGGCTT
TTTCAAAGATTCGATTATTCTGGCTACGGGGGCTACTGGTACGGGCAAAACCCTGTTGGTAAGCAAATTTTTGCAG
GAAGGTTGTCGCCAAAGAGAACGGGCCATTTTGTTTGCCTATGAGGAATCCAGGGCTCAGCTTTCCCGCAACGCTT
CTTCCTGGGGCATTGATTTTGAAGAAATGGAACACAAGGGTTTATTAAAACTTCTTTGTACCTATCCAGAATCGGC
GGGCTTGGAGGATCATTTGCAAATGATCAAGTCGGAAATATCGGAATTTAAACCTTCCCGCATTGCCATTGATTCC
CTTTCTGCCCTGGCCCGGGGAGTGACCAATAATGCTTTCCGTCAATTTGTCATTGGGGTAACGGGC**ATG**GCCAA
ACAGGAGGAGATTACTGGCTTCTTTACCAATACCACGGACCAATTTATGGGGGCCCATTCCATTACGGAATCCCAT
ATTTCCACCATTACAGACACCATTTTGATGTTGCAGTATGTGGAAATCCGAGGAGAAATGTCCCGGGCATTGAATG
TGTTTAAAATGCGGGGTTCCTGGCATGATAAAGGCATTCGAGAATATAGCATTAGCCATGATGGCCCTGATATTCG
CGATTCCTTCCGCAATTATGAGCGGATTATCAGTGGTTCCCCCACCCGCATTAGTGTGGATGAAAAATCTGAGCTTT
CCCGCATTGTCCGGGGTGTTAAGGACAAGACCGCTGAGTAGAGATCCGTCGATCATATCGTCAATTATTACCTCCA
CGGGGAGAGCCTGAGCAAACTGGCCTCAGGCATTTGAGAAGCACACGGTCACACTGCTTCCGGTAGTCAATAAAC
CGGTAAACCAGCAATAGACATAAGCGGCTATTTAACGACCCTGCCCTGAACCGACGACCGGGTCGAATTTGCTTTC
GAATTTCTGCCATTCATCCGCTTATTATCACTTATTCAGGCGTAGCACCAGGCGTTTAAGGGCACCAATAACTGCCT
TAAAAAAATTACGCCCCGCCCTGCCACTCATCGCAGTACTGTTGTAATTCATTAAGCATTCTGCCGACATGGAAGCC
ATCACAAACGGCATGATGAACCTGAATCGCCAGCGGCATCAGCACCTTGTCGCCTTGCGTATAATATTTGCCCATG

GTGAAAACGGGGGCGAAGAAGTTGTCCATATTGGCCACGTTTAAATCAAAACTGGTGAAACTCACCCAGGGATTG
GCTGAGACGAAAAACATATTCTCAATAAACCCTTTAGGGAAATAGGCCAGGTTTTCACCGTAACACGCCACATCTT
GCGAATATATGTGTAGAAACTGCCGGAAATCGTCGTGGTATTCACTCCAGAGCGATGAAAACGTTTCAGTTTGCTC
ATGGAAAACGGTGTAACAAGGGTGAACACTATCCCATATCACCAGCTCACCGTCTTTCATTGCCATACGGAATTCC
GGATGAGCATTCATCAGGCGGGCAAGAATGTGAATAAAGGCCGGATAAAACTTGTGCTTATTTTTCTTTACGGTCT
TTAAAAAGGCCGTAATATCCAGCTGAACGGTCTGGTTATAGGTACATTGAGCAACTGACTGAAATGCCTCAAAATG
TTCTTTACGATGCCATTGGGATATATCAACGGTGGTATATCCAGTGATTTTTTTCTCCATTTTAGCTTCCTTAGCTCCT
GAAAATCTCGATAACTCAAAAAATACGCCCGGTAGTGATCTTATTTCATTATGGTGAAAGTTGGAACCTCTTACGT
GCCGATCAACGTCTCATTTTCGCCAAAAGTTGGCCCAGGGCTTCCCGGTATCAACAGGGACACCAGGATTTATTTA
TTCTGCGAAGTGATCTTCCGTCACAGGTATTTATTCGAAGACGAAAGGGCCTCGTGATACGCCTATTTTTATAGGTT
AATGTCATGATAATAATGGTTTCTTAGACGTCAGGTGGCACTTTTCGGGGAAATGTGCGCGGAACCCCTATTTGTT
TATTTTTCTAAATACATTCAAATATGTATCCGCTCATGAGACAATAACCCTGATAAATGCTTCAATAATATTGAAAAA
GGAAGAGTATGAGTATTCAACATTTCCGTGTCGCCCTTATTCCCTTTTTTGCGGCATTTTGCCTTCCTGTTTTTGCTC
ACCCAGAAACGCTGGTGAAAGTAAAAGATGCTGAAGATCAGTTGGGTGCACGAGTGGGTTACATCGAACTGGAT
CTCAACAGCGGTAAGATCCTTGAGAGTTTTCGCCCCGAAGAACGTTTTCCAATGATGAGCACTTTTAAAGTTCTGCT
ATGTGGCGCGGTATTATCCCGTGTGACGGATCTGATTGAAAAAGGGTAAACTTTCCCATCCGAATCTTGGTTGGGA
AAGGCGGCATGGACAGTGGTTAAGGGTTAGTTTTCTGGGGAATTTAATAACTAAATGTTCCCAGCAAAATCCAGA
CTGATGAGAAGATCGTTAAAGTCTCGATCGCCACCGCCGAATAGATCCTCAACTCCATAAAGGGATTGTCCAAGAC
GCATGAAGTGACTTTCGTTATCTGGATTGGCTTGGTCGTACATAAAGAAAGCGTTGACATTGCCTGGTTGATTACC
GGGGTTTTGTTGGAGGAACTGCTCAGTGGTTCCATTGGAGATGAGGAACAAGCCGTAATTACCCCCTGCTTTTAAC
TGAGTGGTCGCCGTTTTTTGGGAAAAATTGTCTGGGGCACTAAAGGTGATGCCTCCAGTTAAAGGATCTCGACTCT
GGGTCATGCGAACCGCCGCTTCAGCATAGCCCGCTTGCCCTGGCGAGAAATCGATTATGCCATCGTTATTGATATC
AATATCCCCATTGGCATTAGCGAGTTTATACAAACCAAGGGTGTTTTGATATTCACCAGCACGATGGATTTGAATAT
CAGTGGCGATCGCCTCTCCTTGCAAACCGGGGACAACGAGGGGAAGGGTAAAAACTTCGACGGCATATTTCCCGT
TGGGGGTGTCTGCGGAAATTTGGGTAAAACCCCGACCATCCTGGGTCACTGACAAATTCTCATCCTCAACAGAAAA
CATTTCGTTACTGTTCAAGTCAAGGATATAGGGAGTCAAACTATAAGCATCCTCAATGGTGACCTGGGAAAACGTA
CCGATGTCCTTATGTTCGGTGGAAGCCAGGGAATTACCGGAAGTAAGCACTTGTTGAGCCAGAGTTCGGTTTAAG
GCCAAACCATCCGCCGGAGCGGCTTCCAAAACAGGGCTAAATGAACTCCGTTCACCGTTACGAAAAACCCCGGCTT
GGGTTTCGAGGGGGATAAAAGCTGACCAACCTTCATCGGTGGGATCA

AC. Physical map of the *kaiC1*-Y402W fragment:

***kaiC1*-Y402W**

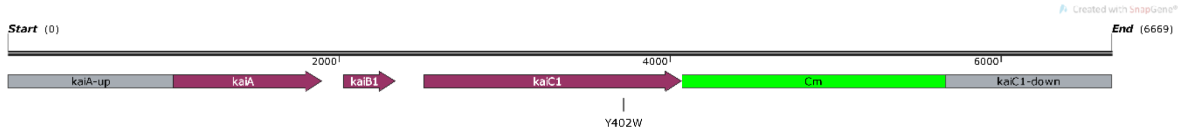

AD. DNA sequence of the *kaiC1*-Y402W fragment, red text highlights the mutation:

TTTAATGCCTGCTCGTAATCCAGCCGTTTAACTGTTTTATTGCCATTTTTCAGCAACTCATTGGCCCGGTTCTGAATC
CATTGGGTCGCTTCCGGTTCCGCCGGGCCACCGGAGGGGGGATTATATTTAAAGCCTCCATCAGTGGGGGGATTG
TGGGAAGGGGTAATAATAATGCCGTCCGCTAAACCTTCTTTACGTCCCTGGTTGTGGGTCAAAATGGCGTAGGAT
ACCGCCGGAGTGGGGGTGAAACGGGTTAAATCCGTGGCGGTGGTTAAAAAAGTTTCTACTTGGTTAGCGGCCAA
CACTTCCAACGCCGTTTTCTGGGCTGGTTCCGACAGAGCATGGCTATCCATCCCCATATAAAGGGGCCCCGTAATG
CCCTGGGCTTGGCGATAGTCCACCACTGCCTGGGTCACCGCCAAAATATGGGCTTCATTAAAAGTACCGTTGAGG
GCAGAACCCCGATGGCCAGAGGTACCAAAGCTCACTAACTGGGCGGGATTTTCCGGGTCCGGTTGCTGACGGTAA
TAGTCGTCTAAAAGTTTGGCCACATCCAAAAGGCTGTCGGCGGGGGGATGCTGGCCGGCGAGGGGATTAATTCT
GCTTGTCATATACAAAAATTGTAAAAAATGGAGGGCGGCGATCAGGGGCTTAGACACCCAAATCCTAGCCAAAAA
GGGTTAACTAGCCAAGGGCTATCCATGGGCAAAGAGATAAAAGAAAAAGTCTCCAAATCCCTGGTCATAGAGAAA
AAATTGCCAAAGTTACCCCAGGCCATACACGGCCCAGCGCCAAGATGGGGAGCACAAATTCAAACTTTGTAAACA
GGCCGGAAGCTATCCGGCCAAGGAGCACTCAGATTGTGTTAACGTTCAGGGGAGTTGCTTAACACAATTTTCCAAT
TAATAGTATTAATATTTTCTTAACTTGCACCGTACCATGGTGAGAAAGCCTATCTGAGCCCTTATTTGATTAACCTTC
GACTGATTATTGATCCCCTGTGCAGTCTCCCCTCTCCCTCTGTCTTTTTGCTCCCGAACACGTTGCCCATAGACTCAG
GTCTATTTTCCAGGGCGATCGCCATTACCTATCGACTTTTCAAGCACTAGATGATTTTTGTGCCTTTCTAGAAGACA
AACCTGAGCGGATTGATTGCCTGTTAGTCTATTACGAAGCTAATTCCCTTCCAGTGCTGAATCGTCTCTATGAACAG
GGGCGATTGTTGCCGATTATTTTGCTCGAACCCAGTCCTTCTGCCCTAGCCAAAACCACCGACGAACACCCCACCAT
TGTCTATCACAACGCTGAAATTCATCTGCCCGAATCCCAATGGTCGGAACTGCCCACCGTCGTAGACCGGGCGATC
GCCCATTACCTACACCTTGGCCCCATCTGTACCCTCCCCAACCAAACGGAAACTATCCCCGCCCCGATTGTCGATGA
ATCATCCCAAAGCTTTTTACTCCTACAACAAAGAAGGCTGGCTGACAAACTTAAAGAAAGACTCGGTTACCTAGGA
GTGTACTACAAACGTAAGCCCAGTCACTTTTACCGCAACTTTTCCCCCCAGGAAAAACAAGAATACCTAGAAGATTT
AAGCTCCCAATATCGAGAGATTATTCTCAGTTATTTTAGTGACGAAGGCACAGTTAATGACCTGTTAGATCAATTTG
TTAATCAGGCTTTCTTTGCCGACCTAGCCATTTCTCAAATCCTGGAAATTCACATGGAATTAATGGATGAATTTTCCC
AGCATCTAAAGCTAGAAGGGCGGAGCGAAGAAGTCCTCCTAGACTATCGTTTAGTGTTGATCGACATCCTCGCCC
ATCTGGGGGAAATGTATCGCCGTTCCATCCCCCGGGAGGACATTCCCTTTGATGTATATTATCAGACGGATTAATA
ACCAAATGCTACGGTTTAATCATCAGCTCAACTCTTGGCTGTTGAAACTACCTCGATTCTCTGGTTATCTATTTTCTT
AGTTGTTTCTTGGTTGGTTTCCCCCTCGTTCTAGTAAACCCGTAAAATGAGCCCCTTTAAAAAAACTTACGTTCTCAA
ACTCTACGTAGCTGGCAACACCCCCAACTCTGTGCGGGCCTTAAAAATGCTAAAAAATATCCTTGAGCAAGAATTC
CAGGGAGTTTATGCCCTCAAAGTAATCGACGTGTTGAAAAATCCCCAATTAGCCGAAGAAGATAAAATTCTTGCCA
CCCCCACCTTGGCTAAAATCCTACCGCCCCCTGTCAGGAAAATCATCGGCGACCTTTCCGACCGAGAGAAAGTATT
GATTGGTTTAGACCTGCTCTATGACGAAATTCGGGAACGGGAAGCAGAAGACCAATAGAAAATCGGGGAACAGG
GCGAGATATTTCCCCCATCAACACACATCATCAGACTCCTAACAAACTTTAATCCTGCCCCTCAAACTCAACACATTC
AATCAGTTTTAGTCAAACTGTTATTTAGTTTTAATTTTACTAACTTTTTTCTTCTTTAAAAATCTAAATTTAGCTAATG
AACTTACCGATTGTTAACGAACGTAATCGCCCCGATGTGCCAAGGAAGGGAGTGCAAAAAATTCGTACTGTGATC
GAGGGCTTTGACGAAATTACCCACGGCGGTTTACCCATTGGCCGTACAACCCTGGTGAGTGGCACCTCCGGCACA
GGCAAAACTCTCTTGGCAGTACAATTTCTTTACCAAGGCATTCACCATTTCGATTATCCGGGTTTATTCATTACATTT
GAAGAATCCCCCAGTGACATTATTGAAAATGCCTATAGTTTTGGCTGGGATTTACAACAATTAATTGACGATGGCA
AATTGTTTATCCTCGATGCTTCCCCCGATCCGGAAGGGCAGGAAGTGGTGGGCACCTTTGATCTGTCGGCCTTAAT
TGAAAGAATTCAGTATGCAGTGCGGAAATATAAAGCCAAGTTAGTTTCCATTGATTCGGTCACAGCGGTATTTCAA
CAATATGATGCGGCTTCGGTGGTGCGGCGGGAAATTTTTCGTTTGGTGGCTAGGTTAAAACAGCTCCAGGTAACG
TCCATTATGACCACCGAACGGGTGGAAGAATATGGCCCCATTGCCCGCTTTGGCGTAGAGGAATTCGTCTCCGATA
ACGTGGTGGTTTTGCGTAATGTTTTAGAAGGGGAACGGCGACGACGCACGGTGGAAATCCTCAAACTACGGGGT

ACCACCCACATGAAGGGGGAATATCCTTTCACTATCACCCACGACGGCATTAACATTTTTCCCCTGGGAGCCATGC
GCCTCACCCAGAGGTCTTCCAATGCCCGCATTTCATCGGGAGTACAAACCTTGGACGAAATGTGTGGCGGTGGCTT
TTTCAAAGATTCGATTATTCTGGCTACGGGGGCTACTGGTACGGGCAAAACCCTGTTGGTAAGCAAATTTTTGCAG
GAAGGTTGTCGCCAAAGAGAACGGGCCATTTTGTTTGCCTATGAGGAATCCAGGGCTCAGCTTTCCCGCAACGCTT
CTTCCTGGGGCATTGATTTTGAAGAAATGGAACACAAGGGTTTATTAAAACTTCTTTGTACCTATCCAGAATCGGC
GGGCTTGGAGGATCATTTGCAAATGATCAAGTCGGAAATATCGGAATTTAAACCTTCCCGCATTGCCATTGATTCC
CTTTCTGCCCTGGCCCGGGGAGTGACCAATAATGCTTTCCGTCAATTTGTCATTGGGGTAACGGGC**TGG**GCCAA
ACAGGAGGAGATTACTGGCTTCTTTACCAATACCACGGACCAATTTATGGGGGCCCATTCCATTACGGAATCCCAT
ATTTCCACCATTACAGACACCATTTTGATGTTGCAGTATGTGGAAATCCGAGGAGAAATGTCCCGGGCATTGAATG
TGTTTAAAATGCGGGGTTCCTGGCATGATAAAGGCATTCGAGAATATAGCATTAGCCATGATGGCCCTGATATTCG
CGATTCCTTCCGCAATTATGAGCGGATTATCAGTGGTTCCCCCACCCGCATTAGTGTGGATGAAAAATCTGAGCTTT
CCCGCATTGTCCGGGGTGTTAAGGACAAGACCGCTGAGTAGAGATCCGTCGATCATATCGTCAATTATTACCTCCA
CGGGGAGAGCCTGAGCAAACTGGCCTCAGGCATTTGAGAAGCACACGGTCACACTGCTTCCGGTAGTCAATAAAC
CGGTAAACCAGCAATAGACATAAGCGGCTATTTAACGACCCTGCCCTGAACCGACGACCGGGTCGAATTTGCTTTC
GAATTTCTGCCATTCATCCGCTTATTATCACTTATTCAGGCGTAGCACCAGGCGTTTAAGGGCACCAATAACTGCCT
TAAAAAAATTACGCCCCGCCCTGCCACTCATCGCAGTACTGTTGTAATTCATTAAGCATTCTGCCGACATGGAAGCC
ATCACAAACGGCATGATGAACCTGAATCGCCAGCGGCATCAGCACCTTGTCGCCTTGCGTATAATATTTGCCCATG
GTGAAAACGGGGGCGAAGAAGTTGTCCATATTGGCCACGTTTAAATCAAAACTGGTGAAACTCACCCAGGGATTG
GCTGAGACGAAAAACATATTCTCAATAAACCCTTTAGGGAAATAGGCCAGGTTTTCACCGTAACACGCCACATCTT
GCGAATATATGTGTAGAAACTGCCGGAAATCGTCGTGGTATTCACTCCAGAGCGATGAAAACGTTTCAGTTTGCTC
ATGGAAAACGGTGTAACAAGGGTGAACACTATCCCATATCACCAGCTCACCGTCTTTCATTGCCATACGGAATTCC
GGATGAGCATTCATCAGGCGGGCAAGAATGTGAATAAAGGCCGGATAAAACTTGTGCTTATTTTTCTTTACGGTCT
TTAAAAAGGCCGTAATATCCAGCTGAACGGTCTGGTTATAGGTACATTGAGCAACTGACTGAAATGCCTCAAAATG
TTCTTTACGATGCCATTGGGATATATCAACGGTGGTATATCCAGTGATTTTTTTCTCCATTTTAGCTTCCTTAGCTCCT
GAAAATCTCGATAACTCAAAAAATACGCCCGGTAGTGATCTTATTTCATTATGGTGAAAGTTGGAACCTCTTACGT
GCCGATCAACGTCTCATTTTCGCCAAAAGTTGGCCCAGGGCTTCCCGGTATCAACAGGGACACCAGGATTTATTTA
TTCTGCGAAGTGATCTTCCGTCACAGGTATTTATTCGAAGACGAAAGGGCCTCGTGATACGCCTATTTTTATAGGTT
AATGTCATGATAATAATGGTTTCTTAGACGTCAGGTGGCACTTTTCGGGGAAATGTGCGCGGAACCCCTATTTGTT
TATTTTTCTAAATACATTCAAATATGTATCCGCTCATGAGACAATAACCCTGATAAATGCTTCAATAATATTGAAAAA
GGAAGAGTATGAGTATTCAACATTTCCGTGTCGCCCTTATTCCCTTTTTTGCGGCATTTTGCCTTCCTGTTTTTGCTC
ACCCAGAAACGCTGGTGAAAGTAAAAGATGCTGAAGATCAGTTGGGTGCACGAGTGGGTTACATCGAACTGGAT
CTCAACAGCGGTAAGATCCTTGAGAGTTTTCGCCCCGAAGAACGTTTTCCAATGATGAGCACTTTTAAAGTTCTGCT
ATGTGGCGCGGTATTATCCCGTGTGACGGATCTGATTGAAAAAGGGTAAACTTTCCCATCCGAATCTTGGTTGGGA
AAGGCGGCATGGACAGTGGTTAAGGGTTAGTTTTCTGGGGAATTTAATAACTAAATGTTCCCAGCAAAATCCAGA
CTGATGAGAAGATCGTTAAAGTCTCGATCGCCACCGCCGAATAGATCCTCAACTCCATAAAGGGATTGTCCAAGAC
GCATGAAGTGACTTTCGTTATCTGGATTGGCTTGGTCGTACATAAAGAAAGCGTTGACATTGCCTGGTTGATTACC
GGGGTTTTGTTGGAGGAACTGCTCAGTGGTTCCATTGGAGATGAGGAACAAGCCGTAATTACCCCCTGCTTTTAAC
TGAGTGGTCGCCGTTTTTTGGGAAAAATTGTCTGGGGCACTAAAGGTGATGCCTCCAGTTAAAGGATCTCGACTCT
GGGTCATGCGAACCGCCGCTTCAGCATAGCCCGCTTGCCCTGGCGAGAAATCGATTATGCCATCGTTATTGATATC
AATATCCCCATTGGCATTAGCGAGTTTATACAAACCAAGGGTGTTTTGATATTCACCAGCACGATGGATTTGAATAT
CAGTGGCGATCGCCTCTCCTTGCAAACCGGGGACAACGAGGGGAAGGGTAAAAACTTCGACGGCATATTTCCCGT
TGGGGGTGTCTGCGGAAATTTGGGTAAAACCCCGACCATCCTGGGTCACTGACAAATTCTCATCCTCAACAGAAAA
CATTTCGTTACTGTTCAAGTCAAGGATATAGGGAGTCAAACTATAAGCATCCTCAATGGTGACCTGGGAAAACGTA
CCGATGTCCTTATGTTCGGTGGAAGCCAGGGAATTACCGGAAGTAAGCACTTGTTGAGCCAGAGTTCGGTTTAAG

GCCAAACCATCCGCCGGAGCGGCTTCCAAAACAGGGCTAAATGAACTCCGTTCACCGTTACGAAAAACCCCGGCTT
GGGTTTCGAGGGGGATAAAAGCTGACCAACCTTCATCGGTGGGATCA

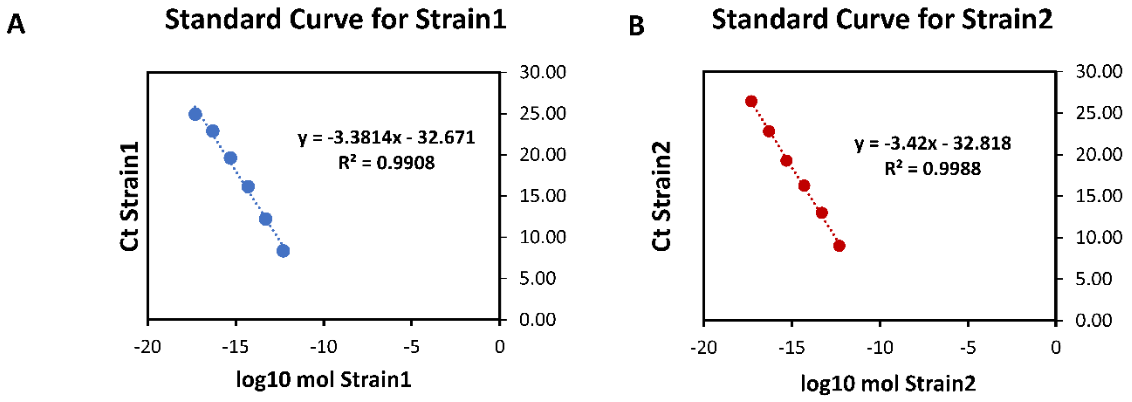

Supplemental Figure S5. The percentages of 2 strains in strain mixtures were quantified through absolute quantitative PCR (QPCR). We first amplified the specific genome regions (150bp for each) of Strain1 (WT in this figure) and Strain2 (kaiAB1C1-ko in this figure) through regular PCR, purified the DNA fragments, and measured the concentrations respectively. Each PCR fragment was diluted to different concentrations (for example at 10^{-13} mol/µl, 10^{-14}mol/ µl, 10^{-15}mol/ µl, 10^{-16}mol/ µl, 10^{-17}mol/ µl and 10^{-18}mol/ µl). QPCR for DNA fragments of the 2 specific regions (based on the different antibiotic resistance genes) was performed with known DNA concentrations to obtain the Ct values. With the Ct values from QPCR, standard curves for the correlation between log10 mol Strain1 with Ct Strain1, and log10 mol Strain2 with Ct Strain2 were plotted as shown, and these served as our standard curves to calculate the chromosome copy numbers from the real samples of mixed-strain cultures. Panels A and B are examples of the standard curves (in this particular example, WT and kaiAB1C1-ko). With real experimental samples (e.g., co-cultures of Strain1 and Strain2), the same sample was run separately with Strain1 specific primers and Strain2 specific primers, based on the antibiotic resistance cassettes described for each strain in the Materials and Methods. From the Ct values for both strains, the mol for each strain in the co-culture could be quantified separately, and thereby the percentage of each strain based on mol amounts could be calculated. For each 96 well PCR plate, both the samples for standard curves and for the real experiments were always included in the same plate. Therefore, the experimental samples were analyzed simultaneously with the standard curve samples to avoid any batch errors.
